# Diversity and relative abundance of ammonia- and nitrite-oxidizing microorganisms in the offshore Namibian hypoxic zone

**DOI:** 10.1371/journal.pone.0217136

**Published:** 2019-05-21

**Authors:** Evan Lau, Caitlin H. Frame, E. Joseph Nolan, Frank J. Stewart, Zachary W. Dillard, Daniel P. Lukich, Nicole E. Mihalik, Katelyn E. Yauch, Marcus A. Kinker, Samantha L. Waychoff

**Affiliations:** 1 Department of Biology, Menlo College, Atherton, California, United States of America; 2 Department of Natural Sciences and Mathematics, West Liberty University, West Liberty, West Virginia, United States of America; 3 Department of Environmental Sciences, University of Basel, Basel, Switzerland; 4 School of Biological Sciences, Georgia Institute of Technology, Atlanta, Georgia, United States of America; Hong Kong University of Science and Technology, HONG KONG

## Abstract

Nitrification, the microbial oxidation of ammonia (NH_3_) to nitrite (NO_2_^–^) and NO_2_^–^ to nitrate (NO_3_^–^), plays a vital role in ocean nitrogen cycling. Characterizing the distribution of nitrifying organisms over environmental gradients can help predict how nitrogen availability may change with shifting ocean conditions, for example, due to loss of dissolved oxygen (O_2_). We characterized the distribution of nitrifiers at 5 depths spanning the oxic to hypoxic zone of the offshore Benguela upwelling system above the continental slope off Namibia. Based on 16S rRNA gene amplicon sequencing, the proportional abundance of nitrifiers (ammonia and nitrite oxidizers) increased with depth, driven by an increase in ammonia-oxidizing archaea (AOA; Thaumarchaeota) to up to 33% of the community at hypoxic depths where O_2_ concentrations fell to ~25 μM. The AOA community transitioned from being dominated by a few members at oxic depths to a more even representation of taxa in the hypoxic zone. In comparison, the community of NO_2_^–^-oxidizing bacteria (NOB), composed primarily of Nitrospinae, was far less abundant and exhibited higher evenness at all depths. The AOA:NOB ratio declined with depth from 41:1 in the oxic zone to 27:1 under hypoxia, suggesting potential variation in the balance between NO_2_^–^ production and consumption via nitrification. Indeed, in contrast to prior observations from more O_2_-depleted sites closer to shore, NO_2_^–^ did not accumulate at hypoxic depths near this offshore site, potentially due in part to a tightened coupling between AOA and NOB.

## Introduction

Microbial nitrification plays an important role in regulating the availability of nitrogen (N) for biological consumption. In the first step of nitrification, ammonia (NH_3_) is oxidized to nitrite (NO_2_^−^) by ammonia-oxidizing archaea (AOA) or bacteria (AOB), with AOA of the phylum Thaumarchaeota playing a major role in NH_3_ oxidation in marine systems [1–3]. In the second nitrification step, NO_2_^−^ is oxidized to nitrate (NO_3_^–^) by nitrite-oxidizing bacteria (NOB), with members of the *Nitrospina* (Nitrospinae), *Nitrococcus* (Gammaproteobacteria), and *Nitrospira* (Nitrospirae) among the most common NOB in marine systems [4–8]. The microbes mediating these two steps can be spatially separated or active at different times based on environmental conditions, including the concentrations of inorganic N substrates and dissolved O_2_ [e.g., 9–11]. Alternatively, AOA/AOB and NOB activity can be tightly coupled, with no NO_2_^−^ accumulation [e.g., 11,12]. Characterizing the distribution of nitrifying taxa in diverse habitats, such as sites with low dissolved O_2_ concentrations, can help determine the physical or chemical thresholds that decouple NH_3_ oxidation from NO_2_^−^ oxidation, and therefore help predict the accumulation and flux of inorganic N in ocean systems.

Nitrifying microorganisms are abundant in and at the periphery of marine zones where dissolved O_2_ concentrations decline, including the upwelling-driven oxygen minimum zones (OMZs) of the eastern Pacific [13,14] and the Benguela upwelling system off Namibia [7]. At the core of these zones, dissolved O_2_ concentration is in the low nanomolar (nM) range [15,16]. Anaerobic metabolisms dominate under these conditions. Such metabolisms include both denitrification, which begins with the NO_2_^−^-producing step of NO_3_^–^ reduction, as well as anaerobic ammonia oxidation (anammox) with NO_2_^−^, a process that may compete with NOB for NO_2_^−^. These anaerobic processes, however, are substantially suppressed as dissolved O_2_ rises to levels of just a few micromolar (μM), which are sufficient to concurrently enable a range of aerobic metabolisms [12,17]. Notably, in the transition zone from near-anoxia to hypoxia (a few to tens of μM O_2_), low O_2_-adapted nitrifiers make substantial contributions to N cycling [13,14,18,19]. In this zone, coupling between the two nitrification steps may be a primary control over the availability and accumulation of inorganic N intermediates that feed both oxidative and reductive metabolisms, including NO_2_^–^ and the greenhouse gas nitrous oxide (N_2_O) [11,13,20–22]. Thus, there is considerable interest in understanding nitrifier abundance, diversity, and activity as O_2_ concentration declines [7,13,23].

The Benguela upwelling system is an ideal model for exploring nitrifier community structure over varying O_2_ regimes. In this system, coastal upwelling of nutrient-rich water extends from the southern tip of Africa (~35°S) to the Angola front (~15°S), reaching peak magnitude off Namibia [24]. The resulting high levels of primary production drive O_2_ depletion by microbial respiration at mid-water depths below the euphotic zone [25–27]. Here, shallow (< 300 m depth) near-shore waters above the continental shelf may be highly O_2_-depleted (O_2_ < 5 μM), while offshore waters above the continental slope experience milder subsurface hypoxia (O_2_ < 60 μM) [7,12,28,30].

Such variation in O_2_ levels may influence the coupling of NO_2_^–^ production and consumption. In an analysis of offshore, deeper Benguela sites (seabed depth > 1,000 m at most sites), Mashifane et al. (2016) detected a primary NO_2_^–^ maximum only in the oxic euphotic zone (30–50 m), which was attributed to a decoupling of nitrification steps and/or microbial assimilatory NO_3_^–^ reduction to NO_2_^–^, but did not detect a secondary NO_2_^–^ maximum at deeper depths. In contrast, near-shore sites (seabed depth ~100 m to ~300 m) on the Namibia shelf exhibit NO_2_^–^ maxima at hypoxic (generally defined as ≤ ~60–120 μM [31]) or anoxic depths (where O_2_ is near or below detection limits, ~10 nM to 1–2 μM depending on sensor technology), typically below ~50–80 m depth [7,12,23,29], a feature common in O_2_-deficient waters where high rates of NO_3_^–^ reduction drive NO_2_^–^ accumulation [18,26,32]. At these near-shore sites, NO_2_^–^ oxidation rates have been shown to exceed those of NH_3_ oxidation, and NOB, roughly evenly represented by the genera *Nitrospina* and *Nitrococcus*, may constitute up to 10% of the total community [7]. These results, alongside those of others [29], suggest active nitrification in near-shore waters above the Namibian shelf, with the balance of NO_2_^–^ also strongly influenced by anaerobic metabolisms, including NO_2_^–^-consuming anammox.

It remains less clear how nitrifier community composition and coupling of NO_2_^–^ consumption and production by nitrifiers change upon the transition to the O_2_-enriched (but still hypoxic) waters further offshore in the Benguela system. Studies in other systems indicate that AOA distributions and NH_3_ oxidation rates are influenced by nutrient and O_2_ concentrations [33–35], and that AOA communities in the Arabian Sea OMZ are distinct from those of OMZs of the Eastern Pacific [36]. A study of relatively O_2_-rich waters in Monterey Bay and the North Pacific Subtropical Gyre showed that the ratio of AOA to NOB ranged from 5:1 to 1:1 [37], suggesting a coupling of ammonia oxidizers and NOB. Similarly, a study in oxic waters of the Gulf of Mexico found that AOA outnumbered NOB by 20 to 1 [38]. The relative abundances of these groups were correlated and secondary NO_2_^–^ maxima were not observed, which together was interpreted as a coupling between nitrifiers [38]. In contrast, Bristow et al. (2015) reported NO_2_^–^ accumulation in hypoxic waters (~16–60 μM), while recording a similarly disproportionate relationship between AOA and NOB abundance, and a decoupling of NH_3_ and NO_2_^–^ oxidation rates at sites spanning hypoxic to oxic conditions on the Louisiana Shelf. Such studies indicate that nitrifier community structuring is dynamic based on environment and geography, and our current limited understanding of whether the relative abundances of ammonia and nitrite oxidizers are predictive of nitrifier coupling.

To explore variation in nitrifier community structuring and coupling, we assessed the relative abundances, phylogenetic diversity, and co-occurrence of nitrifying taxa offshore of Namibia. Using 16S rRNA amplicon gene sequencing, we analyzed the nitrifier community at five depths spanning oxic to hypoxic conditions, while measuring temperature and O_2_, NO_2_^–^, and N_2_O concentrations. We hypothesized that ammonia and nitrite oxidizer diversity changes with depth and decreasing O_2_ concentrations, NOB community composition differs from that previously described at more O_2_-depleted sites close to shore, and ammonia oxidizer to NOB ratios help predict NO_2_^–^ accumulation. Testing these hypotheses sheds light on the environmental drivers of nitrifier community structure and how these communities may influence N availability.

## Materials and methods

### Site description, geochemical profiles, seawater sample collection

**Fig 1:**
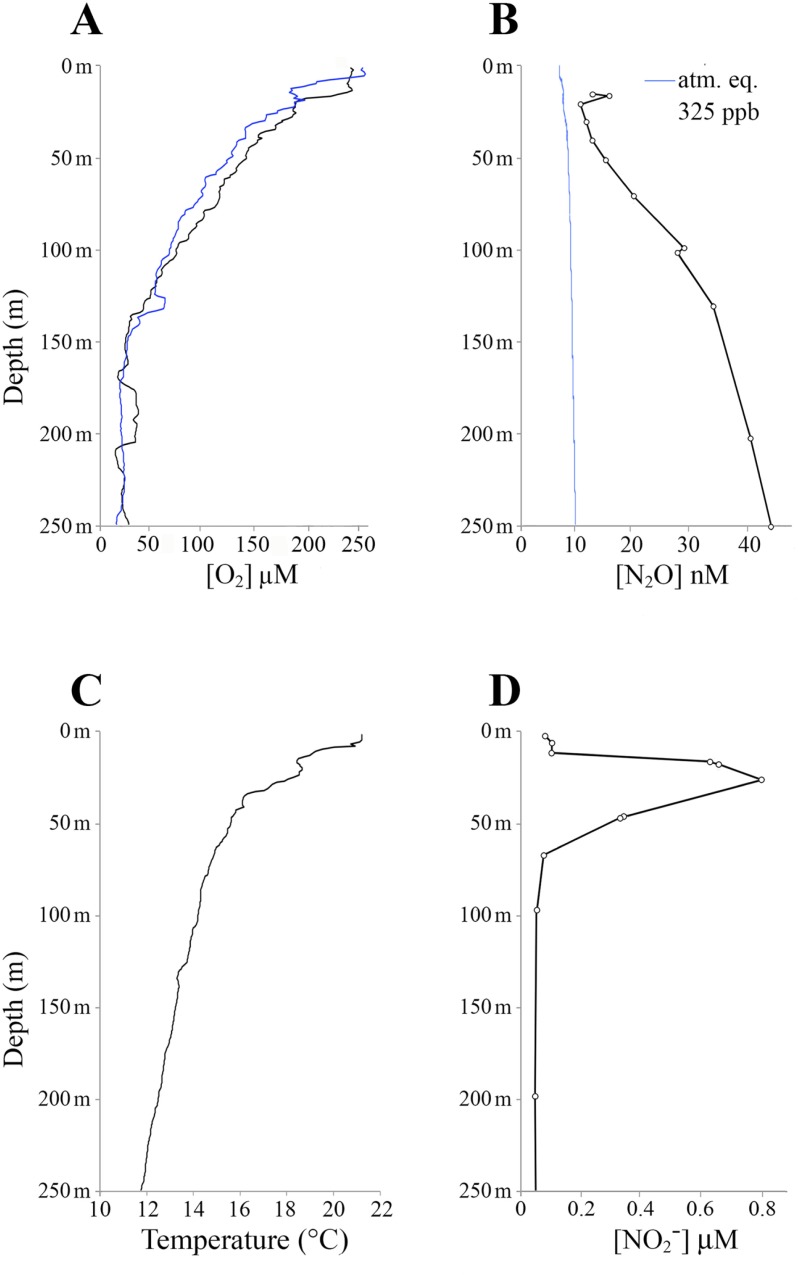
Environmental conditions at the study sites in January 2014. **(A) O**_**2**_ **concentration at stations 116 (blue line) and 117 (black line), (B) N**_**2**_**O concentration at station 116 (black line), with concentration of dissolved N**_**2**_**O at equilibrium (blue line), (C) temperature at station 116, and (D) NO**_**2**_^**−**^ **concentration at station 117.** Microbial samples were collected at station 116. Stations 116 and 117 are separated by ~10 km.

Seawater samples from the Northern Benguela Upwelling system off the Namibian coast were collected during cruise M-103 (“NAMUFil”; Dec. 27, 2013 –Feb. 11, 2014) of the R/V *Meteor*. All cruise operations were conducted in accordance with the Namibian Ministry of Foreign Affairs and Ministry of Mines and Energy. No additional permissions were required for seawater sampling. Samples for microbial analysis were collected on January 31, 2014 at station 116 (20.172°S, 11.540°E) over the continental slope (water depth ~865 m) approximately 150 km from the coast. Seawater was obtained from depths of 10, 25, 100, 130, and 250 m using a rosette equipped with a conductivity, temperature, and depth sensor (CTD), fluorometer (Wet Labs ECO-AFL/FL), and SBE 9 dissolved oxygen sensor (Sea-Bird Scientific). Four liters of water per depth (station 116) were filtered through a 0.22 μm-pore size 47 mm polycarbonate Nuclepore filter (Whatman). Filters were frozen immediately and stored at −80°C. Using water from a separate hydrocast on January 31, 2014, samples for measuring N_2_O were collected from 15 depths (17 to 250 m; Fig 1B). For each, water was twice overfilled into 160 ml glass bottles and preserved by adding 5 ml of 10 M sodium hydroxide [39], followed by vigorous shaking. Within 3 months of collection, total N_2_O in each bottle (one per depth) was purged with carrier helium into a purge- and-trap system [40] and analyzed by continuous-flow gas chromatography-isotope ratio mass spectrometry (GC-IRMS) was previously described [41], with atmospheric equilibrium N_2_O concentrations calculated using the solubility coefficients of Weiss and Price (1980), assuming a 325 ppb atmospheric mole fraction [42]. NO_2_^−^ concentrations were not measured at station 116. However, NO_2_^−^ was measured in samples (12 depths; 0 to 300 m; Fig 1D) collected on February 1, 2014 from station 117 (20.204°S, 11.444°E), approximately 10 km west-southwest of station 116. NO_2_^−^ in 10 ml of sample was measured by converting NO_2_^-^ to N_2_O by azide reduction [43], followed by N_2_O measurement via GC-IRMS as above. Calculations of N_2_O and NO_2_^−^ concentrations were previously described [41].

### 16S rRNA gene amplicon sequencing

The composition of the prokaryotic microbial community was assessed via 16S rRNA gene amplicon sequencing. Briefly, DNA was extracted using the FastDNA spin kit for soil (MP Biomedicals) following the manufacturer’s protocol. The polymerase chain reaction was used to amplify the V4 region of the 16S rRNA gene using barcoded universal primers F515 (5′- GTGCCAGCMGCCGCGGTAA-3′) and R806 (5′-GGACTACHVGGGTWTCTAAT-3′) [44] appended with Illumina-specific adapters [45], with PCR run for 30 cycles using previously described thermal cycler protocol [46]. Three replicate PCR amplifications (different barcodes) were conducted for each of the five depths (15 total reactions). Gel electrophoresis was used to separate PCR products, which were then excised and recovered using a QIAquick gel extraction kit (Qiagen). The amplicon pool was then sequenced on an Illumina MiSeq at the West Virginia University Genomics Core Facility using V2 sequencing reagents, generating paired reads of ∼250 bp, with ∼150 bp overlap between forward and reverse reads.

### Bioinformatic analyses

Paired read merging, quality filtering and denoising, and assignment and analysis of operational taxonomic units (OTUs) were carried out using PANDAseq in Axiome [47,48] and the Quantitative Insights Into Microbial Ecology (QIIME v.1.8.0) pipeline (Caporaso et al., 2010). In PANDAseq, the minimum overlap length was set at 0.9 and the read length maximum was set at 253 bp. Reads were removed from analysis if at least one of the following criteria was met: reads were > 4 bp shorter than the maximum (253 bp), the number of ambiguous bases was > 1, homopolymers with > 4 bp were present, or sequences did not match any sequences in the database at > 97% coverage via BLAST [49]. In QIIME, sequences were clustered into OTUs at 97% sequence identity using UCLUST [50,51], with OTU taxonomic classification done using the RDP classifier [52] at a confidence threshold of 70% and Greengenes datafiles gg_13_8 compiled in May 2013 (http://greengenes.lbl.gov). This step included chimera screening based on 16S rRNA genes in GenBank [53]. All OTUs matching known AOA, AOB, NOB, and MOB (methane oxidizing bacteria) were retrieved and their taxonomic assignments further verified via BLASTN against the NCBI 16S rRNA sequence database (for Bacteria + Archaea).

### Phylogenetic analysis of 16S rRNA sequences

Phylogenetic trees were constructed using MEGA 7 [54]. The 16S rRNA gene sequences of representative AOA, AOB, NOB, and MOB were downloaded from GenBank [55]. To verify that 16S rRNA gene sequences were indeed nitrifiers or MOB, sequences were removed if they shared ≤ 80% identity with known AOA, AOB, NOB, and MOB based on BLAST search. Gene sequences were aligned using MUSCLE in MEGA 7 and manually inspected. The final alignments for AOA, AOB, NOB, and MOB comprised 794, 36, 338, and 27 taxa, respectively. Phylogenetic reconstruction was implemented using Maximum Likelihood (ML), employing the Tamura-Nei model of nucleotide substitution rate, with tree inference based on Nearest-Neighbor-Interchange. Statistical support for ML trees was obtained from 1,000 bootstrap replicates under the initial settings (only bootstrap values > 50% are reported). Rarefaction curves using only ammonia oxidizer (AOA+AOB) OTUs and NOB OTUs were generated using the vegan package in R [56] to estimate nitrifier species richness.

### Statistical and ecological analyses, diversity indices

Nitrifier and MOB OTU frequencies (proportional abundances) were determined using a Python (v.2.7) script to count all OTUs verified by the above phylogenetic analyses, as previously described [41,57]. The Shannon-Weiner diversity and Pielou (J) evenness indices were calculated using the BiodiversityR package [58] in R version 3.4.1 (http://www.R-project.org/). Correlation between nitrifier (AOA+AOB and NOB) diversity indices or OTU abundances and geochemical measurements (O_2_, NO_2_^−^, N_2_O concentrations, and temperature) at all 5 depths were calculated in R using the adjusted R-squared coefficient of determination.

To identify nitrifier OTUs that co-occurred at the same depth, hierarchical cluster analysis was performed using a Euclidean distance matrix based on ammonia oxidizer and NOB frequencies at each depth (after omitting depths where any given OTU occurred only once), generated in R version 3.5.0 (https://CRAN.R-project.org/package=ggdendro). Using this distance matrix and the "agnes" function in the "cluster" package (https://cran.r-project.org/web/packages/cluster/index.html), agglomerative clustering was performed using Ward's minimum variance method, chosen to minimize overall within-cluster variance. The results were visualized using the packages "ggplot2" [59] and "ggdendro" [60].

### Project and sequence accession numbers

All sequence data are available through the European Bioinformatics Institute (EBI) under project accession number PRJEB21239 and individual accession numbers LT896734–LT897482 for ammonia-oxidizing microbes, LT897483–897776 and LT900516 for NOB, and LT900504–900514 for MOB (S1–S4 Tables).

## Results

### Geochemical profiles

Dissolved O_2_ concentration at station 116 declined from 252 μM at the surface to 64 μM (or ~2 mg L^-1^, the beginning of hypoxia) at 105 m depth, declining to a minimum of ~20–25 μM from ~170 m to 370 m (Fig 1A, S1 Fig). Below 370 m, O_2_ concentration increased gradually to ~120 μM just above the sediment-water interface (S1 Fig). N_2_O concentration increased steadily from ~10–20 nM in the oxic zone to > 30 nM at hypoxic depths below 80 m, peaking at ~40 nM at 250 m (Fig 1B). Seawater temperature declined from 21°C at the surface to 12°C at 250 m (Fig 1C). Fluorescence peaked at ~3.9 mg m^-3^ at 10 m and declined to near zero by 80 m (S2 Fig). NO_2_^−^ was not measured at station 116. At station 117, ~10 km from station 116 and with similar O_2_ concentration profile as station 116 (Fig 1A), NO_2_^−^ concentration exhibited a primary maximum (up to 0.8 μM) between 20 m and 50 m, and was consistently low (< 0.1 μM) in the hypoxic zone (Fig 1D), which occurred at roughly the same depths as at station 116; a secondary NO_2_^−^ maximum was not observed.

Counts of 16S rRNA gene amplicons (after quality filtering) ranged from 9,681 to 945,184 across the 15 datasets (5 depths X 3 triplicate PCR reactions per depth). Average OTU counts per dataset ranged from 4,912 to 8,312. At this sequence depth, the observed OTU counts represented 20.0%–60.7% (mean = 47.8%) of the OTU counts estimated via Chao1 (see rarefaction curves in S3 Fig).

**Fig 2:**
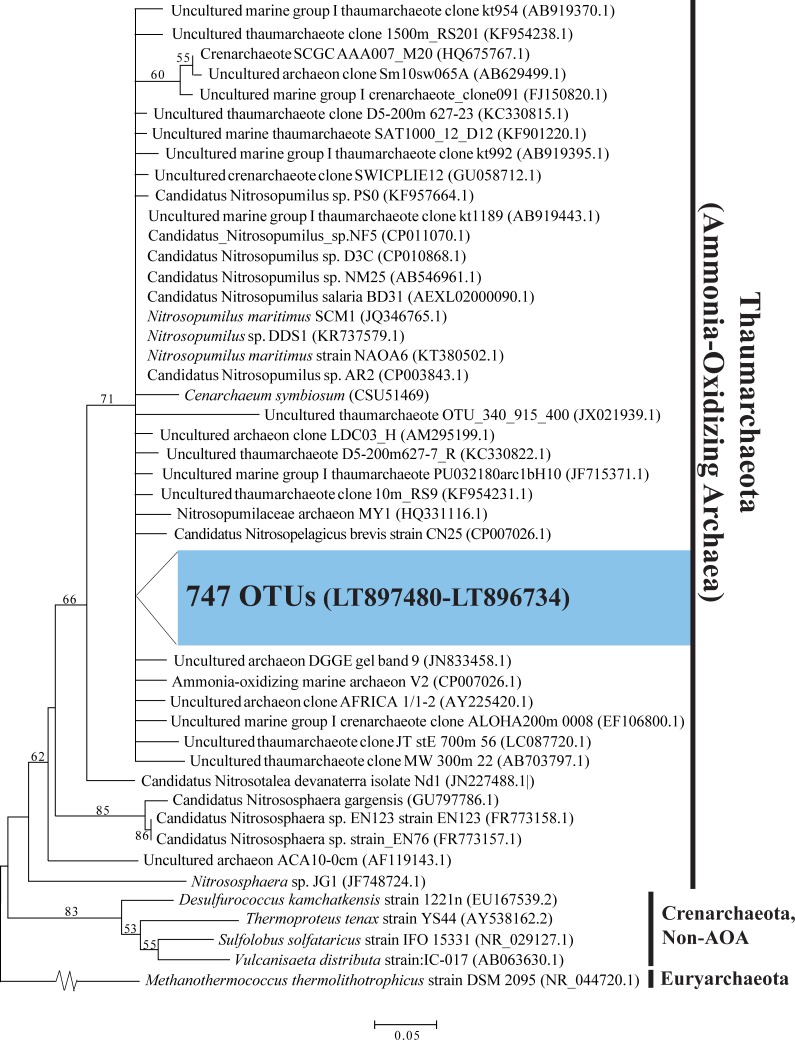
Phylogenetic relationships among ammonia-oxidizing archaea (AOA) of the Thaumarchaeota. **The tree is based on maximum likelihood (ML) analysis of 747 OTUs (∼253 bp) detected in this study (in blue), compared to close relatives.** Accession numbers are indicated in parentheses. Bootstrap values from 1000 replicates are indicated at the nodes of branches (if > 50). The scale bar represents the number of substitutions per site.

Of the total OTUs classified across all samples using the RDP classifier in QIIME, 747 were affiliated with AOA, 2 were affiliated with AOB, 295 were affiliated with NOB, and 11 were affiliated with MOB. The taxonomic assignment of these OTUs was further verified via BLASTN (against NCBI) and phylogenetic analysis. All 747 AOA OTUs had top BLASTN matches (88%–100% identity) to AOA of the Thaumarchaeota (S1 Table), with 745 of these having top matches to strains of the open ocean archaeon *Candidatus* Nitrosopelagicus brevis or to *Nitrosopumilus* spp. of the family Nitrosopumilaceae. The majority (57.8%) clustered into OTUs most similar (94%−100%) to *Ca*. N. brevis CN25 (S1 Table), a pelagic AOA member adapted to the oligotrophic surface ocean [61]. The remaining 2 AOA OTUs matched *Nitrososphaera* sp. of the family Nitrososphaeraceae, which thus far primarily includes representatives from soils (S1 Table) [62]. Phylogenetic analysis supported the AOA classification; all AOA OTUs clustered in a broad clade with diverse Thaumarchaeota (71% bootstrap support). Within this clade, however, OTU placement was not well resolved, likely due to the low information content of the short 16S rRNA gene sequences (Fig 2).

**Fig 3:**
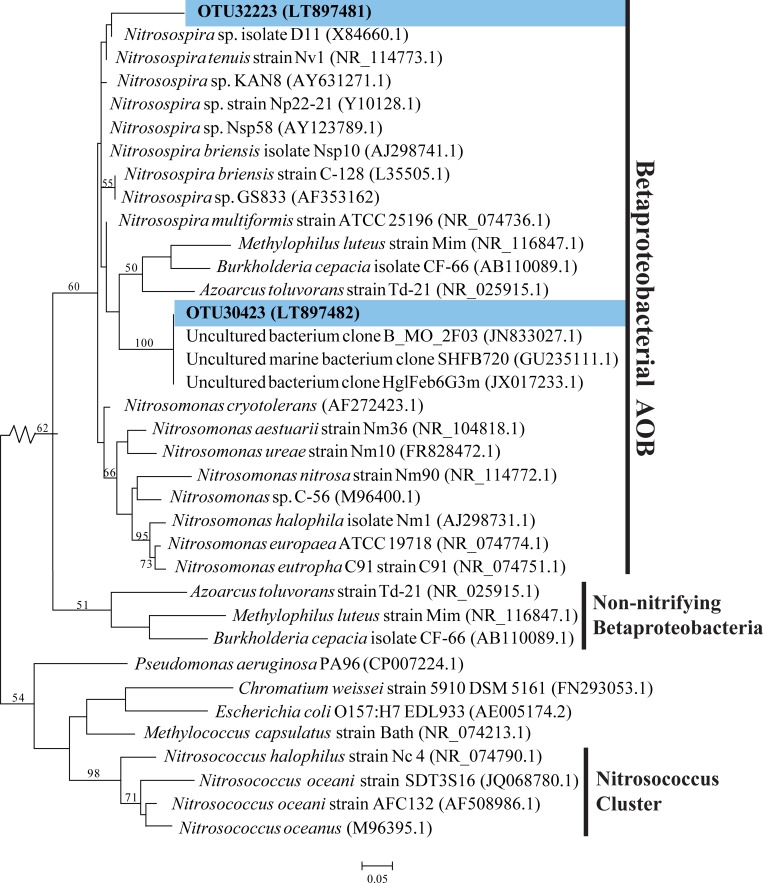
Phylogenetic relationships among ammonia-oxidizing bacteria (AOB). **The tree is based on maximum likelihood (ML) analysis of 2 OTUs (∼253 bp) detected in this study (in blue), compared to relatives from the Nitrosomonadaceae (Betaproteobacteria).** Accession numbers are indicated in parentheses. Bootstrap values from 1000 replicates are indicated at the nodes of branches (if > 50). The scale bar represents the number of substitutions per site.

Consistent with RDP classification, the 2 AOB OTUs matched AOB of the Nitrosomodaceae, sharing 89%–94% identity to *Nitrosospira briensis* C-128 from soil (S2 Table) [63]. These 2 OTUs grouped within a diverse but not well-supported (60% bootstrap support) clade of betaproteobacterial AOB, but represented two distinct lineages within this clade (Fig 3).

**Fig 4:**
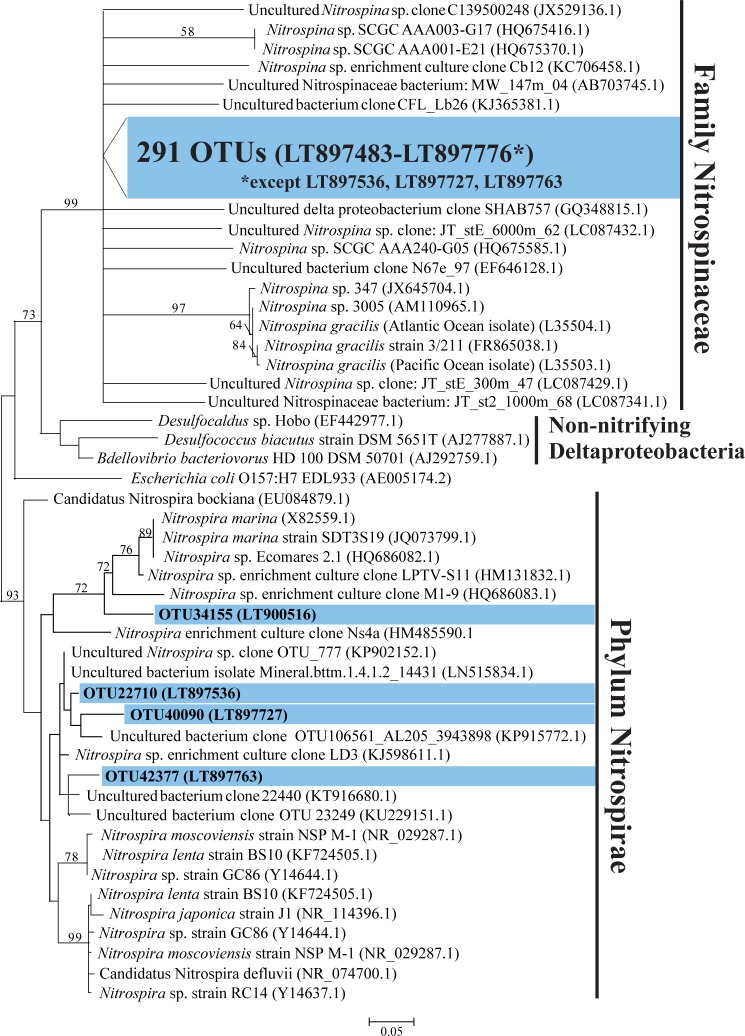
Phylogenetic relationship among nitrite-oxidizing bacteria (NOB). **The tree is based on maximum likelihood (ML) analysis of 295 OTUs (∼253 bp) detected in this study (in blue), compared to relatives from the phyla Nitrospirae and Nitrospinae.** Accession numbers are indicated in parentheses. Bootstrap values from 1000 replicates are indicated at the nodes of branches (if > 50). The scale bar represents the number of substitutions per site.

Of the 295 NOB OTUs, 291 had top BLASTN matches (87%–100% identity) to *Nitrospina* spp. of the phylum Nitrospinae, with over 80% of these being most similar to *Nitrospina gracilis* strain 3/211 (NR_104821.1) or *Nitrospina gracilis* (Atlantic Ocean isolate) (L35504.1) (S3 Table). These OTUs clustered unambiguously (99% bootstrap support) within the Nitrospinaceae but could not be resolved further within this clade (Fig 4). The remaining 4 NOB OTUs matched the phylum Nitrospirae, sharing 94%–98% identity with *Nitrospira* sp. enrichment culture clone LD3 from forest soil and *Nitrospira* sp. enrichment culture clone LPTV-S11 from a marine sponge (S3 Table) [64], and represented multiple lineages within the Nitrospirae clade (Fig 4).

Analysis of the 11 MOB OTUs identified 6 OTUs with top BLASTN matches (90%–97% identity) to the gammaproteobacterial family Methylococcaceae and 5 with matches (83%–84% identity) to the Verrucomicrobia family Methylacidiphilaceae (S4 Table). Phylogenetic analysis confirmed the family-level MOB classifications and also showed that these OTUs represent multiple lineages within each family (S5 Fig).

### Nitrifier abundance and distribution

Nitrifier relative abundances varied substantially with depth (S4 Fig). Notably, AOA sequences affiliated with Thaumarchaeota sequences increased from ~1% of total sequences at 10 m to 33% at 130 m, before declining slightly to 25% at 250 m (S4 Fig). In contrast, AOB were largely absent; the ratio of AOA to AOB sequences exceeded 90,000:1 at all depths where AOB were detected (only at 25 m, 100 m, and 130 m; S2 Table). Similarly, AOA sequences substantially outnumbered nitrite oxidizer sequences at all depths. The ratio of ammonia (AOA+AOB) to nitrite oxidizer sequences was 41:1 at 10 m, but declined with depth to under 27:1 at 250 m (Table 1). Along this depth gradient, sequences matching the NOB phylum Nitrospinae increased from 0% at 10 m to 0.9% at 250 m, representing > 99.9% of all NOB counts at all depths sampled, whereas those of the phylum Nitrospirae comprised < 0.1% (S4 Fig; S3 Table). NOB sequences matching *Nitrococcus*, *Nitrobacter*, or *Nitrotoga* were not detected. The percentage abundance of both the ammonia oxidizer community was weakly correlated with dissolved O_2_ concentration and temperature with the relationship being strongest for the NOB community (*r* = –0.81 for correlations with O_2_ and temperature, respectively; *p* < 0.1; S5 Table). MOB sequences were rare, constituting less than 0.0005% of all sequences at all depths (S4 Table).

**Table 1:** Relative abundances of sequences matching ammonia-oxidizing microbes (AOA + AOB) and nitrite-oxidizing bacteria (NOB), expressed as a proportion of total sequences and as a ratio of (AOA+AOB) to NOB counts.

	AOA + AOB	NOB	C. (AOA+NOB):NOB
10 m	0.010	2.42 × 10^−4^	41:1
25 m	0.251	0.006	40:1
100 m	0.245	0.006	39:1
130 m	0.309	0.009	34:1
250 m	0.249	0.009	27:1

**Fig 5:**
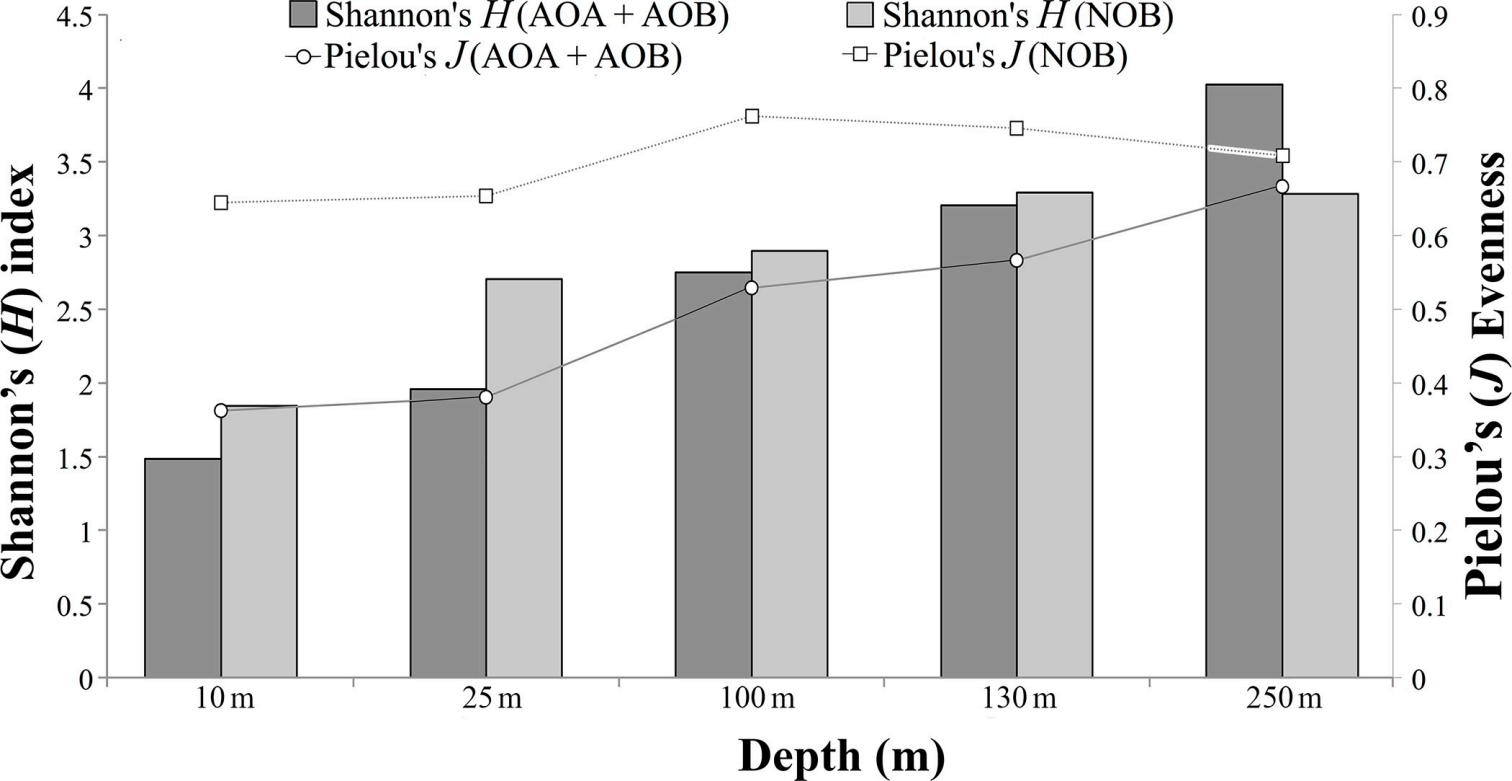
Diversity (*H*) and evenness (*J*) of ammonia-oxidizing (AOA + AOB) and nitrite-oxidizing (NOB) microbial communities. Depths of 10 and 25 m were in the oxic zone. Depths of 100, 130, and 250 m were in the hypoxic zone.

Nitrifier community structure varied with sample depth in our analyses of sequence reads from 5 depths at one site. The OTU richness (observed), diversity (*H*; Shannon-Weiner index), and evenness (*J*; Pielou’s index) of both the ammonia oxidizer (AOA+AOB) and NOB communities increased consistently with depth from the surface to the hypoxic zone (Fig 1, Fig 5, S4 Fig) and regression analysis indicated strong positive linear correlation between the *H* indices of AOA+AOB and NOB (*r* = 0.99) (S7 Fig). These trends differ from that of the total microbial community (all OTUs considered), in which richness was highest at the shallowest depths (10 m and 25 m; S4 Fig), and were most dramatic in the ammonia oxidizer community. Notably, the observed number of ammonia oxidizer OTUs (the overwhelming majority of which were AOA; see above) more than doubled from 25 m to 250 m (S6A Fig; evaluated at a common sequence depth of 150,000) and evenness increased by roughly 50% with depth (Fig 5), indicating a shift to a more equal representation of community members (fewer dominant taxa) from the surface to the hypoxic zone.

Most nitrifier OTUs were not detected broadly across all depths. Whereas 65% of ammonia oxidizer OTUs and 55% of NOB OTUs were detected at more than one depth, only 5% of ammonia oxidizer OTUs and 4% of NOB OTUs were detected at all 5 depths (S1 and S3 Tables). A subset of AOA OTUs {n = 22; marked with an asterisk (*) in S1 Table} was detected in at least 4 depths but was substantially enriched at the shallowest depths, collectively representing 84% and 94% of ammonia oxidizer reads at 10 m and 25 m, respectively, but only 21%, 2% and < 1% at 100 m, 130 m, and 250 m, respectively. Furthermore, hierarchical cluster analysis identified 55 AOA and 52 NOB (all placed within the genus *Nitrospina*) OTUs that were placed in 12 groups based on co-occurrence patterns (S6 Table).

## Discussion

Our data from an offshore site in the Benguela Upwelling system identified a prominent nitrifier community whose taxonomic composition varied substantially with depth, and in comparison to prior results from closer to the coast [7]. Consistent with studies showing AOA dominance over AOB in marine systems [1,18,38,65–70], the ammonia oxidizer community at station 116 was composed almost exclusively of Thaumarchaeota (AOA). AOA abundance increased from ~1% at oxic depths to up to 33% in the hypoxic zone, suggesting ammonia as a substantial energy source. In contrast, the NOB community, composed primarily of *Nitrospina*, never accounted for more than 1% of sequences. However, like the AOA, NOB abundance increased with depth. At depths of peak AOA and NOB abundance, O_2_ concentrations were low (~20–70 μM) compared to the surface but still considerably higher than levels known to inhibit anaerobic metabolisms such as denitrification and anammox (hundreds of nM to low μM) [e.g., 12,17,79]. Rather, minimum O_2_ levels at stations 116 and 117 were within the range (tens of μM) known to support high rates of both ammonia and nitrite oxidation within or at the periphery of low-oxygen zones where both AOA and NOB have been found at high abundance [e.g., 7,12–14,36,71,72].

Given these conditions, we hypothesize that AOA activity was the main local biological source of both N_2_O and NO_2_^−^ in the hypoxic layer, and that NOB of the *Nitrospina* were the main biological NO_2_^–^ sink. Indeed, the proportional increase in AOA with depth coincided with an increase in N_2_O concentration to > 40 nM (Fig 1B), a level consistent with that reported previously for the area [30,73]. In contrast, NO_2_^–^, as measured at a nearby station 117 (~10 km away) with O_2_ conditions similar to those at station 116 (Fig 1), reached a maximum only in the oxic photic zone (~25 m– 30 m) and a deeper secondary NO_2_^–^ maximum was not observed. At station 116, AOA abundances were low in the photic depths, potentially due to competition with phytoplankton [74]. Assuming similar NO_2_^–^ conditions existed at stations 116 and 117, then NO_2_^–^ accumulation at upper depths was likely driven by processes other than AOA activity, potentially incomplete assimilatory NO_3_^−^ reduction by phytoplankton [75]. Indeed, chloroplast genes represented 8–10% of 16S rRNA sequences at 10 and 25 m, coincident with high fluorescence values at these depths (S2 Fig). Given the concurrent increase in both AOA and NOB with depth, we propose that NO_2_^–^ production by AOA became increasingly coupled to NO_2_^–^ consumption by NOB beneath the primary NO_2_^–^ maximum and into the hypoxic zone. Indeed, at station 116, the AOA:NOB ratio was highest in oxic depths (41:1) and lowest in the hypoxic zone (27:1). The lowest value was similar to that reported in a prior study (20:1) to suggest AOA:NOB coupling in the northern Gulf of Mexico [38]. Here, and in the prior study, an apparent coupling occurred even though NOB were rare compared to AOA. Indeed, the genomes of most *Nitrospina* sp. contain two rRNA operons [37]; thus, the true relative abundance of NOB may be lower than what we report. It is also likely that the bulk of ammonia oxidation proceeded through AOA and not through MOB, which can also oxidize NH_3_ and thereby compete with ammonia oxidizers [76–78], but were barely detected in our data. A coupling between NO_2_^–^ production (by AOA) and consumption would therefore suggest either disproportionately high oxidation rates by the minority NOB community, or alternative NO_2_^–^ sinks.

These results provide a contrast to those of Füssel et al. (2012), which focused on sites closer to the coast on the Namibian Shelf (depths: 100 m– 130 m), where NO_2_^–^ maxima (> 4 μM) coincided with O_2_ levels (< 4 μM) considerably lower than those at our site. At such sites, NO_2_^−^ accumulation is likely driven primarily by NO_3_^−^ reduction, although low O_2_-adapted AOA may also contribute to NO_2_^−^ production [7,14,26]. Similarly, NO_2_^–^ consumption by low O_2_-adapted NOB may occur at such sites. Füssel et al. (2012) detected both *Nitrospina* and *Nitrococcus* NOB at up to ~5% (each) of the community at NO_2_^–^-enriched depths and measured NO_2_^–^ oxidation at rates exceeding those of NH_3_ oxidation [7]. By comparison, *Nitrospina* and *Nitrospira* represented > 99% and < 0.01% of NOB counts at all depths, respectively, and we did not detect *Nitrococcus*. Rather, *Nitrospira* sequences, while present in our data (at low abundance) but not detected in Füssel et al. (2012), raising the hypothesis that *Nitrococcus* better tolerates low O_2_ compared to *Nitrospira*. This is consistent with evidence suggesting diverse metabolic capabilities in *Nitrococcus*, including nitrate reduction and sulfide oxidation [8]. Füssel et al. (2012) also measured anammox rates comparable to, or exceeding those of NO_2_^–^ oxidation at O_2_-depleted depths [7], consistent with prior work indicating anammox as a major NO_2_^–^ sink in O_2_-deficient waters in this region [e.g., 29,80] and therefore as a potential competitor with NOB. In contrast, we did not detect anammox bacteria at station 116, where O_2_ levels exceeded the upper limit for anammox [12,17,79]. Together, these results suggest that in the relatively O_2_-enriched water over the continental slope, a proportionally less abundant (but potentially highly active) NOB community may balance NO_2_^–^ production by AOA. In contrast, in more O_2_-depleted waters closer to shore, the NOB community differs in composition and is at higher proportional abundance (versus overall microbial abundance) {~10% in Füssel et al. (2012) compared to 1% in our study}, but still insufficient to balance the combined NO_2_^–^ production by AOA and nitrate reduction, even with concurrent NO_2_^–^ consumption by anammox.

These results highlight variability in the nitrifier community with proximity to shore in the Benguela system. Variability is also evident over depth. Here, both the AOA and NOB communities exhibited lower evenness in oxic compared to hypoxic depths. This trend suggests that nitrification may proceed disproportionately through a minority of nitrifier taxa at oxic depths, but more uniformly through diverse nitrifier taxa at hypoxic depths. Notably, the subset of AOA OTUs (n = 22; S1 Table) that we identified as enriched (> 84% of sequences) in the upper depths may be the main contributors to ammonia oxidation in this zone. While our analysis suggests that many nitrifier OTUs occur across multiple depths, certain OTUs consistently co-occur with others (S6 Table), further suggesting non-random community structuring with depth. As O_2_ levels fell, nitrifier richness and diversity (Shannon *H*), particularly in the AOA community, increased significantly (> 2-fold), to over 500 OTUs at hypoxic depths (S6A and S6B Fig); indeed, AOA diversity may be underestimated, as the PCR primers used in our study may exclude certain archaeal groups [81]. A similar inverse relationship between diversity and O_2_ was observed at the periphery of Eastern Pacific OMZs, where total (nitrifier + non-nitrifier) microbial richness increased with depth and peaked at O_2_ levels of ~10–15 μM [82]. However, the relationship between microbial diversity and O_2_ is complex. At low to sub- μM O_2_ levels, the relationship becomes positive, with diversity decreasing as O_2_ decreases [82,83]. Further, the relationship differs among microbes; here, the negative relationship noted above for nitrifiers differs from the weak positive relationship observed for the entire community (S4 Fig). Together, these results suggest restructuring of nitrifier communities with depth, and potentially O_2_ availability.

Despite their small volume in the oceans, oxygen deficient zones play a critical role in the oceanic N budget, with reductive microbial metabolisms at the core of these zones contributing 30%–50% of oceanic N loss [84,85]. These zones are expanding [31,86], making it critical to understand the microbial consequences of differing oxygen regimes. Oxygen conditions at our study site were intermediate between those of the oxic open ocean and the intensively O_2_-depleted N loss zones closer to shore. Under these conditions, AOA appear to thrive and outnumber NOB by at least 25-fold. Yet NO_2_^–^ does not accumulate, suggesting a tight coupling between the two nitrifying communities. It has been suggested that ammonia-oxidizing microbes and NOB may engage in physically close, syntrophic associations [87], with NOB presumably benefiting from NO_2_^–^ supplied by AOA, and AOA (and/or AOB) benefiting from NOB activity, which prevents NO_2_^–^ from accumulating to toxic concentrations [87–90]. Indeed, the co-occurrence between specific AOA and NOB OTUs (S6 Table) suggests potentially interacting taxa. Describing nitrifier diversity across geochemically variable sites remains a research priority [e.g., 7,9,10,11,14,34,36,67–69]. Alongside results from prior studies, our data inform a broad effort to predict how changing O_2_ levels affect the structure and activity of the ocean N cycle.

## Supporting information

S1 Fig Complete dissolved O_2_ profile for station 116, from surface to 860 m. Seabed depth: 862 m– 868 m.
(PDF)

S2 Fig FIECO-AFL for station 116, from surface to 300 m. (PDF)

S3 Fig Microbial taxon richness at station 116, assessed by fifteen rarefaction curves (triplicate PCR amplifications for 5 different depths) for microbial OTUs in this study. (PDF)

S4 Fig Relative sequence abundances of archaeal and bacterial phyla or class based on the 833,468 unique OTU reads (out of 5,141,055 total reads) obtained from separate reactions in triplicate for each depth (10 m, 25 m, 100 m, 130 m, and 250 m) in the Namibian upwelling seawater at Station 116. (PDF)

S5 Fig Phylogenetic tree based on maximum likelihood (ML) analysis of 11 OTUs (~253 bp) of MOB and putative MOB detected in this study (in bold) in comparison with their close relatives and representatives from the families methyacidiphilaceae (phylum Verrucomicrobia) and Methylococcaceae (class gammaproteobacteria). (PDF)

S6 Fig Taxon richness from 10 m, 25 m, 100 m, 130 m, and 250 m depths, assessed by rarefaction curves for (A) ammonia oxidizer OTUs (AOA+AOB), and (B) nitrite oxidizer (NOB) OTUs in this study.
(PDF)

S7 Fig Shannon diversity (*H*) of the AOA and AOB community compared to that of the NOB community. (PDF)

S1 Table Top named or cultured representative(s) based on BLASTN searches and read counts matching the 747 OTUs related to the ammonia-oxidizing archaea (AOA). (PDF)

S2 Table Top named or cultured representative(s) based on BLASTN searches and read counts matching the 2 OTUs related to the bacterial ammonia-oxidizing family Nitrosomodaceae. (PDF)

S3 Table Top named or cultured representative(s) based on BLASTN searches and read counts matching the 295 OTUs related to the bacterial nitrite-oxidizing family Nitrospinaceae and 4 OTUs related to the bacterial nitrite-oxidizing phylum Nitrospirae. (PDF)

S4 Table Top named or cultured representative(s) based on BLASTN searches and read counts matching the 6 OTUs related to MOB of the gammaproteobacterial family Methylococcaceae, and 5 OTUs related to MOB of the Verrucomicrobial family Methylacidiphilaceae. (PDF)

S5 Table Adjusted R (correlation coefficient) and *P* values in comparisons between nitrifier abundances (percentage of total sequences) versus temperature, dissolved O_2_ concentration, and N_2_O concentration. (PDF)

S6 Table Co-occurrence patterns of 55 AOA and 52 NOB OTUs. (PDF)
