# Supplementary material for: Diversity and relative abundance of ammonia- and nitrite-oxidizing microorganisms in the offshore Namibian hypoxic zone
Source: PLoS One. 2019 May 21;14(5):e0217136. doi: 10.1371/journal.pone.0217136 (PMC6529010; doi:10.1371/journal.pone.0217136)

S1 Fig. Complete dissolved O_2 profile for station 116, from surface to 860 m. Seabed depth: 862–868 m. The most O_2-depleted hypoxic core of Benguela islocated from 170–340 m depths, where O_2 concentration ranged from 20 to 25 μM.

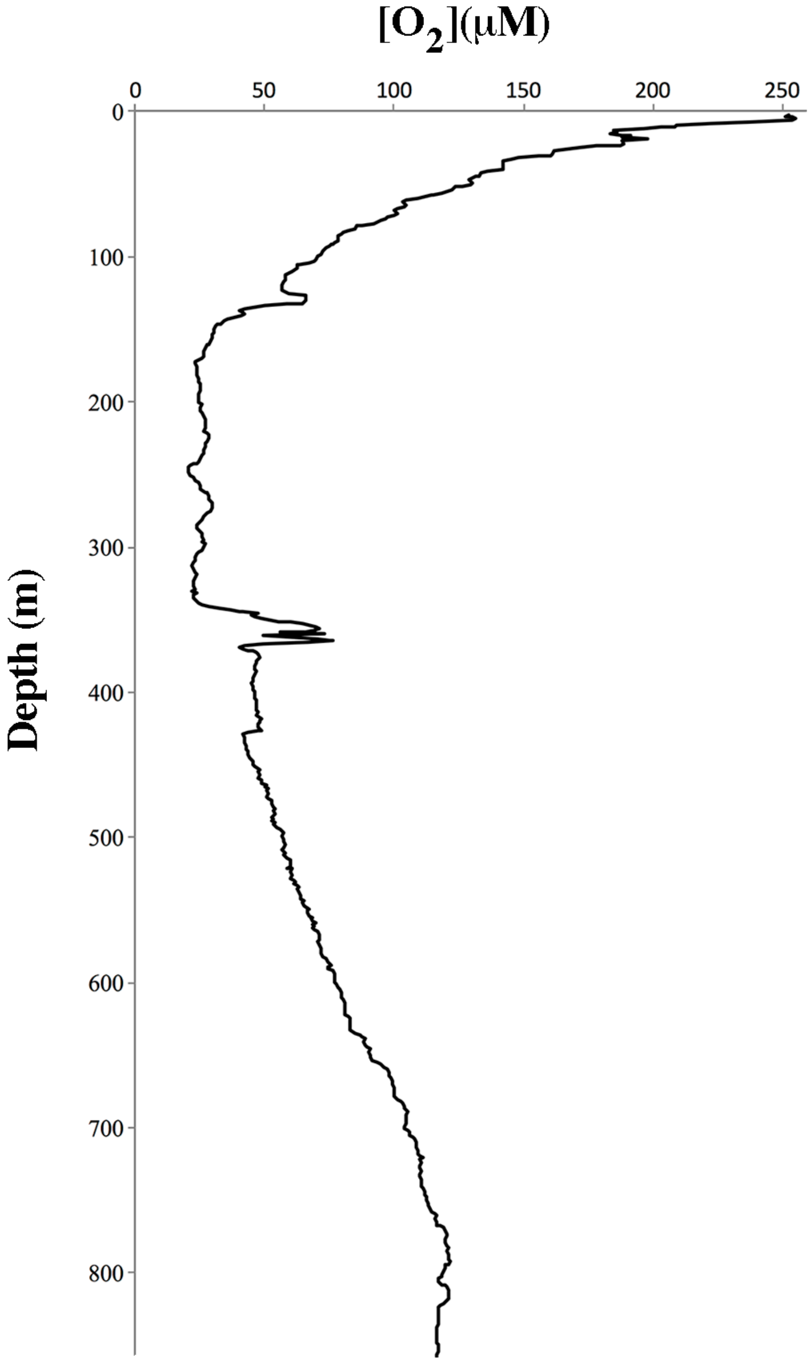

Supplement: S1 Fig — Seabed depth: 862 m– 868 m. (PDF) [file pone.0217136.s001.pdf]
