# Supplementary material for: Diversity and relative abundance of ammonia- and nitrite-oxidizing microorganisms in the offshore Namibian hypoxic zone
Source: PLoS One. 2019 May 21;14(5):e0217136. doi: 10.1371/journal.pone.0217136 (PMC6529010; doi:10.1371/journal.pone.0217136)

S2 Fig. FIECO-AFL for station 116, from surface to 300 m. The FIECO-AFL is an indirect measure of (chlorophyll) pigment concentration at different depths and indicates the abundance of photosynthetic microorganisms in the water column.

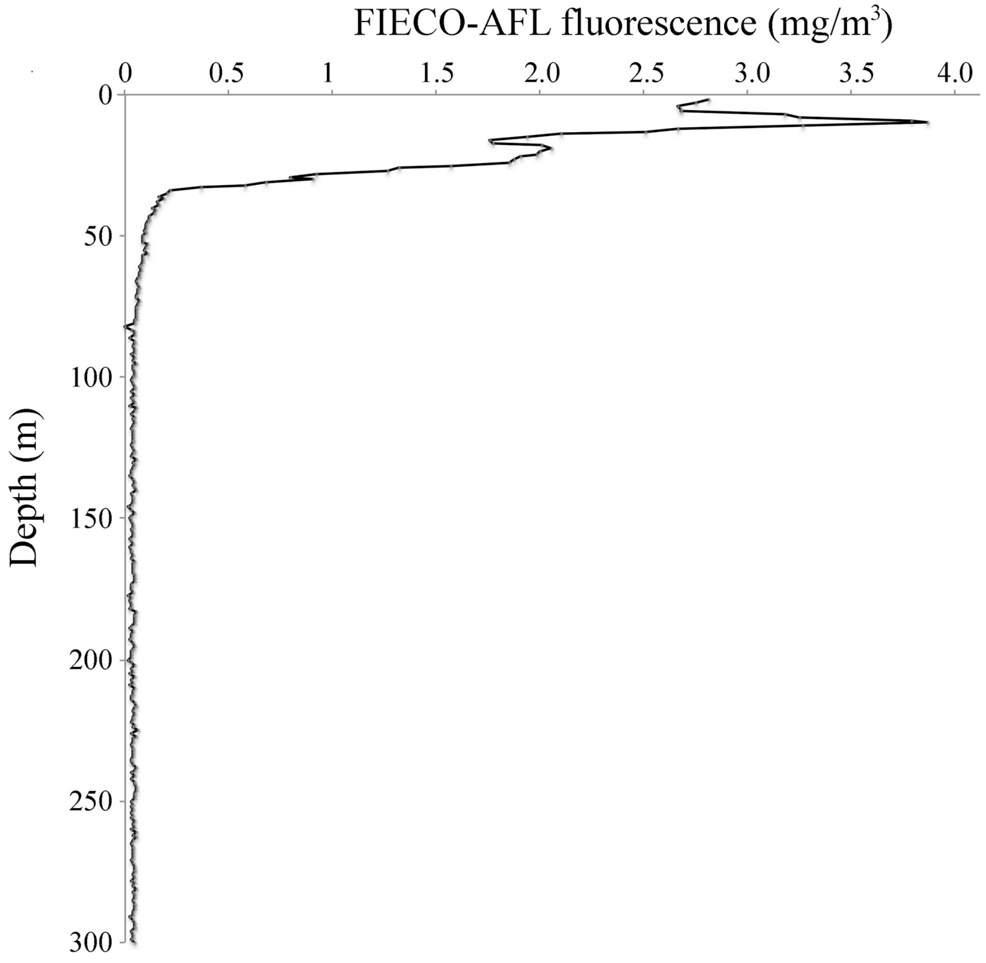

Supplement: S2 Fig — (PDF) [file pone.0217136.s002.pdf]
