# Supplementary material for: Diversity and relative abundance of ammonia- and nitrite-oxidizing microorganisms in the offshore Namibian hypoxic zone
Source: PLoS One. 2019 May 21;14(5):e0217136. doi: 10.1371/journal.pone.0217136 (PMC6529010; doi:10.1371/journal.pone.0217136)

S3 Fig. Microbial taxon richness at station 116, assessed by fifteen rarefaction curves (triplicate PCR amplifications for 5 different depths) for microbial OTUs in this study. For each depth, three separate PCR amplifications, using different barcoded primers were generated for a total of 15 different reactions. Curves are labeled by depth, followed by "-1," "-2," and "-3," for replicates, with the arrows pointing at the terminal of each curve. The rarefaction curves (from Chao1) show the relationship between increasing the number of random samplings and new, unique microbial OTUs, assesses species richness from the results of sampling.

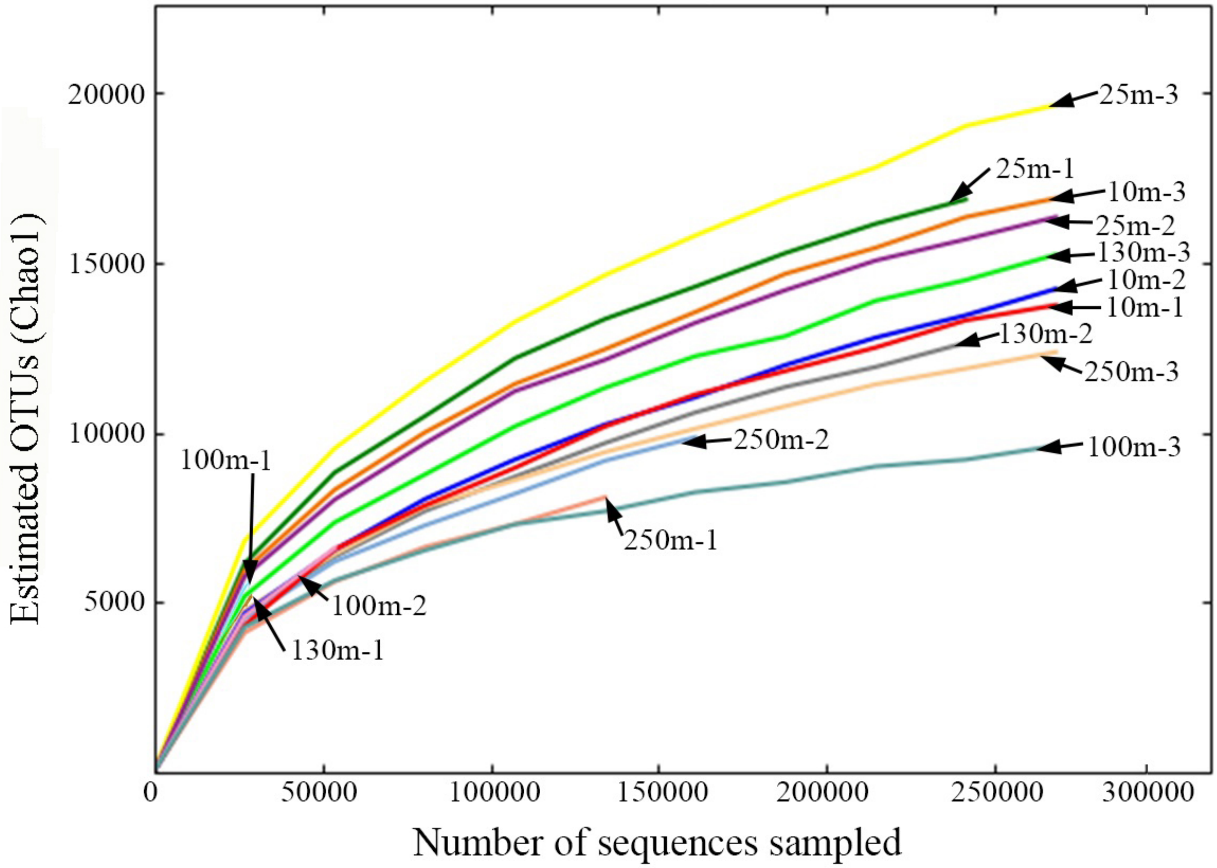

Supplement: S3 Fig — (PDF) [file pone.0217136.s003.pdf]
