# Supplementary material for: Diversity and relative abundance of ammonia- and nitrite-oxidizing microorganisms in the offshore Namibian hypoxic zone
Source: PLoS One. 2019 May 21;14(5):e0217136. doi: 10.1371/journal.pone.0217136 (PMC6529010; doi:10.1371/journal.pone.0217136)

S4 Fig. Relative sequence abundances of Archaeal and Bacterial phyla or class based on the 833,468 unique OTU reads (out of 5,141,055 total reads) obtained from separate reactions in triplicate for each depth (10 m, 25 m, 100 m, 130 m, and 250 m) in the Namibian Upwelling seawater at Station 116. The Thaumarchaeota, which consist of all AOA, constitutes at least 25% of the microbiota at depth 100 m and below.

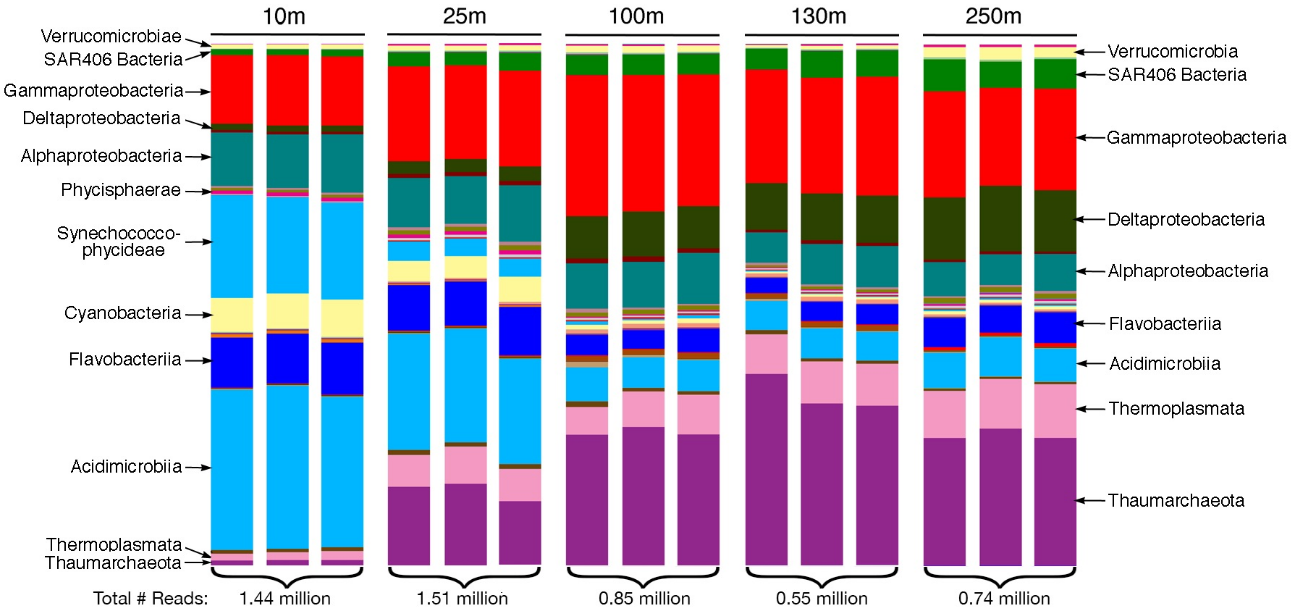

Supplement: S4 Fig — (PDF) [file pone.0217136.s004.pdf]
