# Supplementary material for: Diversity and relative abundance of ammonia- and nitrite-oxidizing microorganisms in the offshore Namibian hypoxic zone
Source: PLoS One. 2019 May 21;14(5):e0217136. doi: 10.1371/journal.pone.0217136 (PMC6529010; doi:10.1371/journal.pone.0217136)

S5 Fig. Phylogenetic tree based on maximum likelihood (ML) analysis of 11 OTUs (~253 bp) of MOB and putative MOB detected in this study (in bold) in comparison with their close relatives and representatives from the families Methyacidiphilaceae (phylum Verrucomicrobia) and Methylococcaceae (class Gammaproteobacteria). The accession numbers are indicated in the parentheses. Bootstrap values from 1000 replicates are indicated at the nodes of branches (if > 50). The scale bar represents the number of substitutions per site.

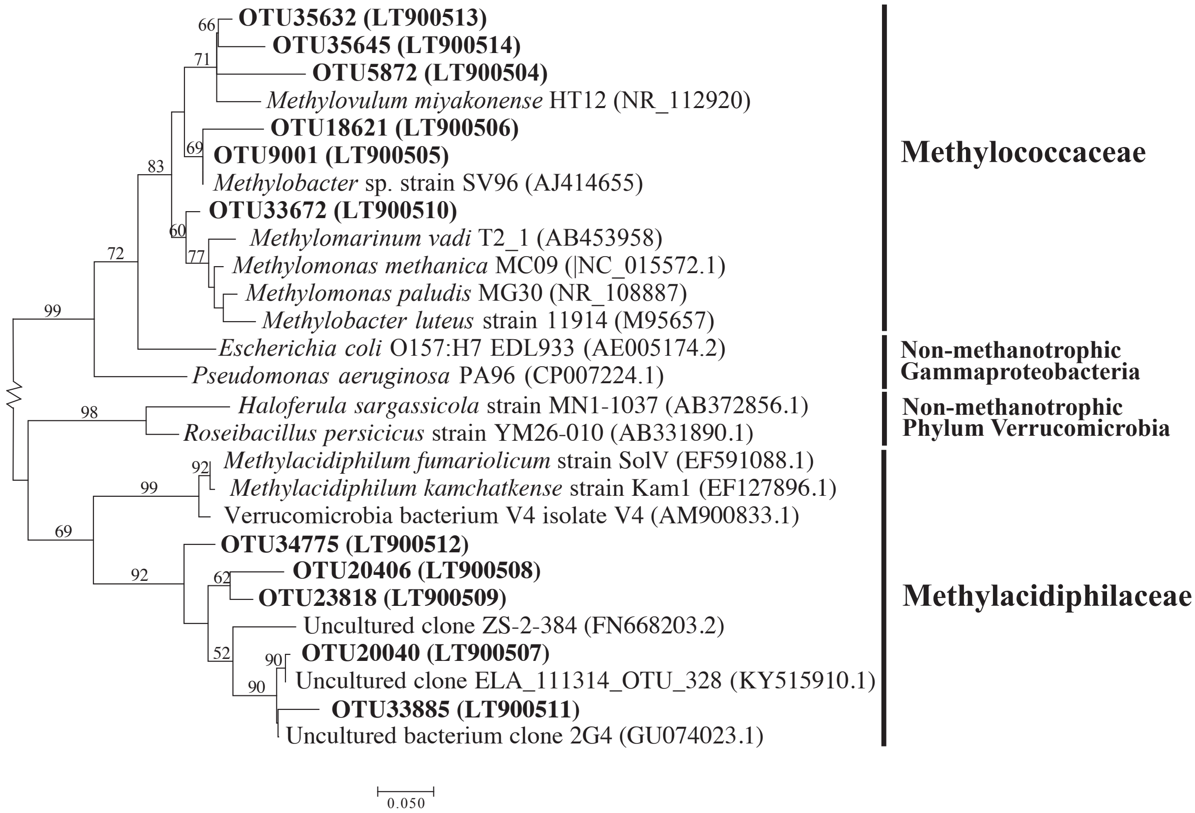

Supplement: S5 Fig — (PDF) [file pone.0217136.s005.pdf]
