# Supplementary material for: Diversity and relative abundance of ammonia- and nitrite-oxidizing microorganisms in the offshore Namibian hypoxic zone
Source: PLoS One. 2019 May 21;14(5):e0217136. doi: 10.1371/journal.pone.0217136 (PMC6529010; doi:10.1371/journal.pone.0217136)
Supplement: S6 Fig — Taxon richness from 10 m, 25 m, 100 m, 130 m, and 250 m depths, assessed by rarefaction curves for (A) ammonia oxidizer OTUs (AOA+AOB), and (B) nitrite oxidizer (NOB) OTUs in this study. (PDF) [file pone.0217136.s006.pdf]

S6 Fig. Taxon richness from 10 m, 25 m, 100 m, 130 m, and 250 m depths, assessed by rarefaction curves for (A) ammonia oxidizer OTUs (AOA+AOB), and (B) nitrite oxidizer (NOB) OTUs in this study. The rarefaction curves show the relationship between increasing the number of random samplings and new, unique nitrifier OTUs in order to assess species richness from the results of sampling.

(A)

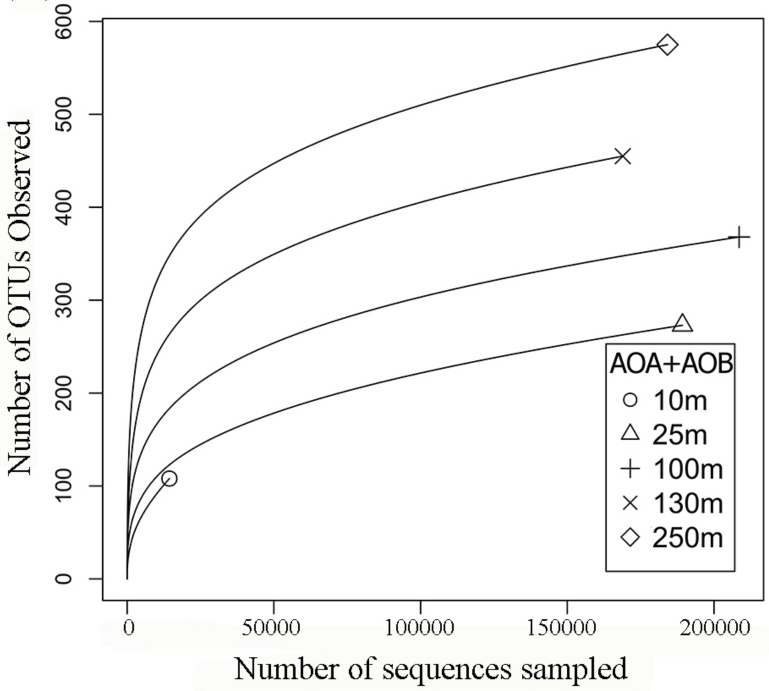

(B)

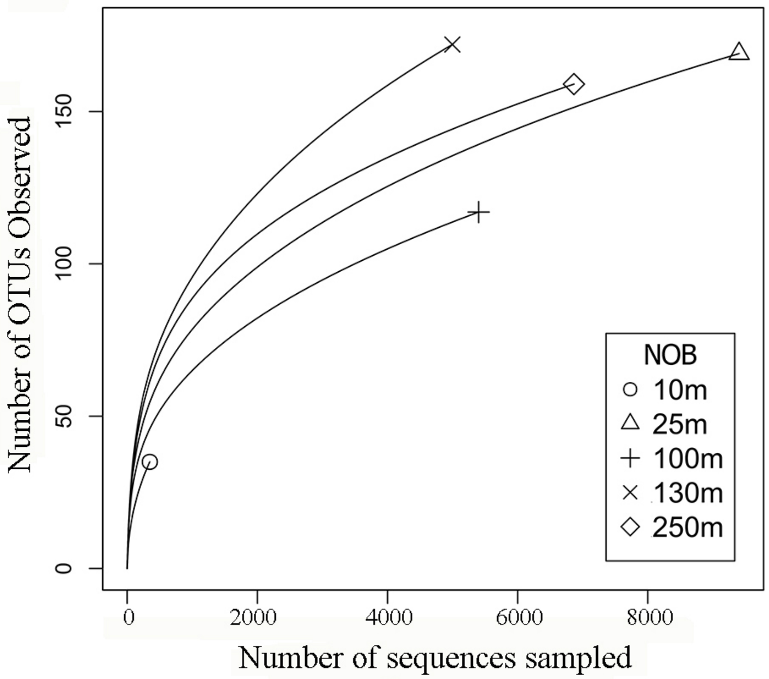

\
