# Supplementary figures and images for: Diversity and relative abundance of ammonia- and nitrite-oxidizing microorganisms in the offshore Namibian hypoxic zone
Source: PLoS One. 2019 May 21;14(5):e0217136. doi: 10.1371/journal.pone.0217136 (PMC6529010; doi:10.1371/journal.pone.0217136)

**S7 Fig. Shannon diversity ( $H$ ) of the AOA and AOB community compared to that of the NOB community.  $r = 0.99$  ( $p < 0.01$ ).**

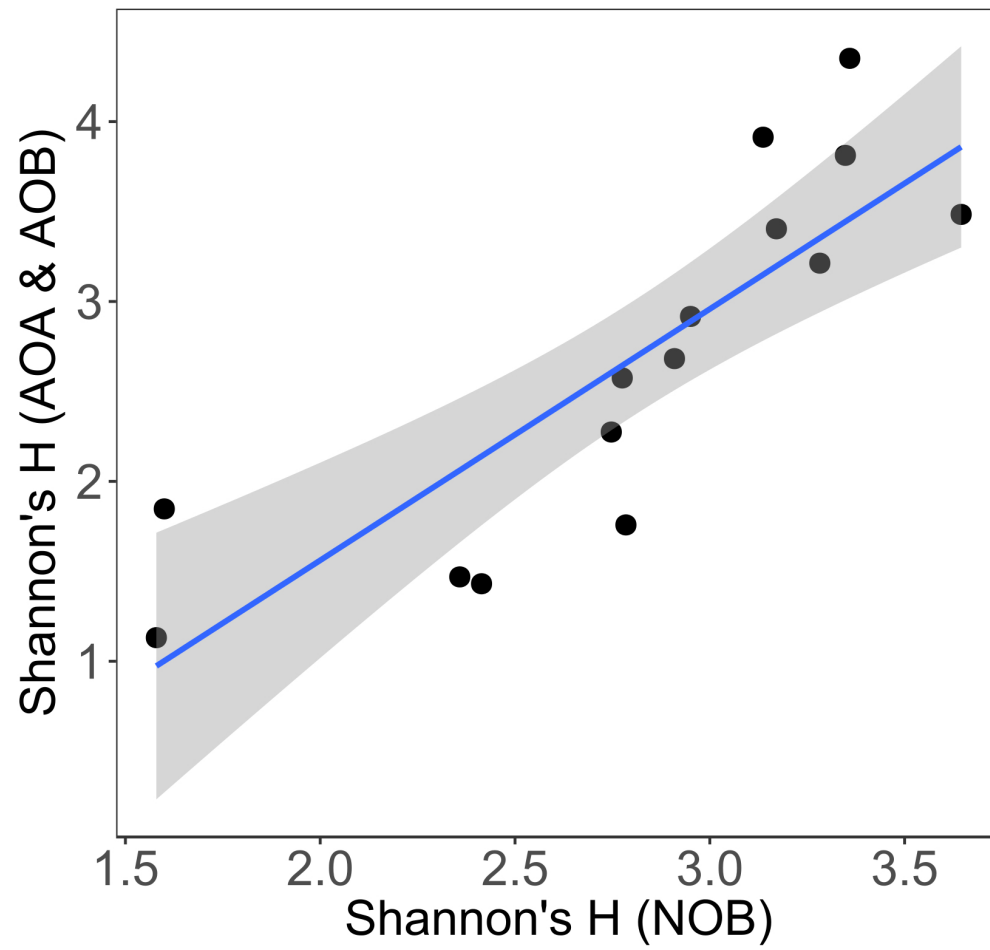

Supplement: S7 Fig — (PDF) [file pone.0217136.s007.pdf]
