# Supplementary material for: Diversity and relative abundance of ammonia- and nitrite-oxidizing microorganisms in the offshore Namibian hypoxic zone
Source: PLoS One. 2019 May 21;14(5):e0217136. doi: 10.1371/journal.pone.0217136 (PMC6529010; doi:10.1371/journal.pone.0217136)
Supplement: S1 Table — (PDF) [file pone.0217136.s008.pdf]

S1 Table. Top named or cultured representative(s) based on BLASTN searches and read counts matching the 747 OTUs related to the ammonia-oxidizing Archaea (AOA). The 22 OTUs enriched at 10 m and 25 m but relatively less abundant at deeper depths are indicated with an asterisk (*) after the OTU number (see main text).

OTU	GenBank Accession no.	Top named or cultured representative(s)	%ID to match	10m	25m	100m	130m	250m
**306**	LT896734	*Candidatus* Nitrosopelagicus strain CN25 (CP007026.1)	98%	187	439	33	247	56
**820**	LT896735	*Nitrosopumilus maritimus* strain SCM1 (NR_102913.1), *Candidatus* Nitrosopumilus koreensis strain AR1 (NR_102904.1), *Candidatus* Nitrosopumilus sp. AR2 (CP003843.1)	97%	3	37	2	41	28
**1972**	LT896736	*Nitrosopumilus maritimus* strain SCM1 (NR_102913.1), *Candidatus* Nitrosopumilus koreensis strain AR1 (NR_102904.1), *Candidatus* Nitrosopumilus sp. AR2 (CP003843.1), *Candidatus* Nitrosopumilus sp. NF5 (CP011070.1), *Candidatus* Nitrosopumilus sp. D3C (CP010868.1)	99%	23	577	21	76	19
**2000**	LT896737	*Candidatus* Nitrosopelagicus brevis strain CN25 (CP007026.1)	99%	158	866	69	18	1
**2383**	LT896738	*Nitrosopumilus maritimus* strain SCM1 (NR_102913.1), *Candidatus* Nitrosopumilus koreensis strain AR1 (NR_102904.1), *Candidatus* Nitrosopumilus sp. AR2 (CP003843.1)	90%	7	25	0	1	2
**2464**	LT896739	*Candidatus* Nitrosopelagicus brevis strain CN25 (CP007026.1)	98%	27	6	0	1	2
**2960**	LT896740	*Candidatus* Nitrosopelagicus brevis strain CN25 (CP007026.1)	96%	0	220	28	388	27
**3097**	LT896741	*Candidatus* Nitrosopelagicus brevis strain CN25 (CP007026.1)	97%	0	4	0	4	1
**3206**	LT896742	*Candidatus* Nitrosopelagicus brevis strain CN25 (CP007026.1)	100%	263	673	439	347	64
**3225**	LT896743	*Nitrosopumilus maritimus* strain SCM1 (NR_102913.1), *Candidatus* Nitrosopumilus koreensis strain AR1 (NR_102904.1), *Candidatus* Nitrosopumilus sp. AR2 (CP003843.1),	98%	24	55	1	35	6
**3272**	LT896744	*Candidatus* Nitrosopelagicus brevis strain CN25 (CP007026.1)	95%	1	1051	100	1375	1407
**3285***	LT896745	*Nitrosopumilus maritimus* strain SCM1 (NR_102913.1), *Candidatus* Nitrosopumilus koreensis strain AR1 (NR_102904.1), *Candidatus* Nitrosopumilus sp. AR2 (CP003843.1)	100%	2942	3153	17	143	85
**3306**	LT896746	*Candidatus* Nitrosopelagicus brevis strain CN25 (CP007026.1)	95%	0	36	6	67	23

**3343**	LT896747	*Nitrosopumilus maritimus* strain SCM1 (NR_102913.1), *Candidatus* Nitrosopumilus koreensis strain AR1 (NR_102904.1), *Candidatus* Nitrosopumilus sp. AR2 (CP003843.1)	98%	11	38	0	3	1
**3353**	LT896748	*Candidatus* Nitrosopumilus sp. NF5 (CP011070.1), *Candidatus* Nitrosopumilus sp. D3C (CP010868.1), *Candidatus* Nitrosopelagicus brevis strain CN25 (CP007026.1), *Nitrosopumilus maritimus* strain SCM1 (NR_102913.1), *Candidatus* Nitrosopumilus koreensis strain AR1 (NR_102904.1), *Candidatus* Nitrosopumilus sp. AR2 (CP003843.1)	96%	37	39	5	46	359
**3359**	LT896749	*Candidatus* Nitrosopelagicus brevis strain CN25 (CP007026.1)	96%	5	48	49	106	12
**3380**	LT896750	*Candidatus* Nitrosopelagicus brevis strain CN25 (CP007026.1)	98%	342	23	15	22	484
**3491**	LT896751	*Candidatus* Nitrosopelagicus brevis strain CN25 (CP007026.1)	96%	14	31	11	32	236
**3503**	LT896752	*Candidatus* Nitrosopelagicus brevis strain CN25 (CP007026.1)	94%	5	199	37	270	688
**3626**	LT896753	*Candidatus* Nitrosopumilus sp. NF5 (CP011070.1), *Candidatus* Nitrosopumilus sp. D3C, (CP010868.1)	96%	3	67	11	126	1448
**3801**	LT896754	*Candidatus* Nitrosopelagicus brevis strain CN25 (CP007026.1)	97%	25	48	5	48	982
**3810**	LT896755	*Candidatus* Nitrosopelagicus brevis strain CN25 (CP007026.1)	95%	22	369	299	453	260
**4113**	LT896756	*Candidatus* Nitrosopumilus sp. NF5e (CP011070.1), *Candidatus* Nitrosopumilus sp. D3C (CP010868.1)	89%	1	0	0	0	0
**5065**	LT896757	*Candidatus* Nitrosopumilus sp. NF5 (CP011070.1)	96%	5	0	0	0	3
**5077**	LT896758	*Candidatus* Nitrosopumilus sp. NF5 (CP011070.1), *Candidatus* Nitrosopumilus sp. D3C (CP010868.1), *Candidatus* Nitrosopelagicus brevis strain CN25 (CP007026.1), *Nitrosopumilus maritimus* strain SCM1 (NR_102913.1), *Candidatus* Nitrosopumilus koreensis strain AR1 (NR_102904.1), *Candidatus* Nitrosopumilus sp. AR2 (CP003843.1)	96%	9	59	2	1	3
**5612**	LT896759	*Candidatus* Nitrosopumilus sp. NF5 (CP011070.1), *Candidatus* Nitrosopumilus sp. D3C (CP010868.1), *Candidatus* Nitrosopelagicus brevis strain CN25 (CP007026.1), *Nitrosopumilus maritimus* strain SCM1 (NR_102913.1), *Candidatus* Nitrosopumilus koreensis strain AR1 (NR_102904.1), *Candidatus* Nitrosopumilus sp. AR2 (CP003843.1)	92%	2	4	0	0	0
**5820**	LT896760	*Candidatus* Nitrosopelagicus brevis strain CN25 (CP007026.1)	95%	1	0	0	0	0

**6126**	LT896761	*Nitrosopumilus maritimus* strain SCM1 (NR_102913.1), *Candidatus* Nitrosopumilus koreensis strain AR1 (NR_102904.1), *Candidatus* Nitrosopumilus sp. AR2 (CP003843.1)	99%	86	74	0	7	3
**6188**	LT896762	*Candidatus* Nitrosopumilus sp. NF5 (CP011070.1), *Candidatus* Nitrosopumilus sp. D3C (CP010868.1), *Candidatus* Nitrosopelagicus brevis strain CN25 (CP007026.1)	88%	1	0	0	0	0
**6312**	LT896763	*Candidatus* Nitrosopelagicus brevis strain CN25 (CP007026.1)	95%	11	20	19	7	4
**6364**	LT896764	*Candidatus* Nitrosopumilus sp. NF5 (CP011070.1)	96%	14	81	20	244	1794
**6810**	LT896765	*Nitrosopumilus maritimus* SCM1 strain (NR_102913.1)	89%	1	0	0	0	0
**6964**	LT896766	*Candidatus* Nitrosopumilus koreensis AR1 strain (NR_102904.1)	93%	1	2	0	1	0
**7168**	LT896767	*Candidatus* Nitrososphaera gargensis (NR_102916.1)	83%	1	7	1	1	2
**7402**	LT896768	*Nitrosopumilus maritimus* strain SCM1 (NR_102913.1), *Candidatus* Nitrosopumilus koreensis strain AR1 (NR_102904.1)	92%	1	0	0	1	0
**7569***	LT896769	*Nitrosopumilus maritimus* strain SCM1 (NR_102913.1), *Candidatus* Nitrosopumilus koreensis strain AR1 (NR_102904.1)	100%	1267	261	5	36	12
**7639**	LT896770	*Nitrosopumilus maritimus* strain SCM1 (NR_102913.1), *Candidatus* Nitrosopumilus koreensis strain AR1 (NR_102904.1)	87%	1	9	0	6	2
**7793**	LT896771	*Nitrosopumilus maritimus* strain SCM1 (NR_102913.1), *Candidatus* Nitrosopumilus koreensis strain AR1 (NR_102904.1)	94%	3	0	0	0	0
**8000**	LT896772	*Nitrosopumilus maritimus* strain SCM1 (NR_102913.1), *Candidatus* Nitrosopumilus koreensis strain AR1 (NR_102904.1)	96%	13	7	0	12	63
**8028**	LT896773	*Nitrosopumilus maritimus* strain SCM1 (NR_102913.1), *Candidatus* Nitrosopumilus koreensis strain AR1 (NR_102904.1)	95%	1	0	0	0	5
**8236**	LT896774	*Nitrosopumilus maritimus* strain SCM1 (NR_102913.1), *Candidatus* Nitrosopumilus koreensis strain AR1 (NR_102904.1)	93%	7	8	0	0	0
**8345**	LT896775	*Nitrosopumilus maritimus* strain SCM1 (NR_102913.1), *Candidatus* Nitrosopumilus koreensis strain AR1 (NR_102904.1)	88%	1	0	0	0	0

**8529**	LT896776	*Nitrosopumilus maritimus* strain SCM1 (NR_102913.1), *Candidatus* Nitrosopumilus koreensis strain AR1 (NR_102904.1)	95%	2	0	0	0	0
**8831**	LT896777	*Nitrosopumilus maritimus* strain SCM1 (NR_102913.1), *Candidatus* Nitrosopumilus koreensis strain AR1 (NR_102904.1)	92%	1	0	0	0	2
**9447**	LT896778	*Nitrosopumilus maritimus* strain SCM1 (NR_102913.1), *Candidatus* Nitrosopumilus koreensis strain AR1 (NR_102904.1)	95%	7	11	6	3	16
**9638**	LT896779	*Nitrosopumilus maritimus* strain SCM1 (NR_102913.1), *Candidatus* Nitrosopumilus koreensis strain AR1 (NR_102904.1)	92%	1	2	0	0	0
**9660**	LT896780	*Nitrosopumilus maritimus* strain SCM1 (NR_102913.1), *Candidatus* Nitrosopumilus koreensis strain AR1 (NR_102904.1)	87%	1	0	0	0	0
**9773**	LT896781	*Nitrosopumilus maritimus* strain SCM1 (NR_102913.1), *Candidatus* Nitrosopumilus koreensis strain AR1 (NR_102904.1)	94%	5	7	0	3	8
**9854**	LT896782	*Candidatus* Nitrososphaera gargensis (NR_102916.1)	77%	1	0	0	0	0
**9967**	LT896783	*Nitrosopumilus maritimus* strain SCM1 (NR_102913.1), *Candidatus* Nitrosopumilus koreensis strain AR1 (NR_102904.1)	90%	3	0	0	0	0
**10230**	LT896784	*Nitrosopumilus maritimus* strain SCM1 (NR_102913.1), *Candidatus* Nitrosopumilus koreensis strain AR1 (NR_102904.1)	98%	32	166	2	12	7
**10759**	LT896785	*Nitrosopumilus maritimus* strain SCM1 (NR_102913.1), *Candidatus* Nitrosopumilus koreensis strain AR1 (NR_102904.1)	92%	1	0	0	0	0
**11077 ***	LT896786	*Nitrosopumilus maritimus* strain SCM1 (NR_102913.1), *Candidatus* Nitrosopumilus koreensis strain AR1 (NR_102904.1)	100%	2854	7838	25	72	12
**11246**	LT896787	*Candidatus* Nitrosopumilus sp. HCA1 (KF957663.1)	92%	4	5	0	0	0
**11421**	LT896788	*Candidatus* Nitrosopelagicus brevis strain CN25 (CP007026.1)	93%	1	0	0	0	0
**11494**	LT896789	*Candidatus* Nitrosopelagicus brevis strain CN25 (CP007026.1)	95%	1	37	8	58	271
**11766**	LT896790	*Candidatus* Nitrosopumilus sp. HCA1 (KF957663.1)	99%	66	346	1	1	0
**11862**	LT896791	*Candidatus* Nitrosopelagicus brevis strain CN25 (CP007026.1)	100%	434	697	2	0	0

**11968 ***	LT896792	*Candidatus* Nitrosopumilus sp. HCA1 (KF957663.1), *Nitrosopumilus maritimus* strain NAOA6 (KT380502.1), *Candidatus* Nitrosomarinus catalina strain SPOT01 (CP021324.1)	100%	5025	19804	3	2	1
**12148**	LT896793	*Candidatus* Nitrosopumilus sp. HCA1 (KF957663.1)	98%	3	3	0	0	0
**12154**	LT896794	*Candidatus* Nitrosopumilus sp. NF5 (CP011070.1), *Candidatus* Nitrosopumilus sp. D3C (CP010868.1)	98%	24	3	0	1	1
**14018**	LT896795	*Candidatus* Nitrosopumilus sp. HCA1 (KF957663.1)	94%	1	5	0	0	0
**15090**	LT896796	*Candidatus* Nitrosopumilus sp. HCA1 (KF957663.1)	93%	1	0	0	0	0
**15267**	LT896797	*Candidatus* Nitrosopumilus sp. NF5 (CP011070.1), *Candidatus* Nitrosopumilus sp. D3C (CP010868.1)	95%	2	4	2	3	0
**15440**	LT896798	*Candidatus* Nitrosopumilus sp. NF5 (CP011070.1)	96%	12	52	2	14	1
**15912**	LT896799	*Candidatus* Nitrosopumilus sp. NF5 (CP011070.1,), *Candidatus* Nitrosopumilus sp. D3C (CP010868.1)	91%	1	3	0	2	1
**16209**	LT896800	*Candidatus* nitrosopumilus sp. NF5 (CP011070.1), *Candidatus* Nitrosopumilus sp. D3C (CP010868.1), *Candidatus* Nitrosopelagicus brevis strain CN25 (CP007026.1)	96%	7	54	2	22	37
**16282**	LT896801	*Candidatus* nitrosopumilus sp. NF5 (CP011070.1), *Candidatus* Nitrosopumilus sp. D3C (CP010868.1), *Candidatus* Nitrosopelagicus brevis strain CN25 (CP007026.1)	94%	1	0	0	0	0
**16366**	LT896802	*Candidatus* Nitrosopumilus sp. NF5 (CP011070.1), *Candidatus* Nitrosopumilus sp. D3C(CP010868.1)	97%	271	556	4	90	7
**16415**	LT896803	*Candidatus* Nitrosopumilus sp. HCA1 (KF957663.1)	94%	1	7	0	2	0
**16461**	LT896804	*Candidatus* Nitrosopelagicus brevis strain CN25 (CP007026.1)	95%	1	27	58	5	2
**16813**	LT896805	*Candidatus* Nitrosopumilus sp. HCA1 (KF957663.1)	96%	2	1	0	0	0
**17034**	LT896806	*Candidatus* Nitrosopumilus sp. NF5 (CP011070.1), *Candidatus* Nitrosopumilus sp. D3C (CP010868.1), *Candidatus* Nitrosopelagicus brevis strain CN25 (CP007026.1)	95%	1	1	1	0	0
**17072**	LT896807	*Candidatus* Nitrosopelagicus brevis strain CN25 (CP007026.1)	95%	1	36	35	2	0
**17460**	LT896808	*Candidatus* Nitrosopelagicus brevis strain CN25 (CP007026.1)	87%	1	0	0	0	0
**18055**	LT896809	*Candidatus* Nitrosopumilus sp. NF5 (CP011070.1), *Candidatus* Nitrosopumilus sp. D3C (CP010868.1)	90%	1	0	0	0	0

**18551**	LT896810	*Candidatus* Nitrosopumilus sp. NF5 (CP011070.1), *Candidatus* Nitrosopumilus sp. D3C (CP010868.1), *Candidatus* Nitrosopelagicus brevis strain CN25 (CP007026.1)	96%	4	55	5	81	1161
**18647**	LT896811	*Candidatus* Nitrosopelagicus brevis strain CN25 (CP007026.1)	96%	1	4	0	0	0
**19140**	LT896812	*Candidatus* nitrosopumilus sp. NF5 (CP011070.1), *Candidatus* Nitrosopumilus sp. D3C (CP010868.1), *Candidatus* Nitrosopelagicus brevis strain CN25 (CP007026.1)	91%	1	0	0	0	1
**19269**	LT896813	*Candidatus* Nitrosopumilus sp. NF5 (CP011070.1), *Candidatus* Nitrosopumilus sp. D3C (CP010868.1)	96%	1	24	0	119	868
**19595**	LT896814	*Candidatus* Nitrosopelagicus brevis strain CN25 (CP007026.1)	96%	2	90	12	77	700
**20099**	LT896815	*Candidatus* Nitrosopelagicus brevis strain CN25 (CP007026.1)	96%	1	3	1	0	0
**20204**	LT896816	*Candidatus* Nitrosopumilus sp. HCA1 (KF957663.1)	92%	1	9	0	1	0
**20233**	LT896817	*Candidatus* Nitrosopelagicus brevis strain CN25 (CP007026.1)	97%	1	5	1	0	1
**20356**	LT896818	*Candidatus* Nitrosopelagicus brevis strain CN25 (CP007026.1)	100%	1	544	2	6	0
**20746**	LT896819	*Candidatus* Nitrosopumilus sp. NF5 (CP011070.1), *Candidatus* Nitrosopumilus sp. D3C (CP010868.1)	98%	0	1019	75	255	49
**20748**	LT896820	*Candidatus* Nitrosopumilus sp. HCA1 (KF957663.1)	99%	0	433	1	4	0
**20762**	LT896821	*Candidatus* Nitrosopelagicus brevis strain CN25 (CP007026.1)	95%	0	20	4	2	0
**20799 ***	LT896822	*Candidatus* Nitrosopelagicus brevis strain CN25 (CP007026.1)	98%	0	1529	6	10	1
**20807**	LT896823	*Candidatus* Nitrosopumilus sp. HCA1 (KF957663.1)	96%	0	42	1	0	1
**20827**	LT896824	*Candidatus* Nitrosopumilus sp. HCA1 (KF957663.1)	92%	0	2	0	0	0
**20894**	LT896825	*Candidatus* Nitrosopelagicus brevis strain CN25 (CP007026.1)	92%	0	1	0	0	0
**20929**	LT896826	*Candidatus* Nitrosopumilus sp. NF5 (CP011070.1), *Candidatus* Nitrosopumilus sp. D3C (CP010868.1)	88%	0	1	0	0	0
**21166**	LT896827	*Candidatus* Nitrosopumilus sp. HCA1 (KF957663.1)	99%	0	1	0	0	0
**21174**	LT896828	*Candidatus* Nitrosopumilus sp. HCA1 (KF957663.1)	94%	0	9	0	0	1
**21189**	LT896829	*Candidatus* Nitrosopumilus sp. HCA1 (KF957663.1)	92%	0	1	0	0	0
**21195**	LT896830	*Candidatus* Nitrosopelagicus brevis strain CN25 (CP007026.1)	96%	0	4	1	0	5
**21201**	LT896831	*Candidatus* Nitrosopelagicus brevis strain CN25 (CP007026.1)	90%	0	1	0	1	0
**21292**	LT896832	*Candidatus* Nitrosopumilus sp. HCA1 (KF957663.1)	99%	0	31	0	1	2

**21318**	LT896833	*Candidatus* Nitrosopelagicus brevis strain CN25 (CP007026.1)	91%	0	1	0	0	0
**21374**	LT896834	*Candidatus* Nitrosopelagicus brevis strain CN25 (CP007026.1)	93%	0	2	0	0	0
**21377**	LT896835	*Candidatus* Nitrosopumilus sp. HCA1 (KF957663.1)	99%	0	20	0	1	2
**21394**	LT896836	*Candidatus* Nitrosopumilus sp. HCA1 (KF957663.1)	99%	0	573	0	2	1
**21482**	LT896837	*Candidatus* Nitrosopumilus sp. PS0 (KF957664.1), *Candidatus* Nitrosopumilus sp. HCA1 (KF957663.1)	91%	0	1	0	0	0
**21586**	LT896838	*Candidatus* Nitrosopelagicus brevis strain CN25 (CP007026.1)	99%	0	41	3	0	0
**21643**	LT896839	*Candidatus* Nitrosopumilus sp. NF5 (CP011070.1), *Candidatus* Nitrosopumilus sp. D3C (CP010868.1)	96%	0	25	4	39	51
**21664**	LT896840	*Candidatus* Nitrosopelagicus brevis strain CN25 (CP007026.1)	95%	0	9	0	5	4
**21710**	LT896841	*Candidatus* Nitrosopelagicus brevis strain CN25 (CP007026.1)	95%	0	3	0	1	0
**21720**	LT896842	*Candidatus* Nitrosopelagicus brevis strain CN25 (CP007026.1)	92%	0	1	0	0	0
**21750**	LT896843	*Candidatus* Nitrosopumilus sp. HCA1 (KF957663.1)	92%	0	3	0	2	0
**21773**	LT896844	*Candidatus* Nitrosopelagicus brevis strain CN25 (CP007026.1)	92%	0	31	0	2	0
**21813**	LT896845	*Candidatus* Nitrosopumilus sp. HCA1 (KF957663.1)	99%	1	25	0	0	0
**21834**	LT896846	*Candidatus* Nitrosopumilus sp. HCA1 (KF957663.1)	94%	0	36	0	0	1
**21937**	LT896847	*Candidatus* Nitrosopumilus sp. NF5 (CP011070.1),	89%	0	1	0	0	0
**22124**	LT896848	*Candidatus* Nitrosopelagicus brevis strain CN25 (CP007026.1)	94%	0	1	0	0	0
**22180**	LT896849	*Candidatus* Nitrosopumilus sp. HCA1 (KF957663.1)	92%	0	1	0	0	0
**22207**	LT896850	*Candidatus* Nitrosopumilus sp. NF5 (CP011070.1), *Candidatus* Nitrosopumilus sp. D3C (CP010868.1)	88%	0	1	1	1	2
**22249**	LT896851	*Candidatus* Nitrosopumilus sp. NF5 (CP011070.1), *Candidatus* Nitrosopumilus sp. D3C (CP010868.1)	97%	0	13	2	9	9
**22293**	LT896852	*Candidatus* Nitrosopelagicus brevis strain CN25 (HQ214501.1)	96%	0	31	26	2	23
**22337**	LT896853	*Candidatus* Nitrosopelagicus brevis strain CN25 (HQ214501.1)	95%	0	42	5	7	1
**22419**	LT896854	*Candidatus* Nitrosopumilus koreensis strain AR1 (NR_102904.1)	99%	0	90	1	8	7
**22430**	LT896855	*Candidatus* Nitrosopumilus sp. HCA1 (KF957663.1)	98%	0	221	0	1	1
**22474 ***	LT896856	*Candidatus* Nitrosopumilus sp. HCA1 (\KF957663.1), *Nitrosopumilus maritimus* strain NAOA6 (KT380502.1), *Candidatus* Nitrosomarinus catalina strain SPOT01 (CP021324.1)	98%	0	1980	190	125	87

**22536**	LT896857	*Candidatus* Nitrosopumilus sp. NF5 (CP011070.1), *Candidatus* Nitrosopumilus sp. D3C (CP010868.1)	96%	0	4	0	0	0
**22609**	LT896858	*Candidatus* Nitrosopumilus sp. NF5 (CP011070.1), *Candidatus* Nitrosopumilus sp. D3C (CP010868.1)	90%	0	103	17	80	129
**22615**	LT896859	*Candidatus* Nitrosopumilus sp. NF5 (CP011070.1), *Candidatus* Nitrosopumilus sp. D3C (CP010868.1)	90%	0	3	0	1	3
**22644**	LT896860	*Candidatus* Nitrosopumilus sp. NF5 (CP011070.1), *Candidatus* Nitrosopumilus sp. D3C (CP010868.1)	97%	0	2	1	0	0
**22714**	LT896861	*Candidatus* Nitrosopumilus sp. NF5 (CP011070.1), *Candidatus* Nitrosopumilus sp. D3C (CP010868.1)	91%	0	1	0	1	0
**22749**	LT896862	*Candidatus* Nitrosopumilus sp. HCA1 (KF957663.1)	99%	0	5	0	5	1
**22823**	LT896863	*Candidatus* Nitrosopumilus sp. HCA1 (KF957663.1)	100%	1	174	0	8	4
**22830 ***	LT896864	*Candidatus* Nitrosopelagicus brevis strain CN25 (HQ214501.1)	100%	0	35809	0	3	1
**22845 ***	LT896865	*Candidatus* Nitrosopelagicus brevis strain CN25 (HQ214501.1)	99%	0	1571	14	12	2
**22849**	LT896866	*Candidatus* Nitrosopelagicus brevis strain CN25 (HQ214501.1)	98%	0	458	34	8	1
**22890 ***	LT896867	*Candidatus* Nitrosopelagicus brevis strain CN25 (HQ214501.1)	96%	0	2489	23	28	6
**22972**	LT896868	*Candidatus* Nitrosopumilus sp. NF5 (CP011070.1), *Candidatus* Nitrosopumilus sp. D3C (CP010868.1)	97%	0	24	3	6	0
**22977**	LT896869	*Candidatus* Nitrosopumilus sp. NF5 (CP011070.1), *Candidatus* Nitrosopumilus sp. D3C (CP010868.1)	91%	0	216	32	528	7285
**23162**	LT896870	*Candidatus* Nitrosopumilus sp. HCA1 (KF957663.1)	93%	0	1	0	0	0
**23218**	LT896871	*Candidatus* Nitrosopelagicus brevis strain CN25 (HQ214501.1)	95%	0	5	2	3	1
**23310**	LT896872	*Candidatus* Nitrosopelagicus brevis strain CN25 (HQ214501.1)	98%	0	7	1	1	0
**23351 ***	LT896873	*Candidatus* Nitrosopelagicus brevis strain CN25 (HQ214501.1)	100%	0	2450	1	2	0
**23536**	LT896874	*Candidatus* Nitrosopelagicus brevis strain CN25 (HQ214501.1)	96%	0	27	0	8	6
**23612**	LT896875	*Candidatus* Nitrosopumilus sp. NF5 (CP011070.1), *Candidatus* Nitrosopumilus sp. D3C (CP010868.1), *Candidatus* Nitrosopelagicus brevis strain CN25 (CP007026.1)	91%	0	106	15	58	18
**23840**	LT896876	*Candidatus* Nitrosopumilus sp. HCA1 (KF957663.1)	97%	0	10	1	7	0
**23852**	LT896877	*Candidatus* Nitrosopelagicus brevis strain CN25 (HQ214501.1)	97%	0	81	11	12	3
**23915**	LT896878	*Candidatus* Nitrosopelagicus brevis strain CN25 (HQ214501.1)	97%	0	5	1	0	0

**24003 ***	LT896879	*Candidatus* Nitrosopelagicus brevis strain CN25 (HQ214501.1)	94%	0	15797	0	8	3
**24054**	LT896880	*Candidatus* Nitrosopelagicus brevis strain CN25 (HQ214501.1)	98%	0	38	5	41	20
**24090**	LT896881	*Candidatus* Nitrosopumilus sp. HCA1 (KF957663.1)	99%	0	7	0	5	0
**24305**	LT896882	*Candidatus* Nitrosopumilus sp. NF5 (CP011070.1), *Candidatus* Nitrosopumilus sp. D3C (CP010868.1), *Candidatus* Nitrosopelagicus brevis strain CN25 (CP007026.1)	92%	0	6	0	2	0
**24312**	LT896883	*Candidatus* Nitrosopumilus sp. HCA1 (KF957663.1)	100%	0	4	0	2	1
**24395**	LT896884	*Candidatus* Nitrosopelagicus brevis strain CN25 (HQ214501.1)	95%	0	1	0	0	0
**24403**	LT896885	*Candidatus* Nitrosopelagicus brevis strain CN25 (HQ214501.1)	97%	0	1	0	0	1
**24440**	LT896886	*Candidatus* Nitrosopelagicus brevis strain CN25 (HQ214501.1)	97%	0	6	0	2	0
**24467**	LT896887	*Candidatus* Nitrosopumilus sp. HCA1 (KF957663.1)	90%	0	58	1	9	5
**24703**	LT896888	*Candidatus* Nitrosopumilus sp. NF5 (CP011070.1), *Candidatus* Nitrosopumilus sp. D3C (CP010868.1)	90%	0	2	0	0	0
**24719**	LT896889	*Candidatus* Nitrosopumilus sp. HCA1 (KF957663.1), *Candidatus* Nitrosopumilus koreensis AR1 strain (NR_102904.1)	94%	0	2	0	1	0
**24879**	LT896890	*Candidatus* Nitrosopumilus sp. HCA1 (KF957663.1), *Candidatus* Nitrosopumilus koreensis AR1 strain (NR_102904.1)	92%	0	2	0	0	0
**24983**	LT896891	*Candidatus* Nitrosopumilus sp. HCA1 (KF957663.1), *Candidatus* Nitrosopumilus koreensis AR1 strain (NR_102904.1)	99%	0	90	0	0	1
**24989**	LT896892	*Candidatus* Nitrosopelagicus brevis strain CN25 (CP007026.1)	92%	0	2	1	1	2
**25118**	LT896893	*Candidatus* Nitrosopumilus sp. HCA1 (KF957663.1), *Nitrosopumilus maritimus* SCM1 strain SCM1 16S (NR_102913.1)	92%	0	1	0	0	0
**25130**	LT896894	*Candidatus* Nitrosopumilus sp. HCA1 (KF957663.1), *Candidatus* Nitrosopumilus koreensis AR1 strain (NR_102904.1)	95%	0	1	0	0	0
**25157**	LT896895	Uncultured *Candidatus* Nitrosopumilus sp. clone A15 (KT335988.1)	97%	0	6	2	5	119
**25177**	LT896896	*Candidatus* Nitrosopelagicus brevis strain CN25 (CP007026.1)	91%	0	1	0	0	0
**25204**	LT896897	*Candidatus* Nitrosopumilus sp. PS0 (KF957664.1), *Nitrosopumilus* sp. DDS1 (KR737579.1)	96%	0	1	0	0	0
**25222**	LT896898	*Candidatus* Nitrosopumilus sp. HCA1 (KF957663.1), *Nitrosopumilus maritimus* SCM1 strain (NR_102913.1)	84%	0	1	0	0	0
**25287**	LT896899	*Candidatus* Nitrosopumilus sp. NF5 (CP011070.1), *Candidatus* Nitrosopumilus sp. D3C (CP010868.1)	96%	0	45	27	30	28

**25297**	LT896900	*Candidatus* Nitrosopumilus sp. NF5 (CP011070.1), *Candidatus* Nitrosopumilus sp. D3C (CP010868.1)	86%	0	2	0	0	0
**25303**	LT896901	*Candidatus* Nitrosopumilus sp. HCA1 (KF957663.1), *Nitrosopumilus maritimus* SCM1 strain SCM1 (NR_102913.1)	99%	0	328	0	2	1
**25452**	LT896902	*Candidatus* Nitrosopumilus sp. HCA1 (KF957663.1), *Nitrosopumilus maritimus* SCM1 strain SCM1 (NR_102913.1)	93%	0	1	0	0	0
**25470**	LT896903	*Candidatus* Nitrosopelagicus brevis strain CN25 (CP007026.1)	91%	0	1	0	0	0
**25587**	LT896904	*Candidatus* Nitrosopumilus sp. HCA1 (KF957663.1), *Nitrosopumilus maritimus* SCM1 strain SCM1 (NR_102913.1)	99%	0	184	1	1	1
**25758**	LT896905	*Candidatus* Nitrosopumilus sp. HCA1 (KF957663.1), *Nitrosopumilus maritimus* strain SCM1 (NR_102913.1)	94%	0	1	0	0	0
**26263**	LT896906	*Candidatus* Nitrosopelagicus brevis strain CN25 (CP007026.1)	94%	0	4	0	3	0
**26425 ***	LT896907	*Candidatus* Nitrosopelagicus brevis strain CN25 (CP007026.1)	98%	0	3473	40	33	12
**26608**	LT896908	*Candidatus* Nitrosopumilus sp. NF5 (CP011070.1), *Candidatus* Nitrosopumilus sp. D3C (CP010868.1)	98%	0	53	1	0	1
**26612 ***	LT896909	*Candidatus* Nitrosopumilus sp. HCA1 (KF957663.1), *Nitrosopumilus maritimus* strain SCM1 (NR_102913.1), *Candidatus* Nitrosomarinus catalina strain SPOT01 (CP021324.1)	100%	0	1162 5	2	3	0
**26903**	LT896910	*Candidatus* Nitrosopumilus sp. HCA1 (KF957663.1), *Nitrosopumilus maritimus* strain SCM1 (NR_102913.1)	93%	0	2	0	0	0
**26906**	LT896911	*Candidatus* Nitrosopumilus sp. HCA1 (KF957663.1), *Nitrosopumilus maritimus* strain SCM1 (NR_102913.1)	92%	0	15	0	0	1
**27220**	LT896912	*Candidatus* Nitrosopumilus sp. NF5 (CP011070.1), *Candidatus* Nitrosopumilus sp. D3C (CP010868.1)	88%	0	1	0	0	0
**27340 ***	LT896913	*Candidatus* Nitrosopumilus sp. HCA1 (KF957663.1), *Nitrosopumilus maritimus* strain SCM1 (NR_102913.1), *Candidatus* Nitrosomarinus catalina strain SPOT01 (CP021324.1)	98%	0	1230 4	465	153	77
**27384**	LT896914	*Candidatus* Nitrosopumilus sp. HCA1 (KF957663.1), *Nitrosopumilus maritimus* strain SCM1 (NR_102913.1)	98%	0	2960	847	19	9
**27490**	LT896915	*Candidatus* Nitrosopelagicus brevis strain CN25 (CP007026.1)	100%	0	596	8	16	13
**27493**	LT896916	*Candidatus* Nitrosopumilus sp. NF5 (CP011070.1), *Candidatus* Nitrosopumilus sp. D3C (CP010868.1)	95%	0	1	0	0	0

**27514**	LT896917	*Candidatus* Nitrosopumilus sp. HCA1 (KF957663.1), *Nitrosopumilus maritimus* strain SCM1 (NR_102913.1), *Candidatus* Nitrosomarinus catalina strain SPOT01 (CP021324.1)	99%	0	279	6	3	2
**27538 ***	LT896918	*Candidatus* Nitrosopumilus sp. HCA1 (KF957663.1), *Nitrosopumilus maritimus* strain SCM1 (NR_102913.1), *Candidatus* Nitrosomarinus catalina strain SPOT01 (CP021324.1)	99%	0	1107	19	8	11
**27557**	LT896919	*Candidatus* Nitrosopumilus sp. HCA1 (KF957663.1), *Nitrosopumilus maritimus* strain SCM1 (NR_102913.1), *Candidatus* Nitrosomarinus catalina strain SPOT01 (CP021324.1)	99%	0	230	1	0	1
**27608 ***	LT896920	*Candidatus* Nitrosopumilus sp. HCA1 (KF957663.1), *Nitrosopumilus maritimus* strain SCM1 (NR_102913.1), *Candidatus* Nitrosomarinus catalina strain SPOT01 (CP021324.1)	100%	0	2222 8	3	22	34
**27622**	LT896921	*Candidatus* Nitrosopelagicus brevis strain CN25 (CP007026.1)	90%	0	1	0	1	0
**27699**	LT896922	*Candidatus* Nitrosopelagicus brevis strain CN25 (CP007026.1)	95%	0	4	0	12	0
**27750**	LT896923	*Candidatus* Nitrosopelagicus brevis strain CN25 (CP007026.1)	92%	0	1	1	8	1
**27841**	LT896924	*Candidatus* Nitrosopumilus sp. NF5 (CP011070.1), *Candidatus* Nitrosopumilus sp. D3C (CP010868.1)	96%	0	1	1	0	3
**27883**	LT896925	*Candidatus* Nitrosopumilus sp. HCA1 (KF957663.1)	99%	0	140	0	14	3
**28136 ***	LT896926	*Candidatus* Nitrosopelagicus brevis strain CN25 (CP007026.1)	100%	0	3566	879	31	2
**28295**	LT896927	*Candidatus* Nitrosopumilus sp. NF5 (CP011070.1), *Candidatus* Nitrosopumilus sp. D3C (CP010868.1)	91%	0	4	0	0	0
**28423**	LT896928	*Candidatus* Nitrosopumilus sp. NF5 (CP011070.1), *Candidatus* Nitrosopumilus sp. D3C (CP010868.1)	86%	0	1	0	0	0
**28501**	LT896929	*Candidatus* Nitrosopumilus sp. NF5 (CP011070.1), *Candidatus* Nitrosopumilus sp. D3C (CP010868.1)	89%	0	1	0	0	0
**28526**	LT896930	*Candidatus* Nitrosopumilus sp. HCA1 (KF957663.1)	99%	0	720	1	2	3
**28655**	LT896931	*Candidatus* Nitrosopumilus sp. HCA1 (KF957663.1)	93%	0	1	0	0	0
**28736**	LT896932	*Candidatus* Nitrosopelagicus brevis strain CN25 (CP007026.1)	93%	0	1	0	0	0
**28846**	LT896933	*Candidatus* Nitrosopelagicus brevis strain CN25 (CP007026.1)	92%	0	1	0	1	0
**29178**	LT896934	*Candidatus* Nitrosopelagicus brevis strain CN25 (CP007026.1)	96%	0	159	68	51	3
**29649**	LT896935	*Candidatus* Nitrosopumilus sp. NF5 CP011070.1), *Candidatus* Nitrosopumilus sp. D3C (CP010868.1)	91%	0	1	0	0	0

**29805 ***	LT896936	*Candidatus* Nitrosopelagicus brevis strain CN25 (CP007026.1)	98%	0	6834	4342	1465	266
**29845 ***	LT896937	*Candidatus* Nitrosopumilus sp. HCA1 (KF957663.1), *Candidatus* Nitrosomarinus catalina strain SPOT01 (CP021324.1), *Candidatus* Nitrosomarinus catalina strain SPOT01 (CP021324.1)	100%	0	1114 1	2	27	7
**29875**	LT896938	*Candidatus* Nitrosopumilus sp. HCA1 (KF957663.1)	93%	0	1	0	0	0
**29931**	LT896939	*Candidatus* Nitrosopelagicus brevis strain CN25 (CP007026.1)	89%	0	1	0	0	0
**30156**	LT896940	*Candidatus* Nitrosopumilus sp. NF5 (CP011070.1)	98%	0	37	4	5	0
**30552**	LT896941	*Candidatus* Nitrosopumilus sp. NF5 (CP011070.1), *Candidatus* Nitrosopumilus sp. D3C (CP010868.1)	98%	0	159	151	15	2
**30631**	LT896942	*Candidatus* Nitrosopelagicus brevis strain CN25 (CP007026.1)	94%	0	1	0	1	0
**30657 ***	LT896943	*Candidatus* Nitrosopumilus sp. HCA1 (KF957663.1), *Candidatus* Nitrosomarinus catalina strain SPOT01 (CP021324.1), *Candidatus* Nitrosomarinus catalina strain SPOT01 (CP021324.1)	99%	0	2275 5	2920	788	353
**30812**	LT896944	*Candidatus* Nitrosopumilus sp. NF5 (CP011070.1), *Candidatus* Nitrosopumilus sp. D3C (CP010868.1)	97%	0	110	146	6	3
**30944**	LT896945	*Nitrosopumilus maritimus* strain NAOA6 (KT380502.1), *Candidatus* Nitrosopumilus sp. HCA1 (KF957663.1)	98%	0	4	2	1	1
**30970 ***	LT896946	*Candidatus* Nitrosopumilus sp. HCA1 (KF957663.1), *Candidatus* Nitrosomarinus catalina strain SPOT01 (CP021324.1), *Candidatus* Nitrosomarinus catalina strain SPOT01 (CP021324.1)	100%	0	1275 9	416	29	38
**31027**	LT896947	*Candidatus* Nitrosopelagicus brevis strain CN25 (CP007026.1)	93%	0	1	0	0	0
**31041**	LT896948	*Candidatus* Nitrosopelagicus brevis strain CN25 (CP007026.1)	98%	0	5	4	1	0
**31121**	LT896949	*Candidatus* Nitrosopelagicus brevis strain CN25 (CP007026.1)	90%	0	1	0	0	0
**31178**	LT896950	*Nitrosopumilus maritimus* strain NAOA6 (KT380502.1), *Candidatus* Nitrosopumilus sp. HCA1 (KF957663.1)	99%	1	527	116	52	21
**31280**	LT896951	*Candidatus* Nitrosopelagicus brevis strain CN25 (CP007026.1)	91%	0	1	0	0	0
**31377**	LT896952	*Candidatus* Nitrosopelagicus brevis strain CN25 (CP007026.1)	96%	0	1	0	1	1
**31558**	LT896953	*Nitrosopumilus maritimus* strain NAOA6 (KT380502.1), *Candidatus* Nitrosopumilus sp. HCA1 (KF957663.1)	92%	0	1	0	0	0
**31911 ***	LT896954	*Candidatus* Nitrosopumilus sp. HCA1 (KF957663.1), *Candidatus* Nitrosomarinus catalina strain SPOT01 (CP021324.1), *Candidatus* Nitrosomarinus catalina strain SPOT01 (CP021324.1)	100%	0	5363	5159	1	0

**32039**	LT896955	*Nitrosopumilus maritimus* strain NAOA6 (KT380502.1), *Candidatus* Nitrosopumilus sp. HCA1 (KF957663.1)	97%	2	8	0	2	0
**32155**	LT896956	*Nitrosopumilus maritimus* strain NAOA6 (KT380502.1), *Candidatus* Nitrosopumilus sp. HCA1 (KF957663.1)	97%	0	337	4	76	122
**32164**	LT896957	*Candidatus* Nitrosopelagicus brevis strain CN25 (CP007026.1)	99%	0	9	1	0	0
**32190**	LT896958	*Nitrosopumilus maritimus* strain NAOA6 (KT380502.1), *Candidatus* Nitrosopumilus sp. HCA1 (KF957663.1)	99%	0	413	1	8	4
**32270**	LT896959	*Nitrosopumilus* sp. DDS1 (KR737579.1), *Candidatus* Nitrosopumilus sp. NF5 (CP011070.1), *Candidatus* Nitrosopumilus sp. D3C (CP010868.1), *Candidatus* Nitrosopelagicus brevis strain CN25 (CP007026.1)	96%	0	1	0	0	6
**32373**	LT896960	*Candidatus* Nitrosopelagicus brevis strain CN25 (CP007026.1)	98%	0	3154	1	46	7
**32375**	LT896961	*Nitrosopumilus* sp. DDS1 (KR737579.1), *Candidatus* Nitrosopumilus sp. NF5 (CP011070.1), *Candidatus* Nitrosopumilus sp. D3C (CP010868.1), *Candidatus* Nitrosopelagicus brevis strain CN25 (CP007026.1)	96%	0	75	1	175	147
**32402**	LT896962	*Candidatus* Nitrosopelagicus brevis strain CN25 (CP007026.1)	95%	0	1	3	9	187
**32403**	LT896963	*Candidatus* Nitrosopelagicus brevis strain CN25 (CP007026.1)	99%	0	18	1	0	0
**32417**	LT896964	*Nitrosopumilus* sp. DDS1 (KR737579.1), *Candidatus* Nitrosopumilus sp. NF5 (CP011070.1), *Candidatus* Nitrosopumilus sp. D3C (CP010868.1), *Candidatus* Nitrosopelagicus brevis strain CN25 (CP007026.1)	98%	0	121	1	6	6
**32433**	LT896965	*Nitrosopumilus maritimus* strain SCM1 (NR_102913.1), *Candidatus* Nitrosopumilus koreensis strain AR1 (NR_102904.1), *Candidatus* Nitrosopumilus sp. AR2 (CP003843.1), *Candidatus* Nitrosopumilus koreensis AR1 (CP003842.1), *Nitrosopumilus maritimus* SCM1 (CP000866.1)	92%	0	0	1	0	0
**32434**	LT896966	*Nitrosopumilus maritimus* strain SCM1 (NR_102913.1), *Candidatus* Nitrosopumilus koreensis strain AR1 (NR_102904.1), *Candidatus* Nitrosopumilus sp. AR2 (CP003843.1), *Candidatus* Nitrosopumilus koreensis AR1 (CP003842.1), *Nitrosopumilus maritimus* SCM1 (CP000866.1)	98%	0	42	1	8	3

**32452**	LT896967	*Nitrosopumilus* sp. DDS1 (KR737579.1), *Candidatus* Nitrosopumilus sp. NF5 (CP011070.1), *Candidatus* Nitrosopumilus sp. D3C (CP010868.1), *Candidatus* Nitrosopelagicus brevis strain CN25 (CP007026.1)	93%	0	0	1	1	6
**32455**	LT896968	*Candidatus* Nitrosopelagicus brevis strain CN25 (CP007026.1)	89%	0	0	1	0	0
**32510**	LT896969	*Candidatus* Nitrosopelagicus brevis strain CN25 (CP007026.1)	95%	0	1	1	0	6
**32573**	LT896970	*Nitrosopumilus* sp. DDS1 (KR737579.1), *Candidatus* Nitrosopumilus sp. NF5 (CP011070.1), *Candidatus* Nitrosopumilus sp. D3C (CP010868.1),	96%	0	34	1	64	163
**32591**	LT896971	*Candidatus* Nitrosopelagicus brevis strain CN25 (CP007026.1)	95%	0	8	1	14	7
**32599**	LT896972	*Candidatus* Nitrosopelagicus brevis strain CN25 (CP007026.1), *Nitrosopumilus* sp. DDS1 (KR737579.1), *Candidatus* Nitrosopumilus sp. NF5 (CP011070.1), *Candidatus* Nitrosopumilus sp. D3C (CP010868.1)	96%	0	65	1	27	41
**32621**	LT896973	*Candidatus* Nitrosopelagicus brevis strain CN25 (CP007026.1)	93%	0	1	2	1	0
**32642**	LT896974	*Candidatus* Nitrosopelagicus brevis strain CN25 (CP007026.1), *Nitrosopumilus* sp. DDS1 (KR737579.1), *Candidatus* Nitrosopumilus sp. NF5 (CP011070.1), *Candidatus* Nitrosopumilus sp. D3C (CP010868.1), *Nitrosopumilus maritimus* strain SCM1 (NR_102913.1), *Candidatus* Nitrosopumilus koreensis strain AR1 (NR_102904.1), *Candidatus* Nitrosopumilus sp. AR2 (CP003843.1), *Candidatus* Nitrosopumilus koreensis AR1 (CP003842.1), *Nitrosopumilus maritimus* SCM1 (CP000866.1)	95%	0	0	1	0	14
**32648**	LT896975	*Candidatus* Nitrosopelagicus brevis strain CN25 (CP007026.1)	97%	0	1559	1	1198	381
**32662**	LT896976	*Candidatus* Nitrosopelagicus brevis strain CN25 (CP007026.1)	95%	0	6	1	3	0
**32724**	LT896977	*Nitrosopumilus* sp. DDS1 (KR737579.1), *Candidatus* Nitrosopumilus sp. NF5 (CP011070.1), *Candidatus* Nitrosopumilus sp. D3C (CP010868.1), *Candidatus* Nitrosopelagicus brevis strain CN25 (CP007026.1), *Nitrosopumilus maritimus* strain SCM1 (NR_102913.1), *Candidatus* Nitrosopumilus koreensis strain AR1 (NR_102904.1), *Candidatus* Nitrosopumilus sp. AR2(CP003843.1), *Candidatus* Nitrosopumilus koreensis AR1 (CP003842.1), *Nitrosopumilus maritimus* SCM1 (CP000866.1)	96%	0	10	2	41	43

**32759**	LT896978	*Candidatus* Nitrosopelagicus brevis strain CN25 (CP007026.1)	95%	0	727	2	17	8
**32769**	LT896979	*Candidatus* Nitrosopelagicus brevis strain CN25 (CP007026.1), *Nitrosopumilus* sp. DDS1 (KR737579.1), *Candidatus* Nitrosopumilus sp. NF5 (CP011070.1), *Candidatus* Nitrosopumilus sp. D3C (CP010868.1)	95%	0	3	1	13	49
**32842**	LT896980	*Nitrosopumilus maritimus* SCM1 strain SCM1 (NR_102913.1), *Candidatus* Nitrosopumilus koreensis strain AR1 (NR_102904.1), *Candidatus* Nitrosopumilus sp. AR2 (CP003843.1), *Candidatus* Nitrosopumilus koreensis AR1 (CP003842.1), *Nitrosopumilus maritimus* SCM1 (CP000866.1), *Nitrosopumilus* sp. DDS1 (KR737579.1), *Candidatus* Nitrosopumilus sp. NF5 (CP011070.1), *Candidatus* Nitrosopumilus sp. D3C (CP010868.1)	99%	0	20	1	0	1
**32932**	LT896981	*Candidatus* Nitrosopelagicus brevis strain CN25 (CP007026.1)	89%	0	0	1	0	1
**33011**	LT896982	*Nitrosopumilus maritimus* SCM1 strain SCM1 (NR_102913.1), *Candidatus* Nitrosopumilus koreensis strain AR1 (NR_102904.1), *Candidatus* Nitrosopumilus sp. AR2 (CP003843.1), *Candidatus* Nitrosopumilus koreensis AR1 (CP003842.1), *Nitrosopumilus maritimus* SCM1 (CP000866.1), *Nitrosopumilus* sp. DDS1 (KR737579.1), *Candidatus* Nitrosopumilus sp. NF5 (CP011070.1), *Candidatus* Nitrosopumilus sp. D3C (CP010868.1)	98%	0	1189	1	3	1
**33012**	LT896983	*Nitrosopumilus maritimus* SCM1 strain SCM1 (NR_102913.1), *Candidatus* Nitrosopumilus koreensis strain AR1 (NR_102904.1), *Candidatus* Nitrosopumilus sp. AR2 (CP003843.1), *Candidatus* Nitrosopumilus koreensis AR1 (CP003842.1), *Nitrosopumilus maritimus* SCM1 (CP000866.1), *Nitrosopumilus* sp. DDS1 (KR737579.1), *Candidatus* Nitrosopumilus sp. NF5 (CP011070.1), *Candidatus* Nitrosopumilus sp. D3C (CP010868.1)	99%	0	497	2	18	6

**33021**	LT896984	*Nitrosopumilus maritimus* SCM1 strain SCM1 (NR_102913.1), *Candidatus* Nitrosopumilus koreensis strain AR1 (NR_102904.1), *Candidatus* Nitrosopumilus sp. AR2 (CP003843.1), *Candidatus* Nitrosopumilus koreensis AR1 (CP003842.1), *Nitrosopumilus maritimus* SCM1 (CP000866.1), *Nitrosopumilus* sp. DDS1 (KR737579.1), *Candidatus* Nitrosopumilus sp. NF5 (CP011070.1), *Candidatus* Nitrosopumilus sp. D3C (CP010868.1)	100%	0	10171	1	63	13
**33037**	LT896985	*Nitrosopumilus maritimus* SCM1 strain SCM1 (NR_102913.1), *Candidatus* Nitrosopumilus koreensis strain AR1 (NR_102904.1), *Candidatus* Nitrosopumilus sp. AR2 (CP003843.1), *Candidatus* Nitrosopumilus koreensis AR1 (CP003842.1), *Nitrosopumilus maritimus* SCM1 (CP000866.1), *Nitrosopumilus* sp. DDS1 (KR737579.1), *Candidatus* Nitrosopumilus sp. NF5 (CP011070.1), *Candidatus* Nitrosopumilus sp. D3C (CP010868.1)	96%	0	2	1	9	7
**33083**	LT896986	*Candidatus* Nitrosopelagicus brevis strain CN25 (CP007026.1)	100%	0	1101	1	1	0
**33089**	LT896987	*Candidatus* Nitrosopelagicus brevis strain CN25 (CP007026.1)	96%	0	2	1	1	4
**33102**	LT896988	*Candidatus* Nitrosopelagicus brevis strain CN25 (CP007026.1)	97%	0	509	1	23	11
**33127**	LT896989	*Candidatus* Nitrosopelagicus brevis strain CN25 (CP007026.1)	92%	0	0	1	0	0
**33133**	LT896990	*Candidatus* Nitrosopelagicus brevis strain CN25 (CP007026.1)	96%	0	1386	1	318	18
**33230**	LT896991	*Candidatus* Nitrosopelagicus brevis strain CN25 (CP007026.1)	98%	0	54	1	34	10
**33257**	LT896992	*Candidatus* Nitrosopelagicus brevis strain CN25 (CP007026.1)	98%	0	5020	0	42	147
**33292**	LT896993	*Candidatus* Nitrosopelagicus brevis strain CN25 (CP007026.1)	96%	0	58	0	4	17
**33420**	LT896994	*Candidatus* Nitrosopelagicus brevis strain CN25 (CP007026.1)	99%	0	1100	0	868	62
**33436**	LT896995	*Nitrosopumilus maritimus* SCM1 strain SCM1 (NR_102913.1), *Candidatus* Nitrosopumilus koreensis strain AR1 (NR_102904.1), *Candidatus* Nitrosopumilus sp. AR2 (CP003843.1), *Candidatus* Nitrosopumilus koreensis AR1 (CP003842.1), *Nitrosopumilus maritimus* SCM1 (CP000866.1), *Nitrosopumilus* sp. DDS1 (KR737579.1)	94%	0	2	0	0	0

**33475**	LT896996	*Nitrosopumilus maritimus* SCM1 strain SCM1 (NR_102913.1), *Candidatus* Nitrosopumilus koreensis strain AR1 (NR_102904.1), *Candidatus* Nitrosopumilus sp. AR2 (CP003843.1), *Candidatus* Nitrosopumilus koreensis AR1 (CP003842.1), *Nitrosopumilus maritimus* SCM1 (CP000866.1), *Nitrosopumilus* sp. DDS1 (KR737579.1)	99%	0	422	0	110	9
**33501**	LT896997	*Candidatus* Nitrosopelagicus brevis strain CN25 (CP007026.1)	95%	0	4	0	14	10
**33560**	LT896998	*Candidatus* Nitrosopelagicus brevis strain CN25 (CP007026.1)	97%	0	1327	0	106	17
**33609**	LT896999	*Candidatus* Nitrosopelagicus brevis strain CN25 (CP007026.1), *Nitrosopumilus* sp. DDS1 (KR737579.1), *Candidatus* Nitrosopumilus sp. NF5 (CP011070.1), *Candidatus* Nitrosopumilus sp. D3C (CP010868.1)	96%	0	22	0	20	19
**33665**	LT897000	*Candidatus* Nitrosopelagicus brevis strain CN25 (CP007026.1)	98%	0	7	0	21	0
**33682**	LT897001	*Candidatus* Nitrosopelagicus brevis strain CN25 (CP007026.1)	95%	0	93	0	13	6
**33719**	LT897002	*Candidatus* Nitrosopelagicus brevis strain CN25 (CP007026.1)	100%	0	1733	0	51	9
**33744**	LT897003	*Candidatus* Nitrosopelagicus brevis strain CN25 (CP007026.1)	95%	0	772	0	40	23
**33748**	LT897004	*Candidatus* Nitrosopelagicus brevis strain CN25 (CP007026.1)	98%	0	7961	0	217	36
**33775**	LT897005	*Candidatus* Nitrosopelagicus brevis strain CN25 (CP007026.1)	97%	0	229	0	14	2
**33801**	LT897006	Nitrosopumilus sp. DDS1 (CP011070.1), *Candidatus* Nitrosopumilus sp. D3C (CP010868.1)	86%	0	1	0	1	1
**33811**	LT897007	*Candidatus* Nitrosopumilus sp. HCA1 (KF957663.1), *Nitrosopumilus maritimus* strain SCM1 (NR_102913.1)	100%	0	1321<br>7	0	90	19
**33869**	LT897008	*Candidatus* Nitrosopelagicus brevis strain CN25 (CP007026.1)	98%	1	124	0	16	9
**33880**	LT897009	*Candidatus* Nitrosopelagicus brevis strain CN25 (CP007026.1)	98%	0	98	0	40	4
**33881**	LT897010	*Nitrosopumilus* sp. DDS1 (KR737579.1), *Candidatus* Nitrosopumilus sp.NF5 (CP011070.1)	88%	0	1	0	0	0
**33887**	LT897011	Nitrosopumilus sp. DDS1 (KR737579.1), *Candidatus* Nitrosopumilus sp.NF5 (CP011070.1)	99%	0	69	0	45	4
**33907**	LT897012	*Candidatus* Nitrosopelagicus brevis strain CN25 (CP007026.1)	99%	1	174	1	251	29
**33911**	LT897013	*Candidatus* Nitrosopelagicus brevis strain CN25 (CP007026.1)	94%	1	22	0	88	42
**33925**	LT897014	*Candidatus* Nitrosopelagicus brevis strain CN25 (CP007026.1), *Nitrosopumilus* sp. DDS1 (KR737579.1)	94%	0	1	0	0	0

**33988**	LT897015	*Candidatus* Nitrosopumilus sp. HCA1 (KF957663.1), *Nitrosopumilus maritimus* strain SCM1 (NR_102913.1)	98%	0	3486	0	1685	4
**34020**	LT897016	*Candidatus* Nitrosopelagicus brevis strain CN25 (CP007026.1)	95%	0	47	0	13	3
**34030**	LT897017	*Candidatus* Nitrosopumilus sp. HCA1 (KF957663.1), *Nitrosopumilus maritimus* strain SCM1 (NR_102913.1)	93%	0	3	0	0	0
**34085**	LT897018	*Candidatus* Nitrosopelagicus brevis strain CN25 (CP007026.1)	95%	0	12	0	19	14
**34088**	LT897019	*Candidatus* Nitrosopelagicus brevis strain CN25 (CP007026.1)	96%	0	164	0	92	2
**34173**	LT897020	*Candidatus* Nitrosopelagicus brevis strain CN25 (CP007026.1)	96%	0	18	0	3	9
**34185**	LT897021	*Candidatus* Nitrosopelagicus brevis strain CN25 (CP007026.1)	96%	0	744	0	60	29
**34187**	LT897022	*Candidatus* Nitrosopelagicus brevis strain CN25 (CP007026.1)	100%	0	14009	0	4589	24
**34201**	LT897023	*Candidatus* Nitrosopumilus sp. HCA1 (KF957663.1), *Nitrosopumilus maritimus* strain SCM1 (NR_102913.1)	100%	0	9788	0	14	5
**34255**	LT897024	*Nitrosopumilus* sp. DDS1 (KR737579.1), *Candidatus* Nitrosopumilus sp. NF5 (CP011070.1)	96%	0	13	0	14	8
**34271**	LT897025	*Candidatus* Nitrosopelagicus brevis strain CN25 (CP007026.1)	96%	0	366	0	360	11
**34273**	LT897026	*Candidatus* Nitrosopelagicus brevis strain CN25 (CP007026.1)	97%	0	7	0	9	1
**34317**	LT897027	*Nitrosopumilus* sp. DDS1 (KR737579.1), *Candidatus* Nitrosopumilus sp. NF5 (CP011070.1)	97%	0	105	0	225	15
**34344**	LT897028	*Candidatus* Nitrosopelagicus brevis strain CN25 (CP007026.1)	95%	0	71	0	123	12
**34366**	LT897029	*Candidatus* Nitrosopelagicus brevis strain CN25 (CP007026.1)	92%	0	1	0	2	0
**34385**	LT897030	*Nitrosopumilus* sp. DDS1 (KR737579.1), *Candidatus* Nitrosopumilus sp. NF5 (CP011070.1)	95%	0	6	0	5	5
**34388**	LT897031	*Candidatus* Nitrosopelagicus brevis strain CN25 (CP007026.1)	96%	0	34	0	142	110
**34397**	LT897032	*Candidatus* Nitrosopumilus sp. HCA1 (KF957663.1), *Nitrosopumilus maritimus* strain SCM1 (NR_102913.1)	90%	0	1	0	0	0
**34421**	LT897033	*Candidatus* Nitrosopelagicus brevis strain CN25 (CP007026.1)	99%	0	990	0	756	36
**34454**	LT897034	*Candidatus* Nitrosopelagicus brevis strain CN25 (CP007026.1)	98%	0	10124	0	202	44
**34463**	LT897035	*Candidatus* Nitrosopelagicus brevis strain CN25 (CP007026.1)	94%	0	2	0	1	1
**34491**	LT897036	*Candidatus* Nitrosopumilus sp. HCA1 (KF957663.1), *Nitrosopumilus maritimus* strain SCM1 (NR_102913.1)	100%	2	25408	3	12629	1247
**34499**	LT897037	*Candidatus* Nitrosopelagicus brevis strain CN25 (CP007026.1)	98%	0	295	0	18	126

**34504**	LT897038	*Candidatus* Nitrosopelagicus brevis strain CN25 (CP007026.1), *Nitrosopumilus* sp. DDS1 (KR737579.1)	96%	0	75	0	374	2421
**34511**	LT897039	*Nitrosopumilus* sp. DDS1 (KR737579.1), *Candidatus* Nitrosopumilus sp. NF5 (CP011070.1)	98%	0	259	0	22	6
**34526**	LT897040	*Candidatus* Nitrosopumilus sp. HCA1 (KF957663.1), *Nitrosopumilus maritimus* strain SCM1 (NR_102913.1)	98%	0	2222	0	106	51
**34531**	LT897041	*Candidatus* Nitrosopelagicus brevis strain CN25 (CP007026.1)	94%	0	2	0	0	1
**34557**	LT897042	*Candidatus* Nitrosopumilus sp. HCA1 (KF957663.1), *Nitrosopumilus maritimus* strain SCM1 (NR_102913.1)	96%	0	1	0	2	0
**34580**	LT897043	*Candidatus* Nitrosopelagicus brevis strain CN25 (CP007026.1)	95%	0	2	0	4	6
**34582**	LT897044	*Candidatus* Nitrosopelagicus brevis strain CN25 (CP007026.1)	91%	0	1	0	0	0
**34588**	LT897045	*Candidatus* Nitrosopelagicus brevis strain CN25 (CP007026.1)	96%	0	934	0	107	24
**34609**	LT897046	*Nitrosopumilus* sp. DDS1 (KR737579.1), *Candidatus* Nitrosopumilus sp. NF5 (CP011070.1)	95%	0	2	0	12	199
**34619**	LT897047	*Candidatus* Nitrosopelagicus brevis strain CN25 (CP007026.1), *Nitrosopumilus* sp. DDS1 (KR737579.1)	96%	0	39	0	140	185
**34686**	LT897048	*Nitrosopumilus* sp. DDS1 (KR737579.1), *Candidatus* Nitrosopumilus sp. NF5 (CP011070.1)	93%	0	1	0	0	0
**34693**	LT897049	*Candidatus* Nitrosopelagicus brevis strain CN25 (CP007026.1)	89%	0	1	0	0	0
**34745**	LT897050	*Candidatus* Nitrosopumilus sp. HCA1 (KF957663.1)	99%	0	536	0	93	2
**34746**	LT897051	*Candidatus* Nitrosopumilus sp. HCA1 (KF957663.1)	93%	0	1	0	0	0
**34809**	LT897052	*Candidatus* Nitrosopelagicus brevis strain CN25 (CP007026.1)	89%	0	1	0	0	0
**34826**	LT897053	*Candidatus* Nitrosopelagicus brevis strain CN25 (CP007026.1)	95%	0	829	0	443	14
**34849**	LT897054	*Candidatus* Nitrosopelagicus brevis strain CN25 (CP007026.1)	95%	0	5	0	30	3
**34851**	LT897055	*Candidatus* Nitrosopumilus sp. HCA1(KF957663.1)	98%	0	1615	0	1110	62
**34856**	LT897056	*Candidatus* Nitrosopelagicus brevis strain CN25 (CP007026.1)	97%	0	325	0	544	24
**34861**	LT897057	*Candidatus* Nitrosopelagicus brevis strain CN25 (CP007026.1)	99%	0	1	0	0	0
**34869**	LT897058	*Candidatus* Nitrosopumilus sp. HCA1(KF957663.1)	94%	0	2	0	5	7
**34883**	LT897059	*Candidatus* Nitrosopelagicus brevis strain CN25 (CP007026.1)	95%	0	1	0	8	1
**34886**	LT897060	*Candidatus* Nitrosopumilus sp. HCA1(KF957663.1)	95%	0	1	0	0	0

**34891**	LT897061	*Candidatus* Nitrosopumilus sp. NF5 (CP011070.1), *Candidatus* Nitrosopumilus sp. D3C (CP010868.1), *Candidatus* Nitrosopelagicus brevis strain CN25 (CP007026.1)	98%	0	191	0	14	0
**34893**	LT897062	*Candidatus* Nitrosopelagicus brevis strain CN25 (CP007026.1)	96%	0	33	0	136	74
**34895**	LT897063	*Candidatus* Nitrosopelagicus brevis strain CN25 (CP007026.1)	96%	0	160	0	36	36
**34896**	LT897064	*Candidatus* Nitrosopumilus sp. HCA1(KF957663.1)	92%	0	1	0	1	0
**34897**	LT897065	*Candidatus* Nitrosopumilus sp. HCA1(KF957663.1)	100%	3	1673 9	1	2911	111
**34915**	LT897066	*Candidatus* Nitrosopumilus sp. HCA1(KF957663.1)	99%	0	47	0	174	8
**34940**	LT897067	*Candidatus* Nitrosopelagicus brevis strain CN25 (CP007026.1)	96%	0	1	0	0	3
**34951**	LT897068	*Candidatus* Nitrosopelagicus brevis strain CN25 (CP007026.1)	98%	0	105	0	468	7
**34977**	LT897069	*Candidatus* Nitrosopelagicus brevis strain CN25 (CP007026.1)	95%	0	2	0	6	0
**35024**	LT897070	*Candidatus* Nitrosopumilus sp. NF5 (CP011070.1)	98%	0	60	0	183	36
**35031**	LT897071	*Candidatus* Nitrosopelagicus brevis strain CN25 (CP007026.1)	95%	0	189	0	280	9
**35034**	LT897072	*Candidatus* Nitrosopelagicus brevis strain CN25 (CP007026.1)	98%	0	6101	0	4231	26
**35058**	LT897073	*Candidatus* Nitrosopelagicus brevis strain CN25 (CP007026.1)	95%	0	1	0	5	33
**35112**	LT897074	*Candidatus* Nitrosopumilus sp. HCA1 (KF957663.1)	98%	0	1146	0	808	4
**35123**	LT897075	*Candidatus* Nitrosopelagicus brevis strain CN25 (CP007026.1)	96%	0	47	0	110	1
**35144**	LT897076	*Candidatus* Nitrosopumilus sp. HCA1 (KF957663.1)	93%	0	1	0	1	1
**35170**	LT897077	*Candidatus* Nitrosopelagicus brevis strain CN25 (CP007026.1)	97%	0	1	0	3	0
**35241**	LT897078	*Candidatus* Nitrosopelagicus brevis strain CN25 (CP007026.1)	95%	0	1	0	2078	25
**35249**	LT897079	*Candidatus* Nitrosopelagicus brevis strain CN25 (CP007026.1)	98%	0	4	0	11588	162
**35292**	LT897080	*Candidatus* Nitrosopumilus sp. NF5 (CP011070.1)	94%	0	1	0	0	1
**35293**	LT897081	*Candidatus* Nitrosopumilus sp. NF5 (CP011070.1), *Candidatus* Nitrosopumilus sp. D3C (CP010868.1)	94%	0	2	0	4	68
**35295**	LT897082	*Candidatus* Nitrosopumilus sp. HCA1 (KF957663.1),	99%	0	1	0	45	2
**35335**	LT897083	*Candidatus* Nitrosopumilus sp. HCA1 (KF957663.1)	93%	0	1	0	1	0
**35349**	LT897084	*Candidatus* Nitrosopumilus sp. HCA1 (KF957663.1)	93%	0	1	0	27	12
**35369**	LT897085	*Candidatus* Nitrosopelagicus brevis strain CN25 (CP007026.1)	97%	0	0	0	301	6

**35375**	LT897086	*Candidatus* Nitrosopelagicus brevis strain CN25 (CP007026.1)	96%	0	0	0	17	24
**35420**	LT897087	*Candidatus* Nitrosopumilus sp. HCA1 (KF957663.1)	97%	0	0	0	18	1
**35443**	LT897088	*Candidatus* Nitrosopumilus sp. HCA1 (KF957663.1)	98%	0	0	0	2949	285
**35447**	LT897089	*Candidatus* Nitrosopelagicus brevis strain CN25 (CP007026.1)	96%	0	0	0	3	0
**35457**	LT897090	*Candidatus* Nitrosopelagicus brevis strain CN25 (CP007026.1)	89%	0	0	0	1	0
**35483**	LT897091	*Candidatus* Nitrosopumilus sp. HCA1 (KF957663.1)	100%	0	5	0	17633	134
**35511**	LT897092	*Nitrosopumilus* sp. DDS1 (KR737579.1)	90%	0	0	0	1	4
**35512**	LT897093	*Candidatus* Nitrosopelagicus brevis strain CN25 (CP007026.1)	93%	0	0	0	1	1
**35516**	LT897094	*Candidatus* Nitrosopelagicus brevis strain CN25 (CP007026.1)	96%	0	0	0	54	50
**35597**	LT897095	*Candidatus* Nitrosopelagicus brevis strain CN25 (CP007026.1)	95%	0	0	0	489	22
**35619**	LT897096	*Nitrosopumilus* sp. DDS1 (KR737579.1)	98%	0	0	0	647	224
**35670**	LT897097	*Nitrosopumilus* sp. DDS1 (KR737579.1), *Candidatus* Nitrosopumilus sp. NF5 (CP011070.1), *Candidatus* Nitrosopumilus sp. D3C (CP010868.1)	96%	0	0	0	9	51
**35737**	LT897098	*Nitrosopumilus maritimus* strain NAOA6 (KT380502.1)	99%	0	0	0	113	7
**35757**	LT897099	*Nitrosopumilus* sp. DDS1 (KR737579.1), *Candidatus* Nitrosopumilus sp. NF5 (CP011070.1), *Candidatus* Nitrosopumilus sp. D3C (CP010868.1), *Candidatus* Nitrosopelagicus brevis strain CN25 (CP007026.1)	98%	0	0	0	720	107
**35762**	LT897100	*Candidatus* Nitrosopelagicus brevis strain CN25 (CP007026.1)	97%	0	0	0	1310	20
**35766**	LT897101	*Candidatus* Nitrosopelagicus brevis strain CN25 (CP007026.1)	97%	0	1	0	187	6
**35827**	LT897102	*Candidatus* Nitrosopelagicus brevis strain CN25 (CP007026.1)	97%	0	0	0	3	0
**35832**	LT897103	*Candidatus* Nitrosopelagicus brevis strain CN25 (CP007026.1)	97%	1	1	0	17	7
**35884**	LT897104	*Candidatus* Nitrosopelagicus brevis strain CN25 (CP007026.1)	95%	0	1	0	3	1
**35893**	LT897105	*Candidatus* Nitrosopumilus sp. HCA1 (KF957663.1), *Nitrosopumilus maritimus* strain NAOA6 (KT380502.1)	92%	0	0	0	1	0
**35909**	LT897106	*Candidatus* Nitrosopelagicus brevis strain CN25 (CP007026.1)	95%	0	0	0	53	22
**35925**	LT897107	*Candidatus* Nitrosopelagicus brevis strain CN25 (CP007026.1)	93%	0	0	0	1	0
**35951**	LT897108	*Candidatus* Nitrosopelagicus brevis strain CN25 (CP007026.1)	95%	0	0	0	71	65

**35966**	LT897109	*Nitrosopumilus* sp. DDS1 (KR737579.1), *Candidatus* Nitrosopumilus sp. NF5 (CP011070.1), *Candidatus* Nitrosopumilus sp. D3C (CP010868.1), *Candidatus* Nitrosopelagicus brevis strain CN25 (CP007026.1)	97%	0	0	0	415	537
**36007**	LT897110	*Nitrosopumilus maritimus* strain NAOA6 (KT380502.1), *Candidatus* Nitrosopumilus sp. HCA1 (KF957663.1)	99%	0	0	0	102	22
**36011**	LT897111	*Nitrosopumilus* sp. DDS1 (KR737579.1), *Candidatus* Nitrosopumilus sp. NF5 (CP011070.1), *Candidatus* Nitrosopumilus sp. D3C (CP010868.1), *Candidatus* Nitrosopelagicus brevis strain CN25 (CP007026.1)	95%	0	0	0	5	2
**36019**	LT897112	*Nitrosopumilus* sp. DDS1 (KR737579.1), *Candidatus* Nitrosopumilus sp. NF5 (CP011070.1), *Candidatus* Nitrosopumilus sp. D3C (CP010868.1), *Candidatus* Nitrosopelagicus brevis strain CN25 (CP007026.1)	97%	0	3	0	543	35
**36032**	LT897113	*Candidatus* Nitrosopelagicus brevis strain CN25 (CP007026.1)	97%	0	0	0	235	21
**36041**	LT897114	*Candidatus* Nitrosopumilus sp. HCA1 (KF957663.1), *Nitrosopumilus maritimus* strain NAOA6 (KT380502.1)	93%	0	0	0	1	0
**36124**	LT897115	*Candidatus* Nitrosopelagicus brevis strain CN25 (CP007026.1)	91%	0	0	0	1	0
**36171**	LT897116	*Nitrosopumilus* sp. DDS1 (KR737579.1), *Candidatus* Nitrosopumilus sp. NF5 (CP011070.1), *Candidatus* Nitrosopumilus sp. D3C (CP010868.1), *Candidatus* Nitrosopelagicus brevis strain CN25 (CP007026.1)	96%	0	0	0	77	31
**36180**	LT897117	*Nitrosopumilus maritimus* strain NAOA6 (KT380502.1)	99%	0	0	0	174	170
**36229**	LT897118	*Candidatus* Nitrosopelagicus brevis strain CN25 (CP007026.1)	95%	0	1	0	139	59
**36238**	LT897119	*Candidatus* Nitrosopelagicus brevis strain CN25 (CP007026.1)	97%	0	1	0	5	0
**36241**	LT897120	*Candidatus* Nitrosopelagicus brevis strain CN25 (CP007026.1)	93%	0	0	0	1	0
**36298**	LT897121	*Nitrosopumilus* sp. DDS1 (KR737579.1), *Candidatus* Nitrosopumilus sp. NF5 (CP011070.1), *Candidatus* Nitrosopumilus sp. D3C (CP010868.1), *Candidatus* Nitrosopelagicus brevis strain CN25 (CP007026.1)	92%	0	0	0	1	0
**36299**	LT897122	*Candidatus* Nitrosopelagicus brevis strain CN25 (CP007026.1)	95%	0	0	0	851	1192
**36306**	LT897123	*Candidatus* Nitrosopumilus sp. HCA1 (KF957663.1), *Nitrosopumilus maritimus* strain NAOA6 (KT380502.1)	92%	0	0	0	1	0

**36322**	LT897124	*Nitrosopumilus* sp. DDS1 (KR737579.1), *Candidatus* Nitrosopumilus sp. NF5 (CP011070.1), *Candidatus* Nitrosopumilus sp. D3C (CP010868.1), *Candidatus* Nitrosopelagicus brevis strain CN25 (CP007026.1)	96%	0	0	0	25	50
**36328**	LT897125	*Candidatus* Nitrosopelagicus brevis strain CN25 (CP007026.1)	96%	0	0	0	3	7
**36352**	LT897126	*Candidatus* Nitrosopelagicus brevis strain CN25 (CP007026.1)	95%	0	0	1	58	78
**36357**	LT897127	*Candidatus* Nitrosopelagicus brevis strain CN25 (CP007026.1)	90%	0	0	0	1	2
**36361**	LT897128	*Candidatus* Nitrosopelagicus brevis strain CN25 (CP007026.1)	95%	0	1	0	25	28
**36369**	LT897129	*Candidatus* Nitrosopelagicus brevis strain CN25 (CP007026.1)	98%	0	1	0	11993	36
**36420**	LT897130	*Candidatus* Nitrosopelagicus brevis strain CN25 (CP007026.1)	90%	0	1	0	1	0
**36448**	LT897131	*Candidatus* Nitrosopelagicus brevis strain CN25 (CP007026.1)	96%	0	0	0	19	8
**36508**	LT897132	*Candidatus* Nitrosopelagicus brevis strain CN25 (CP007026.1)	91%	0	0	0	1	0
**36525**	LT897133	*Candidatus* Nitrosopelagicus brevis strain CN25 (CP007026.1)	96%	0	0	0	34	31
**36530**	LT897134	*Candidatus* Nitrosopumilus sp. NF5 (CP011070.1)	96%	0	0	0	1	0
**36561**	LT897135	*Candidatus* Nitrosopelagicus brevis strain CN25 (CP007026.1)	96%	0	0	0	838	189
**36585**	LT897136	*Nitrosopumilus maritimus* strain NAOA6 (KT380502.1)	98%	0	0	0	6205	357
**36587**	LT897137	*Candidatus* Nitrosopelagicus brevis strain CN25 (CP007026.1)	94%	0	1	0	241	146
**36591**	LT897138	*Candidatus* Nitrosopelagicus brevis strain CN25 (CP007026.1)	96%	0	0	0	578	9
**36598**	LT897139	*Candidatus* Nitrosopelagicus brevis strain CN25 (CP007026.1)	91%	0	0	0	1	0
**36626**	LT897140	*Candidatus* Nitrosopelagicus brevis strain CN25 (CP007026.1)	99%	0	0	0	237	10
**36640**	LT897141	*Candidatus* Nitrosopelagicus brevis strain CN25 (CP007026.1)	95%	0	0	0	2337	550
**36708**	LT897142	*Nitrosopumilus maritimus* strain NAOA6 (KT380502.1)	98%	0	10	1	4015	659
**36720**	LT897143	*Candidatus* Nitrosopelagicus brevis strain CN25 (CP007026.1)	95%	0	1	0	19	10
**36721**	LT897144	*Candidatus* Nitrosopelagicus brevis strain CN25 (CP007026.1)	95%	0	0	0	1029	94
**36761**	LT897145	*Nitrosopumilus* sp. DDS1 (KR737579.1)	95%	0	0	0	3	126
**36832**	LT897146	*Nitrosopumilus* sp. DDS1 (KR737579.1)	96%	0	0	0	433	4861
**36839**	LT897147	*Candidatus* Nitrosopumilus sp. AR2 (CP003843.1), *Candidatus* Nitrosopumilus koreensis AR1 (CP003842.1	94%	0	0	0	24	405
**36858**	LT897148	*Nitrosopumilus maritimus* strain NAOA6 (KT380502.1), *Nitrosopumilus* sp. DDS1 (KR737579.1)	98%	0	0	0	111	7

**36861**	LT897149	*Candidatus* Nitrosopelagicus brevis strain CN25 (CP007026.1)	96%	0	16	1	394	427
**36872**	LT897150	*Nitrosopumilus* sp. DDS1 (KR737579.1)	96%	0	0	0	42	38
**36894**	LT897151	*Nitrosopumilus* sp. DDS1 (KR737579.1)	96%	0	1	0	4009	28
**36903**	LT897152	*Candidatus* Nitrosopelagicus brevis strain CN25 (CP007026.1)	96%	0	0	0	77	247
**36952**	LT897153	*Candidatus* Nitrosopelagicus brevis strain CN25 (CP007026.1)	99%	0	3	0	123	3
**36955**	LT897154	*Nitrosopumilus* sp. DDS1 (KR737579.1)	98%	0	0	0	536	18
**36969**	LT897155	*Candidatus* Nitrosopelagicus brevis strain CN25 (CP007026.1)	94%	0	0	0	12	10
**36985**	LT897156	*Candidatus* Nitrosopelagicus brevis strain CN25 (CP007026.1)	89%	0	0	0	1	0
**36990**	LT897157	*Candidatus* Nitrosopelagicus brevis strain CN25 (CP007026.1)	92%	0	0	0	1	4
**36998**	LT897158	*Nitrosopumilus* sp. DDS1 (KR737579.1)	94%	0	0	0	1	2
**37032**	LT897159	*Nitrosopumilus* sp. DDS1 (KR737579.1)	96%	0	0	0	69	486
**37036**	LT897160	*Nitrosopumilus* sp. DDS1 (KR737579.1)	96%	0	0	0	5	4
**37119**	LT897161	*Nitrosopumilus* sp. DDS1 (KR737579.1)	96%	0	0	0	31	31
**37142**	LT897162	*Candidatus* Nitrosopelagicus brevis strain CN25 (CP007026.1)	93%	0	0	0	1	0
**37147**	LT897163	*Candidatus* Nitrosopelagicus brevis strain CN25 (CP007026.1)	99%	0	0	0	51	8
**37164**	LT897164	*Candidatus* Nitrosopelagicus brevis strain CN25 (CP007026.1)	99%	0	1	0	74	2
**37168**	LT897165	*Nitrosopumilus maritimus* strain SCM1 (NR_102913.1), *Candidatus* Nitrosopumilus koreensis strain AR1 (NR_102904.1), *Candidatus* Nitrosopumilus sp. AR2 (CP003843.1), *Candidatus* Nitrosopumilus koreensis AR1 (CP003842.1), *Nitrosopumilus maritimus* SCM1 (CP000866.1)	97%	0	0	0	12	23
**37188**	LT897166	*Candidatus* Nitrosopelagicus brevis strain CN25 (CP007026.1)	96%	0	1	0	780	165
**37203**	LT897167	*Candidatus* Nitrosopelagicus brevis strain CN25 (CP007026.1)	95%	0	0	0	1699	521
**37333**	LT897168	*Nitrosopumilus* sp. DDS1 (KR737579.1), *Candidatus* Nitrosopumilus sp. NF5 (CP011070.1), *Candidatus* Nitrosopumilus sp. D3C (CP010868.1), *Candidatus* Nitrosopelagicus brevis strain CN25 (CP007026.1)	98%	0	0	0	460	46
**37347**	LT897169	*Nitrosopumilus maritimus* strain SCM1 (NR_102913.1), *Candidatus* Nitrosopumilus koreensis strain AR1 (NR_102904.1), *Candidatus* Nitrosopumilus sp. AR2 (CP003843.1), *Candidatus* Nitrosopumilus koreensis AR1 (CP003842.1), *Nitrosopumilus maritimus* SCM1 (CP000866.1)	100%	0	8	0	8934	115
**37398**	LT897170	*Candidatus* Nitrosopelagicus brevis strain CN25 (CP007026.1)	95%	0	0	0	267	126

**37442**	LT897171	*Nitrosopumilus* sp. DDS1 (KR737579.1), *Candidatus* Nitrosopumilus sp. NF5 (CP011070.1), *Candidatus* Nitrosopumilus sp. D3C (CP010868.1), *Candidatus* Nitrosopelagicus brevis strain CN25 (CP007026.1)	95%	0	1	0	90	144
**37459**	LT897172	*Candidatus* Nitrosopelagicus brevis strain CN25 (CP007026.1)	100%	0	5	0	1104	5
**37473**	LT897173	*Nitrosopumilus maritimus* strain SCM1 (NR_102913.1), *Candidatus* Nitrosopumilus koreensis strain AR1 (NR_102904.1), *Candidatus* Nitrosopumilus sp. AR2 (CP003843.1), *Candidatus* Nitrosopumilus koreensis AR1 (CP003842.1), *Nitrosopumilus maritimus* SCM1 (CP000866.1)	94%	0	0	0	1	1
**37475**	LT897174	*Candidatus* Nitrosopelagicus brevis strain CN25 (CP007026.1)	98%	0	0	0	7223	23
**37489**	LT897175	*Candidatus* Nitrosopelagicus brevis strain CN25 (CP007026.1)	96%	0	0	0	102	26
**37496**	LT897176	*Candidatus* Nitrosopelagicus brevis strain CN25 (CP007026.1)	95%	0	0	0	528	37
**37518**	LT897177	*Candidatus* Nitrosopelagicus brevis strain CN25 (CP007026.1)	88%	0	0	0	2	1
**37554**	LT897178	*Candidatus* Nitrosopelagicus brevis strain CN25 (CP007026.1)	96%	0	0	0	25	3
**37617**	LT897179	*Nitrosopumilus* sp. DDS1 (KR737579.1), *Candidatus* Nitrosopumilus sp. NF5 (CP011070.1), *Candidatus* Nitrosopumilus sp. D3C (CP010868.1), *Candidatus* Nitrosopelagicus brevis strain CN25 (CP007026.1)	96%	0	1	0	52	1174
**37770**	LT897180	*Nitrosopumilus* sp. DDS1 (KR737579.1), *Candidatus* Nitrosopumilus sp. NF5 (CP011070.1), *Candidatus* Nitrosopumilus sp. D3C (CP010868.1), *Candidatus* Nitrosopelagicus brevis strain CN25 (CP007026.1)	96%	0	0	0	4	0
**37778**	LT897181	*Candidatus* Nitrosopelagicus brevis strain CN25 (CP007026.1)	96%	0	0	0	23	330
**37807**	LT897182	*Nitrosopumilus maritimus* strain SCM1 (NR_102913.1), *Candidatus* Nitrosopumilus koreensisstrain AR1 (NR_102904.1), *Candidatus* Nitrosopumilus sp. AR2 (CP003843.1), *Candidatus* Nitrosopumilus koreensis AR1 (CP003842.1), *Nitrosopumilus maritimus* SCM1 (CP000866.1)	99%	0	5	1	48	42
**37827**	LT897183	*Candidatus* Nitrosopelagicus brevis strain CN25 (CP007026.1), *Nitrosopumilus* sp. DDS1 (KR737579.1), *Candidatus* Nitrosopumilus sp. NF5 (CP011070.1), *Candidatus* Nitrosopumilus sp. D3C (CP010868.1)	94%	0	0	0	30	71

**37846**	LT897184	*Candidatus* Nitrosopelagicus brevis strain CN25 (CP007026.1)	96%	0	1	0	30	47
**37875**	LT897185	*Candidatus* Nitrosopelagicus brevis strain CN25 (CP007026.1)	97%	0	0	0	118	81
**37902**	LT897186	*Candidatus* Nitrosopelagicus brevis strain CN25 (CP007026.1)	97%	0	2	0	267	22
**37964**	LT897187	*Nitrosopumilus* sp. DDS1 (KR737579.1), *Candidatus* Nitrosopumilus sp. NF5 (CP011070.1), *Candidatus* Nitrosopumilus sp. D3C (CP010868.1), *Candidatus* Nitrosopelagicus brevis strain CN25 (CP007026.1)	97%	0	0	0	95	38
**37965**	LT897188	*Candidatus* Nitrosopelagicus brevis strain CN25 (CP007026.1)	100%	0	0	0	1228	24
**37998**	LT897189	*Nitrosopumilus maritimus* strain SCM1 (NR_102913.1), *Candidatus* Nitrosopumilus koreensis strain AR1 (NR_102904.1), *Candidatus* Nitrosopumilus sp. AR2 (CP003843.1), *Nitrosopumilus maritimus* strain NAOA6 (KT380502.1), *Candidatus* Nitrosopumilus sp. HCA1 (KF957663.1)	99%%	0	0	0	23	12
**38000**	LT897190	*Nitrosopumilus* sp. DDS1 (KR737579.1), *Candidatus* Nitrosopumilus sp. NF5 (CP011070.1), *Candidatus* Nitrosopumilus sp. D3C (CP010868.1), *Candidatus* Nitrosopelagicus brevis strain CN25 (CP007026.1)	97%	0	1	0	9	19
**38009**	LT897191	*Nitrosopumilus* sp. DDS1 (KR737579.1), *Candidatus* Nitrosopumilus sp. NF5 (CP011070.1), *Candidatus* Nitrosopumilus sp. D3C (CP010868.1), *Candidatus* Nitrosopelagicus brevis strain CN25 (CP007026.1)	89%	0	0	0	2	1
**38037**	LT897192	*Candidatus* Nitrosopelagicus brevis strain CN25 (CP007026.1)	93%	0	0	0	1	0
**38051**	LT897193	*Candidatus* Nitrosopelagicus brevis strain CN25 (CP007026.1)	98%	0	0	0	1	0
**38059**	LT897194	*Nitrosopumilus maritimus* strain NAOA6 (KT380502.1), *Candidatus* Nitrosopumilus sp. HCA1 (KF957663.1)	98%	0	11	1	1316	411
**38064**	LT897195	*Candidatus* Nitrosopelagicus brevis strain CN25 (CP007026.1)	94%	0	0	0	1	1
**38101**	LT897196	*Candidatus* Nitrosopelagicus brevis strain CN25 (CP007026.1)	99%	0	1	0	109	73
**38115**	LT897197	*Candidatus* Nitrosopelagicus brevis strain CN25 (CP007026.1)	96%	0	0	0	3	30
**38137**	LT897198	*Nitrosopumilus* sp. DDS1 (KR737579.1), *Candidatus* Nitrosopumilus sp. NF5 (CP011070.1), *Candidatus* Nitrosopumilus sp. D3C (CP010868.1), *Candidatus* Nitrosopelagicus brevis strain CN25 (CP007026.1)	96%	0	1	0	15	59

**38152**	LT897199	*Nitrosopumilus* sp. DDS1 (KR737579.1), *Candidatus* Nitrosopumilus sp. NF5 (CP011070.1), *Candidatus* Nitrosopumilus sp. D3C (CP010868.1), *Candidatus* Nitrosopelagicus brevis strain CN25 (CP007026.1)	92%	0	0	0	221	14
**38166**	LT897200	*Nitrosopumilus* sp. DDS1 (KR737579.1), *Candidatus* Nitrosopumilus sp. NF5 (CP011070.1), *Candidatus* Nitrosopumilus sp. D3C (CP010868.1), *Candidatus* Nitrosopelagicus brevis strain CN25 (CP007026.1)	96%	0	1	0	44	43
**38232**	LT897201	*Candidatus* Nitrosopelagicus brevis strain CN25 (CP007026.1)	94%	0	0	0	3	2
**38249**	LT897202	*Candidatus* Nitrosopelagicus brevis strain CN25 (CP007026.1)	94%	0	0	0	9	845
**38261**	LT897203	*Nitrosopumilus maritimus* strain NAOA6 (KT380502.1), *Candidatus* Nitrosopumilus sp. HCA1 (KF957663.1)	95%	0	0	0	1	0
**38320**	LT897204	*Candidatus* Nitrosopelagicus brevis strain CN25 (CP007026.1)	98%	0	0	0	10331	450
**38321**	LT897205	*Nitrosopumilus* sp. DDS1 (KR737579.1), *Candidatus* Nitrosopumilus sp. NF5 (CP011070.1), *Candidatus* Nitrosopumilus sp. D3C (CP010868.1), *Candidatus* Nitrosopelagicus brevis strain CN25 (CP007026.1)	98%	0	0	0	1	2
**38363**	LT897206	*Nitrosopumilus maritimus* strain SCM1 (NR_102913.1), *Candidatus* Nitrosopumilus koreensis strain AR1 (NR_102904.1), *Candidatus* Nitrosopumilus sp. AR2 (CP003843.1), *Nitrosopumilus maritimus* strain NAOA6 (KT380502.1), *Candidatus* Nitrosopumilus sp. HCA1 (KF957663.1)	100%	0	17	0	10418	7179
**38387**	LT897207	*Candidatus* Nitrosopelagicus brevis strain CN25 (CP007026.1)	97%	0	3	2	555	581
**38399**	LT897208	*Nitrosopumilus* sp. DDS1 (KR737579.1), *Candidatus* Nitrosopumilus sp. NF5 (CP011070.1), *Candidatus* Nitrosopumilus sp. D3C (CP010868.1)	95%	0	0	0	10	23
**38439**	LT897209	*Candidatus* Nitrosopelagicus brevis strain CN25 (CP007026.1)	95%	0	0	0	2	647
**38478**	LT897210	*Candidatus* Nitrosopelagicus brevis strain CN25 (CP007026.1)	96%	0	1	0	1	18
**38487**	LT897211	*Candidatus* Nitrosopelagicus brevis strain CN25 (CP007026.1)	96%	0	0	0	2	0
**38509**	LT897212	*Candidatus* Nitrosopelagicus brevis strain CN25 (CP007026.1)	96%	0	1	0	1	15
**38510**	LT897213	*Nitrosopumilus* sp. DDS1 (KR737579.1), *Candidatus* Nitrosopumilus sp. NF5 (CP011070.1), *Candidatus* Nitrosopumilus sp. D3C (CP010868.1)	98%	0	6	0	1	1251
**38523**	LT897214	*Candidatus* Nitrosopelagicus brevis strain CN25 (CP007026.1)	91%	0	0	0	1	0

**38525**	LT897215	*Candidatus* Nitrosopelagicus brevis strain CN25 (CP007026.1)	96%	0	17	2	10	422
**38641**	LT897216	*Nitrosopumilus maritimus* strain SCM1 (NR_102913.1), *Candidatus* Nitrosopumilus koreensis strain AR1 (NR_102904.1), *Candidatus* Nitrosopumilus sp. AR2 (CP003843.1), *Candidatus* Nitrosopumilus koreensis AR1 (CP003842.1), *Nitrosopumilus maritimus* SCM1 (CP000866.1)	100%	0	5	0	3	10890
**38661**	LT897217	*Candidatus* Nitrosopelagicus brevis strain CN25 (CP007026.1)	97%	0	10	1	12	354
**38670**	LT897218	*Nitrosopumilus* sp. DDS1 (KR737579.1), *Candidatus* Nitrosopumilus sp. NF5 (CP011070.1), *Candidatus* Nitrosopumilus sp. D3C (CP010868.1)	95%	0	0	0	1	0
**38674**	LT897219	*Nitrosopumilus maritimus* strain SCM1 (NR_102913.1), *Candidatus* Nitrosopumilus koreensis strain AR1 (NR_102904.1), *Candidatus* Nitrosopumilus sp. AR2 (CP003843.1), *Candidatus* Nitrosopumilus koreensis AR1 (CP003842.1), *Nitrosopumilus maritimus* SCM1 (CP000866.1)	98%	0	2	0	2	1390
**38681**	LT897220	*Candidatus* Nitrosopelagicus brevis strain CN25 (CP007026.1)	98%	1	11	1	12	105
**38700**	LT897221	*Candidatus* Nitrosopelagicus brevis strain CN25 (CP007026.1)	98%	0	1	1	4	3431
**38741**	LT897222	*Candidatus* Nitrosopelagicus brevis strain CN25 (CP007026.1)	95%	0	1	0	6	759
**38751**	LT897223	*Nitrosopumilus maritimus* strain SCM1 (NR_102913.1), *Candidatus* Nitrosopumilus koreensis strain AR1 (NR_102904.1), *Candidatus* Nitrosopumilus sp. AR2 (CP003843.1), *Candidatus* Nitrosopumilus koreensis AR1 (CP003842.1), *Nitrosopumilus maritimus* SCM1 (CP000866.1)	99%	0	0	0	1	3
**38755**	LT897224	*Nitrosopumilus maritimus* strain SCM1 (NR_102913.1), *Candidatus* Nitrosopumilus koreensis strain AR1 (NR_102904.1), *Candidatus* Nitrosopumilus sp. AR2 (CP003843.1), *Candidatus* Nitrosopumilus koreensis AR1 (CP003842.1), *Nitrosopumilus maritimus* SCM1 (CP000866.1)	98%	0	2	0	1	3459
**38811**	LT897225	*Candidatus* Nitrosopelagicus brevis strain CN25 (CP007026.1)	100%	0	5	0	8	874
**38827**	LT897226	*Candidatus* Nitrosopelagicus brevis strain CN25 (CP007026.1)	96%	0	0	0	1	184

**38880**	LT897227	*Nitrosopumilus maritimus* strain SCM1 (NR_102913.1), *Candidatus* Nitrosopumilus koreensis strain AR1 (NR_102904.1), *Candidatus* Nitrosopumilus sp. AR2 (CP003843.1), *Candidatus* Nitrosopumilus koreensis AR1 (CP003842.1), *Nitrosopumilus maritimus* SCM1 (CP000866.1)	95%	1	0	0	1	1
**38888**	LT897228	*Candidatus* Nitrosopelagicus brevis strain CN25 (CP007026.1)	95%	0	1	0	1	991
**38938**	LT897229	*Nitrosopumilus maritimus* strain SCM1 (NR_102913.1), *Candidatus* Nitrosopumilus koreensis strain AR1 (NR_102904.1), *Candidatus* Nitrosopumilus sp. AR2 (CP003843.1), *Candidatus* Nitrosopumilus koreensis AR1 (CP003842.1), *Nitrosopumilus maritimus* SCM1 (CP000866.1)	98%	0	1	0	3	45
**38948**	LT897230	*Nitrosopumilus maritimus* strain SCM1 (NR_102913.1), *Candidatus* Nitrosopumilus koreensis strain AR1 (NR_102904.1), *Candidatus* Nitrosopumilus sp. AR2 (CP003843.1), *Candidatus* Nitrosopumilus koreensis AR1 (CP003842.1), *Nitrosopumilus maritimus* SCM1 (CP000866.1)	99%	0	0	0	1	38
**38958**	LT897231	*Candidatus* Nitrosopelagicus brevis strain CN25 (CP007026.1)	90%	0	0	0	1	0
**38981**	LT897232	*Candidatus* Nitrosopelagicus brevis strain CN25 (CP007026.1), *Nitrosopumilus* sp. DDS1 (KR737579.1), *Candidatus* Nitrosopumilus sp. NF5 (CP011070.1), *Candidatus* Nitrosopumilus sp. D3C (CP010868.1)	94%	0	0	0	1	0
**38997**	LT897233	*Candidatus* Nitrosopelagicus brevis strain CN25 (CP007026.1)	97%	0	0	0	1	83
**38998**	LT897234	*Nitrosopumilus* sp. DDS1 (KR737579.1), *Candidatus* Nitrosopumilus sp. NF5 (CP011070.1), *Candidatus* Nitrosopumilus sp. D3C (CP010868.1)	98%	0	7	0	2	68
**39088**	LT897235	*Candidatus* Nitrosopelagicus brevis strain CN25 (CP007026.1)	94%	0	0	0	1	1
**39146**	LT897236	*Candidatus* Nitrosopelagicus brevis strain CN25 (CP007026.1)	95%	0	0	0	1	5
**39149**	LT897237	*Candidatus* Nitrosopelagicus brevis strain CN25 (CP007026.1)	93%	0	0	0	1	2
**39174**	LT897238	*Nitrosopumilus* sp. DDS1 (KR737579.1), *Candidatus* Nitrosopumilus sp. NF5 (CP011070.1), *Candidatus* Nitrosopumilus sp. D3C (CP010868.1)	93%	0	0	0	0	7

**39209**	LT897239	*Candidatus* Nitrosopelagicus brevis strain CN25 (CP007026.1), *Nitrosopumilus* sp. DDS1 (KR737579.1), *Candidatus* Nitrosopumilus sp. NF5 (CP011070.1), *Candidatus* Nitrosopumilus sp. D3C (CP010868.1)	96%	0	0	0	0	650
**39214**	LT897240	*Candidatus* Nitrosopelagicus brevis strain CN25 (CP007026.1)	96%	0	0	0	0	60
**39220**	LT897241	*Candidatus* Nitrosopelagicus brevis strain CN25 (CP007026.1), *Nitrosopumilus* sp. DDS1 (KR737579.1), *Candidatus* Nitrosopumilus sp. NF5 (CP011070.1), *Candidatus* Nitrosopumilus sp. D3C (CP010868.1)	96%	0	0	0	0	3173
**39237**	LT897242	*Candidatus* Nitrosopelagicus brevis strain CN25 (CP007026.1)	97%	0	0	0	0	600
**39251**	LT897243	*Candidatus* Nitrosopelagicus brevis strain CN25 (CP007026.1)	93%	0	0	0	0	1
**39261**	LT897244	*Candidatus* Nitrosopelagicus brevis strain CN25 (CP007026.1)	96%	0	0	0	0	223
**39262**	LT897245	*Candidatus* Nitrosopelagicus brevis strain CN25 (CP007026.1)	98%	0	0	0	0	130
**39272**	LT897246	*Nitrosopumilus maritimus* strain SCM1 (NR_102913.1), *Candidatus* Nitrosopumilus koreensis strain AR1 (NR_102904.1), *Candidatus* Nitrosopumilus sp. AR2 (CP003843.1), *Candidatus* Nitrosopumilus koreensis AR1 (CP003842.1), *Nitrosopumilus maritimus* SCM1 (CP000866.1)	98%	0	1	0	1	83
**39338**	LT897247	*Nitrosopumilus* sp. DDS1 (KR737579.1), *Candidatus* Nitrosopumilus sp. NF5 (CP011070.1), *Candidatus* Nitrosopumilus sp. D3C (CP010868.1)	95%	0	0	0	0	35
**39362**	LT897248	*Nitrosopumilus* sp. DDS1 (KR737579.1), *Candidatus* Nitrosopumilus sp. NF5 (CP011070.1), *Candidatus* Nitrosopumilus sp. D3C (CP010868.1)	94%	0	0	0	0	6
**39364**	LT897249	*Candidatus* Nitrosopelagicus brevis strain CN25 (CP007026.1)	95%	0	0	0	1	937
**39366**	LT897250	*Candidatus* Nitrosopelagicus brevis strain CN25 (CP007026.1)	95%	0	0	0	0	3
**39395**	LT897251	*Nitrosopumilus* sp. DDS1 (KR737579.1), *Candidatus* Nitrosopumilus sp. NF5 (CP011070.1), *Candidatus* Nitrosopumilus sp. D3C (CP010868.1)	94%	0	0	0	0	93
**39484**	LT897252	*Nitrosopumilus* sp. DDS1 (KR737579.1), *Candidatus* Nitrosopumilus sp. NF5 (CP011070.1), *Candidatus* Nitrosopumilus sp. D3C (CP010868.1)	91%	0	0	0	0	1
**39553**	LT897253	*Nitrosopumilus* sp. DDS1 (KR737579.1), *Candidatus* Nitrosopumilus sp. NF5 (CP011070.1), *Candidatus* Nitrosopumilus sp. D3C (CP010868.1)	95%	0	0	1	0	4

**39557**	LT897254	*Candidatus* Nitrosopelagicus brevis strain CN25 (CP007026.1)	93%	0	0	0	0	4
**39587**	LT897255	*Nitrosopumilus maritimus* strain SCM1 (NR_102913.1), *Candidatus* Nitrosopumilus koreensis strain AR1 (NR_102904.1), *Candidatus* Nitrosopumilus sp. AR2 (CP003843.1), *Candidatus* Nitrosopumilus koreensis AR1 (CP003842.1), *Nitrosopumilus maritimus* SCM1 (CP000866.1)	98%	0	0	0	0	1024
**39630**	LT897256	*Candidatus* Nitrosopelagicus brevis strain CN25 (CP007026.1)	95%	0	0	0	0	2
**39632**	LT897257	*Nitrosopumilus maritimus* strain SCM1 (NR_102913.1), *Candidatus* Nitrosopumilus koreensis strain AR1 (NR_102904.1), *Candidatus* Nitrosopumilus sp. AR2 (CP003843.1), *Candidatus* Nitrosopumilus koreensis AR1 (CP003842.1), *Nitrosopumilus maritimus* SCM1 (CP000866.1)	99%	0	1	0	0	1394
**39644**	LT897258	*Nitrosopumilus maritimus* strain SCM1 (NR_102913.1), *Candidatus* Nitrosopumilus koreensis strain AR1 (NR_102904.1), *Candidatus* Nitrosopumilus sp. AR2 (CP003843.1), *Candidatus* Nitrosopumilus koreensis AR1 (CP003842.1), *Nitrosopumilus maritimus* SCM1 (CP000866.1)	98%	0	0	0	0	23
**39665**	LT897259	*Nitrosopumilus* sp. DDS1 (KR737579.1), *Candidatus* Nitrosopumilus sp. NF5 (CP011070.1), *Candidatus* Nitrosopumilus sp. D3C (CP010868.1)	94%	0	0	0	0	2
**39674**	LT897260	*Nitrosopumilus* sp. DDS1 (KR737579.1), *Candidatus* Nitrosopumilus sp. NF5 (CP011070.1), *Candidatus* Nitrosopumilus sp. D3C (CP010868.1)	96%	0	0	0	0	218
**39675**	LT897261	*Candidatus* Nitrosopelagicus brevis strain CN25 (CP007026.1), *Nitrosopumilus* sp. DDS1 (KR737579.1), *Candidatus* Nitrosopumilus sp. NF5 (CP011070.1), *Candidatus* Nitrosopumilus sp. D3C (CP010868.1)	96%	0	0	0	0	2068
**39699**	LT897262	*Candidatus* Nitrosopelagicus brevis strain CN25 (CP007026.1)	95%	0	1	0	0	583
**39706**	LT897263	*Candidatus* Nitrosopelagicus brevis strain CN25 (CP007026.1)	96%	0	1	0	0	10
**39726**	LT897264	*Nitrosopumilus* sp. DDS1 (KR737579.1), *Candidatus* Nitrosopumilus sp. NF5 (CP011070.1), *Candidatus* Nitrosopumilus sp. D3C (CP010868.1)	96%	0	0	0	0	668

**39748**	LT897265	*Candidatus* Nitrosopelagicus brevis strain CN25 (CP007026.1), *Nitrosopumilus* sp. DDS1 (KR737579.1), *Candidatus* Nitrosopumilus sp. NF5 (CP011070.1), *Candidatus* Nitrosopumilus sp. D3C (CP010868.1)	95%	0	0	0	0	294
**39753**	LT897266	*Candidatus* Nitrosopelagicus brevis strain CN25 (CP007026.1)	88%	0	0	0	0	2
**39756**	LT897267	*Nitrosopumilus maritimus* strain SCM1 (NR_102913.1), *Candidatus* Nitrosopumilus koreensis strain AR1 (NR_102904.1), *Candidatus* Nitrosopumilus sp. AR2 (CP003843.1), *Candidatus* Nitrosopumilus koreensis AR1 (CP003842.1), *Nitrosopumilus maritimus* SCM1 (CP000866.1)	98%	0	0	0	0	2770
**39764**	LT897268	*Candidatus* Nitrosopelagicus brevis strain CN25 (CP007026.1)	94%	0	0	0	0	1
**39777**	LT897269	*Nitrosopumilus* sp. DDS1 (KR737579.1), *Candidatus* Nitrosopumilus sp. NF5 (CP011070.1), *Candidatus* Nitrosopumilus sp. D3C (CP010868.1)	97%	0	0	0	0	1020
**39825**	LT897270	*Nitrosopumilus* sp. DDS1 (KR737579.1), *Candidatus* Nitrosopumilus sp. NF5 (CP011070.1), *Candidatus* Nitrosopumilus sp. D3C (CP010868.1)	96%	0	0	0	0	262
**39871**	LT897271	*Candidatus* Nitrosopelagicus brevis strain CN25 (CP007026.1)	89%	0	0	0	0	1
**39893**	LT897272	*Nitrosopumilus* sp. DDS1 (KR737579.1), *Candidatus* Nitrosopumilus sp. NF5 (CP011070.1), *Candidatus* Nitrosopumilus sp. D3C (CP010868.1)	93%	0	0	0	0	1487
**39903**	LT897273	*Nitrosopumilus* sp. DDS1 (KR737579.1), *Candidatus* Nitrosopumilus sp. NF5 (CP011070.1), *Candidatus* Nitrosopumilus sp. D3C (CP010868.1)	95%	0	0	0	0	40
**39904**	LT897274	*Nitrosopumilus* sp. DDS1 (KR737579.1), *Candidatus* Nitrosopumilus sp. NF5 (CP011070.1), *Candidatus* Nitrosopumilus sp. D3C (CP010868.1)	95%	0	0	0	0	363
**39939**	LT897275	*Nitrosopumilus* sp. DDS1 (KR737579.1), *Candidatus* Nitrosopumilus sp. NF5 (CP011070.1), *Candidatus* Nitrosopumilus sp. D3C (CP010868.1)	92%	0	0	0	0	9
**39995**	LT897276	*Nitrosopumilus* sp. DDS1 (KR737579.1), *Candidatus* Nitrosopumilus sp. NF5 (CP011070.1), *Candidatus* Nitrosopumilus sp. D3C (CP010868.1)	96%	0	1	0	1	9858
**40012**	LT897277	*Nitrosopumilus* sp. DDS1 (KR737579.1), *Candidatus* Nitrosopumilus sp. NF5 (CP011070.1), *Candidatus* Nitrosopumilus sp. D3C (CP010868.1)	95%	0	1	0	0	1184
**40013**	LT897278	*Nitrosopumilus maritimus* strain NAOA6 (KT380502.1), *Candidatus* Nitrosopumilus sp. HCA1 (KF957663.1)	93%	0	0	0	0	5

**40051**	LT897279	*Nitrosopumilus* sp. DDS1 (KR737579.1), *Candidatus* Nitrosopumilus sp. NF5 (CP011070.1), *Candidatus* Nitrosopumilus sp. D3C (CP010868.1)	89%	0	0	0	0	1
**40064**	LT897280	*Nitrosopumilus* sp. DDS1 (KR737579.1), *Candidatus* Nitrosopumilus sp. NF5 (CP011070.1), *Candidatus* Nitrosopumilus sp. D3C (CP010868.1)	95%	1	1	0	0	46
**40077**	LT897281	*Nitrosopumilus* sp. DDS1 (KR737579.1), *Candidatus* Nitrosopumilus sp. NF5 (CP011070.1), *Candidatus* Nitrosopumilus sp. D3C (CP010868.1)	88%	0	0	0	0	1
**40128**	LT897282	*Nitrosopumilus* sp. DDS1 (KR737579.1), *Candidatus* Nitrosopumilus sp. NF5 (CP011070.1), *Candidatus* Nitrosopumilus sp. D3C (CP010868.1)	96%	0	0	0	0	139
**40130**	LT897283	*Nitrosopumilus* sp. DDS1 (KR737579.1), *Candidatus* Nitrosopumilus sp. NF5 (CP011070.1), *Candidatus* Nitrosopumilus sp. D3C (CP010868.1)	96%	0	0	0	0	39
**40131**	LT897284	*Nitrosopumilus* sp. DDS1 (KR737579.1), *Candidatus* Nitrosopumilus sp. NF5 (CP011070.1), *Candidatus* Nitrosopumilus sp. D3C (CP010868.1)	95%	0	0	0	0	14
**40158**	LT897285	*Nitrosopumilus* sp. DDS1 (KR737579.1), *Candidatus* Nitrosopumilus sp. NF5 (CP011070.1), *Candidatus* Nitrosopumilus sp. D3C (CP010868.1)	95%	0	0	0	0	6
**40197**	LT897286	*Nitrosopumilus* sp. DDS1 (KR737579.1), *Candidatus* Nitrosopumilus sp. NF5 (CP011070.1), *Candidatus* Nitrosopumilus sp. D3C (CP010868.1)	93%	0	0	0	0	7
**40231**	LT897287	*Nitrosopumilus* sp. DDS1 (KR737579.1), *Candidatus* Nitrosopumilus sp. NF5 (CP011070.1), *Candidatus* Nitrosopumilus sp. D3C (CP010868.1)	96%	0	0	0	0	33
**40240**	LT897288	*Nitrosopumilus* sp. DDS1 (KR737579.1), *Candidatus* Nitrosopumilus sp. NF5 (CP011070.1), *Candidatus* Nitrosopumilus sp. D3C (CP010868.1)	96%	0	0	0	0	4
**40288**	LT897289	*Nitrosopumilus* sp. DDS1 (KR737579.1), *Candidatus* Nitrosopumilus sp. NF5 (CP011070.1), *Candidatus* Nitrosopumilus sp. D3C (CP010868.1)	92%	0	0	0	0	1
**40315**	LT897290	*Nitrosopumilus* sp. DDS1 (KR737579.1), *Candidatus* Nitrosopumilus sp. NF5 (CP011070.1), *Candidatus* Nitrosopumilus sp. D3C (CP010868.1)	97%	0	0	0	0	201

**40388**	LT897291	*Nitrosopumilus maritimus* strain SCM1 (NR_102913.1), *Candidatus* Nitrosopumilus koreensis strain AR1 (NR_102904.1), *Candidatus* Nitrosopumilus sp. AR2 (CP003843.1), *Nitrosopumilus maritimus* strain NAOA6 (KT380502.1), *Candidatus* Nitrosopumilus sp. HCA1 (KF957663.1)	98%	0	2	0	1	10
**40391**	LT897292	*Nitrosopumilus maritimus* strain NAOA6 (KT380502.1), *Candidatus* Nitrosopumilus sp. HCA1 (KF957663.1)	96%	0	0	0	0	5
**40392**	LT897293	*Nitrosopumilus* sp. DDS1 (KR737579.1), *Candidatus* Nitrosopumilus sp. NF5 (CP011070.1), *Candidatus* Nitrosopumilus sp. D3C (CP010868.1)	95%	0	0	0	0	464
**40397**	LT897294	*Nitrosopumilus* sp. DDS1 (KR737579.1), *Candidatus* Nitrosopumilus sp. NF5 (CP011070.1), *Candidatus* Nitrosopumilus sp. D3C (CP010868.1)	93%	0	0	0	0	19
**40413**	LT897295	*Nitrosopumilus* sp. DDS1 (KR737579.1), *Candidatus* Nitrosopumilus sp. NF5 (CP011070.1), *Candidatus* Nitrosopumilus sp. D3C (CP010868.1)	96%	0	2	0	1	259
**40433**	LT897296	*Nitrosopumilus* sp. DDS1 (KR737579.1), *Candidatus* Nitrosopumilus sp. NF5 (CP011070.1), *Candidatus* Nitrosopumilus sp. D3C (CP010868.1)	95%	0	0	0	2	193
**40461**	LT897297	*Nitrosopumilus* sp. DDS1 (KR737579.1), *Candidatus* Nitrosopumilus sp. NF5 (CP011070.1), *Candidatus* Nitrosopumilus sp. D3C (CP010868.1)	95%	0	0	0	0	224
**40474**	LT897298	*Nitrosopumilus* sp. DDS1 (KR737579.1), *Candidatus* Nitrosopumilus sp. NF5 (CP011070.1), *Candidatus* Nitrosopumilus sp. D3C (CP010868.1)	96%	0	0	0	0	16
**40476**	LT897299	*Nitrosopumilus* sp. DDS1 (KR737579.1), *Candidatus* Nitrosopumilus sp. NF5 (CP011070.1), *Candidatus* Nitrosopumilus sp. D3C (CP010868.1)	95%	0	0	0	0	14
**40487**	LT897300	*Nitrosopumilus* sp. DDS1 (KR737579.1), *Candidatus* Nitrosopumilus sp. NF5 (CP011070.1), *Candidatus* Nitrosopumilus sp. D3C (CP010868.1)	93%	0	0	0	2	41
**40519**	LT897301	*Nitrosopumilus* sp. DDS1 (KR737579.1), *Candidatus* Nitrosopumilus sp. NF5 (CP011070.1), *Candidatus* Nitrosopumilus sp. D3C (CP010868.1)	97%	0	5	2	4	964
**40598**	LT897302	*Nitrosopumilus maritimus* strain NAOA6 (KT380502.1), *Candidatus* Nitrosopumilus sp. HCA1 (KF957663.1)	98%	0	0	0	6	4252
**40605**	LT897303	*Nitrosopumilus* sp. DDS1 (KR737579.1), *Candidatus* Nitrosopumilus sp. NF5 (CP011070.1), *Candidatus* Nitrosopumilus sp. D3C (CP010868.1)	95%	0	0	0	0	106

**40637**	LT897304	*Nitrosopumilus* sp. DDS1 (KR737579.1), *Candidatus* Nitrosopumilus sp. NF5 (CP011070.1), *Candidatus* Nitrosopumilus sp. D3C (CP010868.1)	95%	0	0	0	0	741
**40672**	LT897305	*Candidatus* Nitrosopelagicus brevis strain CN25 (CP007026.1)	94%	0	0	0	2	47
**40681**	LT897306	*Nitrosopumilus* sp. DDS1 (KR737579.1), *Candidatus* Nitrosopumilus sp. NF5 (CP011070.1), *Candidatus* Nitrosopumilus sp. D3C (CP010868.1)	95%	0	0	0	0	17
**40711**	LT897307	*Nitrosopumilus* sp. DDS1 (KR737579.1), *Candidatus* Nitrosopumilus sp. NF5 (CP011070.1), *Candidatus* Nitrosopumilus sp. D3C (CP010868.1)	95%	0	0	0	0	385
**40716**	LT897308	*Nitrosopumilus* sp. DDS1 (KR737579.1), *Candidatus* Nitrosopumilus sp. NF5 (CP011070.1), *Candidatus* Nitrosopumilus sp. D3C (CP010868.1)	98%	0	1	0	0	99
**40717**	LT897309	*Nitrosopumilus* sp. DDS1 (KR737579.1), *Candidatus* Nitrosopumilus sp. NF5 (CP011070.1), *Candidatus* Nitrosopumilus sp. D3C (CP010868.1)	94%	0	0	0	0	63
**40725**	LT897310	*Nitrosopumilus* sp. DDS1 (KR737579.1), *Candidatus* Nitrosopumilus sp. NF5 (CP011070.1), *Candidatus* Nitrosopumilus sp. D3C (CP010868.1)	96%	0	0	0	1	2744
**40734**	LT897311	*Nitrosopumilus* sp. DDS1 (KR737579.1), *Candidatus* Nitrosopumilus sp. NF5 (CP011070.1), *Candidatus* Nitrosopumilus sp. D3C (CP010868.1)	97%	0	1	0	0	1534
**40742**	LT897312	*Nitrosopumilus* sp. DDS1 (KR737579.1), *Candidatus* Nitrosopumilus sp. NF5 (CP011070.1), *Candidatus* Nitrosopumilus sp. D3C (CP010868.1)	94%	0	0	0	0	417
**40767**	LT897313	*Candidatus* Nitrosopelagicus brevis strain CN25 (CP007026.1), *Nitrosopumilus* sp. DDS1 (KR737579.1), *Candidatus* Nitrosopumilus sp. NF5 (CP011070.1), *Candidatus* Nitrosopumilus sp. D3C (CP010868.1)	96%	0	0	0	0	317
**40782**	LT897314	*Candidatus* Nitrosopelagicus brevis strain CN25 (CP007026.1), *Nitrosopumilus* sp. DDS1 (KR737579.1), *Candidatus* Nitrosopumilus sp. NF5 (CP011070.1), *Candidatus* Nitrosopumilus sp. D3C (CP010868.1)	99%	0	2	1	0	77
**40817**	LT897315	*Candidatus* Nitrosopelagicus brevis strain CN25 (CP007026.1), *Nitrosopumilus* sp. DDS1 (KR737579.1), *Candidatus* Nitrosopumilus sp. NF5 (CP011070.1), *Candidatus* Nitrosopumilus sp. D3C (CP010868.1)	96%	0	0	0	0	1774
**40844**	LT897316	*Candidatus* Nitrosopelagicus brevis strain CN25 (CP007026.1)	96%	2	2	0	1	30

**40847**	LT897317	*Candidatus* Nitrosopelagicus brevis strain CN25 (CP007026.1)	100%	0	5	2	1	3406
**40883**	LT897318	*Candidatus* Nitrosopelagicus brevis strain CN25 (CP007026.1), *Nitrosopumilus* sp. DDS1 (KR737579.1), *Candidatus* Nitrosopumilus sp. NF5 (CP011070.1), *Candidatus* Nitrosopumilus sp. D3C (CP010868.1)	96%	0	0	0	0	158
**40895**	LT897319	*Nitrosopumilus maritimus* strain NAOA6 (KT380502.1)	98%	1	0	0	2	18
**40911**	LT897320	*Candidatus* Nitrosopelagicus brevis strain CN25 (CP007026.1), *Nitrosopumilus* sp. DDS1 (KR737579.1), *Candidatus* Nitrosopumilus sp. NF5 (CP011070.1), *Candidatus* Nitrosopumilus sp. D3C (CP010868.1)	96%	0	0	0	0	43
**40943**	LT897321	*Candidatus* Nitrosopelagicus brevis strain CN25 (CP007026.1), *Nitrosopumilus* sp. DDS1 (KR737579.1), *Candidatus* Nitrosopumilus sp. NF5 (CP011070.1), *Candidatus* Nitrosopumilus sp. D3C (CP010868.1)	89%	0	0	0	0	1
**40944**	LT897322	*Candidatus* Nitrosopelagicus brevis strain CN25 (CP007026.1), *Nitrosopumilus* sp. DDS1 (KR737579.1), *Candidatus* Nitrosopumilus sp. NF5 (CP011070.1), *Candidatus* Nitrosopumilus sp. D3C (CP010868.1)	96%	0	0	0	1	2104
**40957**	LT897323	*Candidatus* Nitrosopelagicus brevis strain CN25 (CP007026.1)	95%	0	0	0	0	24
**40987**	LT897324	*Candidatus* Nitrosopelagicus brevis strain CN25 (CP007026.1)	98%	1	240	20	185	3557
**40990**	LT897325	*Candidatus* Nitrosopelagicus brevis strain CN25 (CP007026.1)	96%	0	0	0	0	1
**41009**	LT897326	*Candidatus* Nitrosopelagicus brevis strain CN25 (CP007026.1)	96%	0	3	2	10	640
**41019**	LT897327	*Candidatus* Nitrosopelagicus brevis strain CN25 (CP007026.1)	97%	0	3	0	12	106
**41032**	LT897328	*Candidatus* Nitrosopelagicus brevis strain CN25 (CP007026.1), *Nitrosopumilus* sp. DDS1 (KR737579.1), *Candidatus* Nitrosopumilus sp. NF5 (CP011070.1), *Candidatus* Nitrosopumilus sp. D3C (CP010868.1)	96%	0	0	0	0	739
**41045**	LT897329	*Candidatus* Nitrosopelagicus brevis strain CN25 (CP007026.1), *Nitrosopumilus* sp. DDS1 (KR737579.1), *Candidatus* Nitrosopumilus sp. NF5 (CP011070.1), *Candidatus* Nitrosopumilus sp. D3C (CP010868.1)	95%	0	0	1	0	528
**41075**	LT897330	*Candidatus* Nitrosopelagicus brevis strain CN25 (CP007026.1)	94%	0	3	0	3	1245
**41092**	LT897331	*Candidatus* Nitrosopelagicus brevis strain CN25 (CP007026.1)	88%	0	0	0	0	1

**41093**	LT897332	*Candidatus* Nitrosopelagicus brevis strain CN25 (CP007026.1), *Nitrosopumilus* sp. DDS1 (KR737579.1), *Candidatus* Nitrosopumilus sp. NF5 (CP011070.1), *Candidatus* Nitrosopumilus sp. D3C (CP010868.1)	95%	0	0	1	3	4573
**41120**	LT897333	*Nitrosopumilus maritimus* strain NAOA6 (KT380502.1), *Candidatus* Nitrosopumilus sp. HCA1 (KF957663.1)	99%	0	0	0	0	107
**41123**	LT897334	*Candidatus* Nitrosopelagicus brevis strain CN25 (CP007026.1)	97%	0	0	0	0	39
**41130**	LT897335	*Candidatus* Nitrosopelagicus brevis strain CN25 (CP007026.1), *Nitrosopumilus* sp. DDS1 (KR737579.1), *Candidatus* Nitrosopumilus sp. NF5 (CP011070.1), *Candidatus* Nitrosopumilus sp. D3C (CP010868.1)	96%	0	0	0	0	467
**41149**	LT897336	*Candidatus* Nitrosopelagicus brevis strain CN25 (CP007026.1)	95%	0	2	0	0	4
**41161**	LT897337	*Candidatus* Nitrosopelagicus brevis strain CN25 (CP007026.1), *Nitrosopumilus* sp. DDS1 (KR737579.1), *Candidatus* Nitrosopumilus sp. NF5 (CP011070.1), *Candidatus* Nitrosopumilus sp. D3C (CP010868.1)	96%	0	0	0	0	106
**41172**	LT897338	*Candidatus* Nitrosopelagicus brevis strain CN25 (CP007026.1), *Nitrosopumilus* sp. DDS1 (KR737579.1), *Candidatus* Nitrosopumilus sp. NF5 (CP011070.1), *Candidatus* Nitrosopumilus sp. D3C (CP010868.1)	86%	0	0	0	0	1
**41200**	LT897339	*Candidatus* Nitrosopelagicus brevis strain CN25 (CP007026.1)	95%	0	0	0	0	1
**41216**	LT897340	*Candidatus* Nitrosopelagicus brevis strain CN25 (CP007026.1)	96%	0	0	0	0	29
**41218**	LT897341	*Candidatus* Nitrosopelagicus brevis strain CN25 (CP007026.1), *Nitrosopumilus* sp. DDS1 (KR737579.1), *Candidatus* Nitrosopumilus sp. NF5 (CP011070.1), *Candidatus* Nitrosopumilus sp. D3C (CP010868.1)	93%	0	0	0	0	1
**41239**	LT897342	*Candidatus* Nitrosopelagicus brevis strain CN25 (CP007026.1), *Nitrosopumilus* sp. DDS1 (KR737579.1), *Candidatus* Nitrosopumilus sp. NF5 (CP011070.1), *Candidatus* Nitrosopumilus sp. D3C (CP010868.1)	97%	0	0	0	3	3701
**41264**	LT897343	*Candidatus* Nitrosopelagicus brevis strain CN25 (CP007026.1)	89%	0	0	0	0	1
**41281**	LT897344	*Candidatus* Nitrosopelagicus brevis strain CN25 (CP007026.1)	96%	0	0	0	0	9

**41306**	LT897345	*Candidatus* Nitrosopelagicus brevis strain CN25 (CP007026.1), *Nitrosopumilus* sp. DDS1 (KR737579.1), *Candidatus* Nitrosopumilus sp. NF5 (CP011070.1), *Candidatus* Nitrosopumilus sp. D3C (CP010868.1)	93%	0	0	0	0	3
**41324**	LT897346	*Candidatus* Nitrosopelagicus brevis strain CN25 (CP007026.1)	95%	0	0	0	0	3
**41326**	LT897347	*Candidatus* Nitrosopelagicus brevis strain CN25 (CP007026.1), *Nitrosopumilus* sp. DDS1 (KR737579.1), *Candidatus* Nitrosopumilus sp. NF5 (CP011070.1), *Candidatus* Nitrosopumilus sp. D3C (CP010868.1)	95%	0	0	0	0	3
**41328**	LT897348	*Candidatus* Nitrosopelagicus brevis strain CN25 (CP007026.1)	95%	0	4	1	4	2548
**41360**	LT897349	*Candidatus* Nitrosopelagicus brevis strain CN25 (CP007026.1)	95%	0	13	0	12	919
**41363**	LT897350	*Candidatus* Nitrosopelagicus brevis strain CN25 (CP007026.1), *Nitrosopumilus* sp. DDS1 (KR737579.1), *Candidatus* Nitrosopumilus sp. NF5 (CP011070.1), *Candidatus* Nitrosopumilus sp. D3C (CP010868.1)	90%	0	0	0	0	3
**41364**	LT897351	*Candidatus* Nitrosopelagicus brevis strain CN25 (CP007026.1)	95%	0	0	0	0	245
**41407**	LT897352	*Candidatus* Nitrosopelagicus brevis strain CN25 (CP007026.1), *Nitrosopumilus* sp. DDS1 (KR737579.1), *Candidatus* Nitrosopumilus sp. NF5 (CP011070.1), *Candidatus* Nitrosopumilus sp. D3C (CP010868.1)	87%	0	0	0	0	1
**41458**	LT897353	*Candidatus* Nitrosopelagicus brevis strain CN25 (CP007026.1), *Nitrosopumilus* sp. DDS1 (KR737579.1), *Candidatus* Nitrosopumilus sp. NF5 (CP011070.1), *Candidatus* Nitrosopumilus sp. D3C (CP010868.1)	97%	1	1	0	1	1311
**41471**	LT897354	*Nitrosopumilus maritimus* strain NAOA6 (KT380502.1), *Candidatus* Nitrosopumilus sp. HCA1 (KF957663.1)	99%	0	1	0	1	41
**41520**	LT897355	*Candidatus* Nitrosopelagicus brevis strain CN25 (CP007026.1)	95%	0	0	0	0	544
**41559**	LT897356	*Candidatus* Nitrosopelagicus brevis strain CN25 (CP007026.1), *Nitrosopumilus* sp. DDS1 (KR737579.1), *Candidatus* Nitrosopumilus sp. NF5 (CP011070.1), *Candidatus* Nitrosopumilus sp. D3C (CP010868.1)	96%	0	1	0	0	2542
**41560**	LT897357	*Candidatus* Nitrosopelagicus brevis strain CN25 (CP007026.1), *Nitrosopumilus* sp. DDS1 (KR737579.1), *Candidatus* Nitrosopumilus sp. NF5 (CP011070.1), *Candidatus* Nitrosopumilus sp. D3C (CP010868.1)	90%	0	0	0	0	1

**41575**	LT897358	*Nitrosopumilus maritimus* strain SCM1 (NR_102913.1), *Candidatus* Nitrosopumilus koreensis strain AR1 (NR_102904.1), *Candidatus* Nitrosopumilus sp. AR2 (CP003843.1), *Nitrosopumilus maritimus* strain NAOA6 (KT380502.1), *Candidatus* Nitrosopumilus sp. HCA1 (KF957663.1)	98%	1	1	0	1	4913
**41580**	LT897359	*Candidatus* Nitrosopelagicus brevis strain CN25 (CP007026.1), *Nitrosopumilus* sp. DDS1 (KR737579.1), *Candidatus* Nitrosopumilus sp. NF5 (CP011070.1), *Candidatus* Nitrosopumilus sp. D3C (CP010868.1)	95%	0	0	0	0	1
**41590**	LT897360	*Candidatus* Nitrosopelagicus brevis strain CN25 (CP007026.1)	98%	0	9	1	7	1765
**41600**	LT897361	*Candidatus* Nitrosopelagicus brevis strain CN25 (CP007026.1)	98%	0	0	0	0	2
**41653**	LT897362	*Candidatus* Nitrosopelagicus brevis strain CN25 (CP007026.1), *Nitrosopumilus* sp. DDS1 (KR737579.1), *Candidatus* Nitrosopumilus sp. NF5 (CP011070.1), *Candidatus* Nitrosopumilus sp. D3C (CP010868.1)	95%	0	0	0	0	1
**41654**	LT897363	*Candidatus* Nitrosopelagicus brevis strain CN25 (CP007026.1)	91%	0	0	0	0	1
**41680**	LT897364	*Candidatus* Nitrosopelagicus brevis strain CN25 (CP007026.1), *Nitrosopumilus* sp. DDS1 (KR737579.1), *Candidatus* Nitrosopumilus sp. NF5 (CP011070.1), *Candidatus* Nitrosopumilus sp. D3C (CP010868.1)	90%	0	0	0	0	1
**41684**	LT897365	*Candidatus* Nitrosopelagicus brevis strain CN25 (CP007026.1)	95%	0	0	0	1	20
**41686**	LT897366	*Candidatus* Nitrosopelagicus brevis strain CN25 (CP007026.1)	91%	0	0	0	0	2
**41699**	LT897367	*Candidatus* Nitrosopelagicus brevis strain CN25 (CP007026.1), *Nitrosopumilus* sp. DDS1 (KR737579.1), *Candidatus* Nitrosopumilus sp. NF5 (CP011070.1), *Candidatus* Nitrosopumilus sp. D3C (CP010868.1)	96%	0	0	0	0	3
**41701**	LT897368	*Candidatus* Nitrosopelagicus brevis strain CN25 (CP007026.1), *Nitrosopumilus* sp. DDS1 (KR737579.1), *Candidatus* Nitrosopumilus sp. NF5 (CP011070.1), *Candidatus* Nitrosopumilus sp. D3C (CP010868.1)	98%	0	3	0	1	55

**41702**	LT897369	*Nitrosopumilus maritimus* strain SCM1 (NR_102913.1), *Candidatus* Nitrosopumilus koreensis strain AR1 (NR_102904.1), *Candidatus* Nitrosopumilus sp. AR2 (CP003843.1), *Nitrosopumilus maritimus* strain NAOA6 (KT380502.1), *Candidatus* Nitrosopumilus sp. HCA1 (KF957663.1)	99%	0	0	1	0	7
**41742**	LT897370	*Candidatus* Nitrosopelagicus brevis strain CN25 (CP007026.1), *Nitrosopumilus* sp. DDS1 (KR737579.1), *Candidatus* Nitrosopumilus sp. NF5 (CP011070.1), *Candidatus* Nitrosopumilus sp. D3C (CP010868.1)	96%	0	0	0	0	13
**41778**	LT897371	*Candidatus* Nitrosopelagicus brevis strain CN25 (CP007026.1), *Nitrosopumilus* sp. DDS1 (KR737579.1), *Candidatus* Nitrosopumilus sp. NF5 (CP011070.1), *Candidatus* Nitrosopumilus sp. D3C (CP010868.1)	97%	0	5	3	6	455
**41823**	LT897372	*Nitrosopumilus maritimus* strain NAOA6 (KT380502.1), *Candidatus* Nitrosopumilus sp. HCA1 (KF957663.1)	90%	0	0	0	1	1
**41845**	LT897373	*Candidatus* Nitrosopelagicus brevis strain CN25 (CP007026.1), *Nitrosopumilus* sp. DDS1 (KR737579.1), *Candidatus* Nitrosopumilus sp. NF5 (CP011070.1), *Candidatus* Nitrosopumilus sp. D3C (CP010868.1)	93%	0	0	0	0	119
**41849**	LT897374	*Candidatus* Nitrosopelagicus brevis strain CN25 (CP007026.1)	95%	0	0	0	0	40
**41854**	LT897375	*Nitrosopumilus maritimus* strain NAOA6 (KT380502.1), *Nitrosopumilus maritimus* strain SCM1 (NR_102913.1), *Candidatus* Nitrosopumilus koreensis strain AR1 (NR_102904.1), *Candidatus* Nitrosopumilus sp. AR2 (CP003843.1), *Nitrosopumilus maritimus* strain NAOA6 (KT380502.1), *Candidatus* Nitrosopumilus sp. HCA1 (KF957663.1)	97%	0	0	0	0	1
**41860**	LT897376	*Nitrosopumilus maritimus* strain NAOA6 (KT380502.1), *Nitrosopumilus maritimus* strain SCM1 (NR_102913.1), *Candidatus* Nitrosopumilus koreensis strain AR1 (NR_102904.1), *Candidatus* Nitrosopumilus sp. AR2 (CP003843.1), *Nitrosopumilus maritimus* strain NAOA6 (KT380502.1), *Candidatus* Nitrosopumilus sp. HCA1 (KF957663.1)	99%	0	20	0	11	115
**41868**	LT897377	*Candidatus* Nitrosopelagicus brevis strain CN25 (CP007026.1)	90%	0	0	0	0	2
**41879**	LT897378	*Nitrosopumilus maritimus* strain NAOA6 (KT380502.1), *Candidatus* Nitrosopumilus sp. HCA1 (KF957663.1)	99%	16	590	27	126	2624

**41880**	LT897379	*Candidatus* Nitrosopelagicus brevis strain CN25 (CP007026.1), *Nitrosopumilus* sp. DDS1 (KR737579.1), *Candidatus* Nitrosopumilus sp. NF5 (CP011070.1), *Candidatus* Nitrosopumilus sp. D3C (CP010868.1)	91%	0	0	0	0	1
**41881**	LT897380	*Candidatus* Nitrosopelagicus brevis strain CN25 (CP007026.1), *Nitrosopumilus* sp. DDS1 (KR737579.1), *Candidatus* Nitrosopumilus sp. NF5 (CP011070.1), *Candidatus* Nitrosopumilus sp. D3C (CP010868.1)	96%	0	0	0	0	20
**41885**	LT897381	*Nitrosopumilus maritimus* strain NAOA6 (KT380502.1), *Candidatus* Nitrosopumilus sp. HCA1 (KF957663.1)	99%	0	9	0	4	93
**41920**	LT897382	*Nitrosopumilus maritimus* strain NAOA6 (KT380502.1), *Nitrosopumilus maritimus* strain SCM1 (NR_102913.1), *Candidatus* Nitrosopumilus koreensis strain AR1 (NR_102904.1), *Candidatus* Nitrosopumilus sp. AR2 (CP003843.1), *Candidatus* Nitrosopumilus sp. HCA1 (KF957663.1)	89%	0	0	0	0	1
**41945**	LT897383	*Candidatus* Nitrosopelagicus brevis strain CN25 (CP007026.1)	96%	0	0	0	0	22
**41956**	LT897384	*Candidatus* Nitrosopelagicus brevis strain CN25 (CP007026.1)	94%	0	0	0	0	1
**41965**	LT897385	*Candidatus* Nitrosopelagicus brevis strain CN25 (CP007026.1), *Nitrosopumilus* sp. DDS1 (KR737579.1), *Candidatus* Nitrosopumilus sp. NF5 (CP011070.1), *Candidatus* Nitrosopumilus sp. D3C (CP010868.1)	96%	0	0	0	0	2092
**41983**	LT897386	*Candidatus* Nitrosopelagicus brevis strain CN25 (CP007026.1)	95%	0	0	0	0	366
**41984**	LT897387	*Candidatus* Nitrosopelagicus brevis strain CN25 (CP007026.1)	96%	0	0	1	0	3
**41989**	LT897388	*Candidatus* Nitrosopelagicus brevis strain CN25 (CP007026.1)	96%	0	0	0	0	221
**42018**	LT897389	*Nitrosopumilus maritimus* strain NAOA6 (KT380502.1), *Nitrosopumilus maritimus* strain SCM1 (NR_102913.1), *Candidatus* Nitrosopumilus koreensis strain AR1 (NR_102904.1), *Candidatus* Nitrosopumilus sp. AR2 (CP003843.1), *Candidatus* Nitrosopumilus sp. HCA1 (KF957663.1)	99%	1	64	5	18	69
**42026**	LT897390	*Candidatus* Nitrosopelagicus brevis strain CN25 (CP007026.1), *Nitrosopumilus* sp. DDS1 (KR737579.1), *Candidatus* Nitrosopumilus sp. NF5 (CP011070.1), *Candidatus* Nitrosopumilus sp. D3C (CP010868.1)	95%	0	0	0	1	91

**42035**	LT897391	*Candidatus* Nitrosopelagicus brevis strain CN25 (CP007026.1), *Nitrosopumilus* sp. DDS1 (KR737579.1), *Candidatus* Nitrosopumilus sp. NF5 (CP011070.1), *Candidatus* Nitrosopumilus sp. D3C (CP010868.1)	95%	0	0	0	0	1414
**42068**	LT897392	*Nitrosopumilus maritimus* strain NAOA6 (KT380502.1), *Candidatus* Nitrosopumilus sp. HCA1 (KF957663.1)	100%	38	1425	61	306	5528
**42086**	LT897393	*Candidatus* Nitrosopelagicus brevis strain CN25 (CP007026.1), *Nitrosopumilus* sp. DDS1 (KR737579.1), *Candidatus* Nitrosopumilus sp. NF5 (CP011070.1), *Candidatus* Nitrosopumilus sp. D3C (CP010868.1)	93%	0	0	0	0	1
**42091**	LT897394	*Candidatus* Nitrosopelagicus brevis strain CN25 (CP007026.1), *Nitrosopumilus* sp. DDS1 (KR737579.1), *Candidatus* Nitrosopumilus sp. NF5 (CP011070.1), *Candidatus* Nitrosopumilus sp. D3C (CP010868.1)	94%	0	0	0	0	1
**42112**	LT897395	*Candidatus* Nitrosopumilus sp. NF5 (CP011070.1), *Candidatus* Nitrosopumilus sp. D3C (CP010868.1)	94%	0	0	0	0	51
**42126**	LT897396	*Nitrosopumilus maritimus* strain NAOA6 (KT380502.1), *Candidatus* Nitrosopumilus sp. HCA1 (KF957663.1)	89%	0	0	0	0	1
**42135**	LT897397	*Candidatus* Nitrosopelagicus brevis strain CN25 (CP007026.1)	97%	0	0	1	2	416
**42163**	LT897398	*Candidatus* Nitrosopelagicus brevis strain CN25 (CP007026.1)	95%	0	0	0	0	1
**42165**	LT897399	*Candidatus* Nitrosopelagicus brevis strain CN25 (CP007026.1)	96%	0	14	3	12	686
**42186**	LT897400	*Candidatus* Nitrosopelagicus brevis strain CN25 (CP007026.1)	96%	0	0	0	0	6
**42202**	LT897401	*Candidatus* Nitrosopelagicus brevis strain CN25 (CP007026.1), *Nitrosopumilus* sp. DDS1 (KR737579.1), *Candidatus* Nitrosopumilus sp. NF5 (CP011070.1), *Candidatus* Nitrosopumilus sp. D3C (CP010868.1)	91%	0	0	0	0	2
**42223**	LT897402	*Nitrosopumilus maritimus* strain NAOA6 (KT380502.1), *Nitrosopumilus maritimus* strain SCM1 (NR_102913.1), *Candidatus* Nitrosopumilus koreensis strain AR1 (NR_102904.1), *Candidatus* Nitrosopumilus sp. AR2 (CP003843.1), *Candidatus* Nitrosopumilus sp. HCA1 (KF957663.1)	98%	0	9	1	4	2591
**42274**	LT897403	*Nitrosopumilus maritimus* strain NAOA6 (KT380502.1), *Candidatus* Nitrosopumilus sp. HCA1 (KF957663.1)	91%	0	0	0	0	1

**42283**	LT897404	*Candidatus* Nitrosopelagicus brevis strain CN25 (CP007026.1), *Nitrosopumilus* sp. DDS1 (KR737579.1), *Candidatus* Nitrosopumilus sp. NF5 (CP011070.1), *Candidatus* Nitrosopumilus sp. D3C (CP010868.1)	92%	0	0	0	0	2
**42287**	LT897405	*Candidatus* Nitrosopelagicus brevis strain CN25 (CP007026.1)	96%	0	0	0	0	1
**42317**	LT897406	*Nitrosopumilus maritimus* strain NAOA6 (KT380502.1), *Candidatus* Nitrosopumilus sp. HCA1 (KF957663.1)	94%	0	0	0	0	2
**42331**	LT897407	*Candidatus* Nitrosopelagicus brevis strain CN25 (CP007026.1)	89%	0	0	0	0	1
**42350**	LT897408	*Candidatus* Nitrosopelagicus brevis strain CN25 (CP007026.1)	96%	0	3	1	2	127
**42371**	LT897409	*Candidatus* Nitrosopelagicus brevis strain CN25 (CP007026.1), *Nitrosopumilus* sp. DDS1 (KR737579.1), *Candidatus* Nitrosopumilus sp. NF5 (CP011070.1), *Candidatus* Nitrosopumilus sp. D3C (CP010868.1)	95%	0	0	0	0	40
**42376**	LT897410	*Candidatus* Nitrosopelagicus brevis strain CN25 (CP007026.1), *Nitrosopumilus* sp. DDS1 (KR737579.1), *Candidatus* Nitrosopumilus sp. NF5 (CP011070.1), *Candidatus* Nitrosopumilus sp. D3C (CP010868.1)	95%	0	0	0	0	29
**42394**	LT897411	*Nitrosopumilus maritimus* strain NAOA6 (KT380502.1), *Candidatus* Nitrosopumilus sp. HCA1 (KF957663.1)	92%	0	0	0	0	1
**42399**	LT897412	*Candidatus* Nitrosopelagicus brevis strain CN25 (CP007026.1), *Nitrosopumilus* sp. DDS1 (KR737579.1), *Candidatus* Nitrosopumilus sp. NF5 (CP011070.1), *Candidatus* Nitrosopumilus sp. D3C (CP010868.1)	88%	0	0	0	0	1
**42400**	LT897413	*Candidatus* Nitrosopelagicus brevis strain CN25 (CP007026.1)	94%	0	3	0	3	19
**42438**	LT897414	*Candidatus* Nitrosopelagicus brevis strain CN25 (CP007026.1), *Nitrosopumilus* sp. DDS1 (KR737579.1), *Candidatus* Nitrosopumilus sp. NF5 (CP011070.1), *Candidatus* Nitrosopumilus sp. D3C (CP010868.1)	96%	0	1	0	1	26
**42440**	LT897415	*Candidatus* Nitrosopelagicus brevis strain CN25 (CP007026.1), *Nitrosopumilus* sp. DDS1 (KR737579.1), *Candidatus* Nitrosopumilus sp. NF5 (CP011070.1), *Candidatus* Nitrosopumilus sp. D3C (CP010868.1)	96%	0	4	2	3	1081
**42445**	LT897416	*Candidatus* Nitrosopelagicus brevis strain CN25 (CP007026.1)	98%	4	487	32	377	834

**42454**	LT897417	*Candidatus* Nitrosopelagicus brevis strain CN25 (CP007026.1), *Nitrosopumilus* sp. DDS1 (KR737579.1), *Candidatus* Nitrosopumilus sp. NF5 (CP011070.1), *Candidatus* Nitrosopumilus sp. D3C (CP010868.1)	91%	0	0	0	0	2
**42456**	LT897418	*Nitrosopumilus maritimus* strain NAOA6 (KT380502.1), *Nitrosopumilus maritimus* strain SCM1 (NR_102913.1), *Candidatus* Nitrosopumilus koreensis strain AR1 (NR_102904.1), *Candidatus* Nitrosopumilus sp. AR2 (CP003843.1), *Candidatus* Nitrosopumilus sp. HCA1 (KF957663.1)	100%	4	403	6	69	86
**42462**	LT897419	*Candidatus* Nitrosopelagicus brevis strain CN25 (CP007026.1)	96%	0	0	0	0	25
**42464**	LT897420	*Nitrosopumilus* sp. DDS1 (KR737579.1)	89%	0	0	0	0	1
**42494**	LT897421	*Candidatus* Nitrosopelagicus brevis strain CN25 (CP007026.1), *Nitrosopumilus* sp. DDS1 (KR737579.1), *Candidatus* Nitrosopumilus sp. NF5 (CP011070.1), *Candidatus* Nitrosopumilus sp. D3C (CP010868.1)	96%	0	3	0	8	719
**42510**	LT897422	*Nitrosopumilus* sp. DDS1 (KR737579.1)	95%	0	0	0	0	1
**42516**	LT897423	*Nitrosopumilus* sp. DDS1 (KR737579.1)	100%	7	244	11	99	92
**42533**	LT897424	*Candidatus* Nitrosopelagicus brevis strain CN25 (CP007026.1), *Nitrosopumilus* sp. DDS1 (KR737579.1), *Candidatus* Nitrosopumilus sp. NF5 (CP011070.1), *Candidatus* Nitrosopumilus sp. D3C (CP010868.1)	90%	0	0	0	0	1
**42536**	LT897425	*Candidatus* Nitrosopelagicus brevis strain CN25 (CP007026.1)	95%	0	24	1	34	306
**42571**	LT897426	*Candidatus* Nitrosopelagicus brevis strain CN25 (CP007026.1)	95%	0	8	3	18	400
**42576**	LT897427	*Candidatus* Nitrosopelagicus brevis strain CN25 (CP007026.1)	97%	0	9	1	13	196
**42596**	LT897428	*Candidatus* Nitrosopelagicus brevis strain CN25 (CP007026.1), *Nitrosopumilus* sp. DDS1 (KR737579.1), *Candidatus* Nitrosopumilus sp. NF5 (CP011070.1), *Candidatus* Nitrosopumilus sp. D3C (CP010868.1)	95%	0	0	0	0	1
**42607**	LT897429	*Candidatus* Nitrosopelagicus brevis strain CN25 (CP007026.1)	95%	0	26	4	48	399
**42623**	LT897430	*Nitrosopumilus maritimus* strain NAOA6 (KT380502.1), *Nitrosopumilus maritimus* strain SCM1 (NR_102913.1), *Candidatus* Nitrosopumilus koreensis strain AR1 (NR_102904.1), *Candidatus* Nitrosopumilus sp. AR2 (CP003843.1), *Candidatus* Nitrosopumilus sp. HCA1 (KF957663.1)	98%	2	272	19	169	2213
**42638**	LT897431	*Candidatus* Nitrosopelagicus brevis strain CN25 (CP007026.1)	94%	0	0	0	1	14

**42693**	LT897432	*Candidatus* Nitrosopelagicus brevis strain CN25 (CP007026.1), *Nitrosopumilus* sp. DDS1 (KR737579.1), *Candidatus* Nitrosopumilus sp. NF5 (CP011070.1), *Candidatus* Nitrosopumilus sp. D3C (CP010868.1)	93%	0	0	0	0	1
**42725**	LT897433	*Candidatus* Nitrosopelagicus brevis strain CN25 (CP007026.1), *Nitrosopumilus* sp. DDS1 (KR737579.1)	95%	0	0	0	0	17
**42734**	LT897434	*Candidatus* Nitrosopelagicus brevis strain CN25 (CP007026.1), *Nitrosopumilus* sp. DDS1 (KR737579.1), *Candidatus* Nitrosopumilus sp. NF5 (CP011070.1), *Candidatus* Nitrosopumilus sp. D3C (CP010868.1)	96%	0	1	0	1	148
**42754**	LT897435	*Candidatus* Nitrosopelagicus brevis strain CN25 (CP007026.1), *Nitrosopumilus* sp. DDS1 (KR737579.1), *Candidatus* Nitrosopumilus sp. NF5 (CP011070.1), *Candidatus* Nitrosopumilus sp. D3C (CP010868.1)	96%	0	4	0	2	1243
**42787**	LT897436	*Nitrosopumilus maritimus* strain NAOA6 (KT380502.1), *Nitrosopumilus maritimus* strain SCM1 (NR_102913.1), *Candidatus* Nitrosopumilus koreensis strain AR1 (NR_102904.1), *Candidatus* Nitrosopumilus sp. AR2 (CP003843.1), *Candidatus* Nitrosopumilus sp. HCA1 (KF957663.1)	97%	0	2	0	0	1
**42793**	LT897437	*Nitrosopumilus maritimus* strain NAOA6 (KT380502.1), *Candidatus* Nitrosopumilus sp. HCA1 (KF957663.1)	91%	0	0	0	0	1
**42825**	LT897438	*Candidatus* Nitrosopelagicus brevis strain CN25 (CP007026.1), *Nitrosopumilus* sp. DDS1 (KR737579.1), *Candidatus* Nitrosopumilus sp. NF5 (CP011070.1), *Candidatus* Nitrosopumilus sp. D3C (CP010868.1)	96%	0	4	1	2	960
**42849**	LT897439	*Candidatus* Nitrosopelagicus brevis strain CN25 (CP007026.1), *Nitrosopumilus* sp. DDS1 (KR737579.1), *Candidatus* Nitrosopumilus sp. NF5 (CP011070.1), *Candidatus* Nitrosopumilus sp. D3C (CP010868.1)	94%	0	0	0	0	3
**42861**	LT897440	*Candidatus* Nitrosopelagicus brevis strain CN25 (CP007026.1), *Nitrosopumilus* sp. DDS1 (KR737579.1), *Candidatus* Nitrosopumilus sp. NF5 (CP011070.1), *Candidatus* Nitrosopumilus sp. D3C (CP010868.1)	97%	0	0	0	0	1

**42869**	LT897441	*Candidatus* Nitrosopelagicus brevis strain CN25 (CP007026.1), *Nitrosopumilus* sp. DDS1 (KR737579.1), *Candidatus* Nitrosopumilus sp. NF5 (CP011070.1), *Candidatus* Nitrosopumilus sp. D3C (CP010868.1)	92%	0	0	0	0	1
**42871**	LT897442	*Nitrosopumilus maritimus* strain NAOA6 (KT380502.1), *Nitrosopumilus maritimus* strain SCM1 (NR_102913.1), *Candidatus* Nitrosopumilus koreensis strain AR1 (NR_102904.1), *Candidatus* Nitrosopumilus sp. AR2 (CP003843.1), *Candidatus* Nitrosopumilus sp. HCA1 (KF957663.1)	94%	0	0	0	0	1
**42878**	LT897443	*Candidatus* Nitrosopelagicus brevis strain CN25 (CP007026.1), *Nitrosopumilus* sp. DDS1 (KR737579.1), *Candidatus* Nitrosopumilus sp. NF5 (CP011070.1), *Candidatus* Nitrosopumilus sp. D3C (CP010868.1)	95%	0	0	0	0	1
**42896**	LT897444	*Candidatus* Nitrosopelagicus brevis strain CN25 (CP007026.1)	95%	0	0	0	0	1
**42901**	LT897445	*Candidatus* Nitrosopelagicus brevis strain CN25 (CP007026.1)	95%	0	0	0	0	1
**42902**	LT897446	*Candidatus* Nitrosopelagicus brevis strain CN25 (CP007026.1)	92%	0	0	0	0	1
**42915**	LT897447	*Candidatus* Nitrosopelagicus brevis strain CN25 (CP007026.1), *Nitrosopumilus* sp. DDS1 (KR737579.1), *Candidatus* Nitrosopumilus sp. NF5 (CP011070.1), *Candidatus* Nitrosopumilus sp. D3C (CP010868.1)	96%	0	0	0	0	1
**42956**	LT897448	*Candidatus* Nitrosopelagicus brevis strain CN25 (CP007026.1), *Nitrosopumilus* sp. DDS1 (KR737579.1), *Candidatus* Nitrosopumilus sp. NF5 (CP011070.1), *Candidatus* Nitrosopumilus sp. D3C (CP010868.1)	96%	0	0	0	0	1
**42976**	LT897449	*Candidatus* Nitrosopelagicus brevis strain CN25 (CP007026.1), *Nitrosopumilus* sp. DDS1 (KR737579.1), *Candidatus* Nitrosopumilus sp. NF5 (CP011070.1), *Candidatus* Nitrosopumilus sp. D3C (CP010868.1)	95%	0	0	0	0	1
**42992**	LT897450	*Candidatus* Nitrosopelagicus brevis strain CN25 (CP007026.1), *Nitrosopumilus* sp. DDS1 (KR737579.1), *Candidatus* Nitrosopumilus sp. NF5 (CP011070.1), *Candidatus* Nitrosopumilus sp. D3C (CP010868.1)	94%	0	0	0	0	1

**42996**	LT897451	*Nitrosopumilus maritimus* strain NAOA6 (KT380502.1), *Nitrosopumilus maritimus* strain SCM1 (NR_102913.1), *Candidatus* Nitrosopumilus koreensis strain AR1 (NR_102904.1), *Candidatus* Nitrosopumilus sp. AR2 (CP003843.1), *Candidatus* Nitrosopumilus sp. HCA1 (KF957663.1)	96%	0	0	0	0	1
**43024**	LT897452	*Candidatus* Nitrosopelagicus brevis strain CN25 (CP007026.1)	94%	0	0	0	0	1
**43031**	LT897453	*Nitrosopumilus maritimus* strain NAOA6 (KT380502.1), *Nitrosopumilus maritimus* strain SCM1 (NR_102913.1), *Candidatus* Nitrosopumilus koreensis strain AR1 (NR_102904.1), *Candidatus* Nitrosopumilus sp. AR2 (CP003843.1), *Candidatus* Nitrosopumilus sp. HCA1 (KF957663.1)	97%	0	0	0	0	1
**43043**	LT897454	*Nitrosopumilus maritimus* strain NAOA6 (KT380502.1), *Nitrosopumilus maritimus* strain SCM1 (NR_102913.1), *Candidatus* Nitrosopumilus koreensis strain AR1 (NR_102904.1), *Candidatus* Nitrosopumilus sp. AR2 (CP003843.1), *Candidatus* Nitrosopumilus sp. HCA1 (KF957663.1)	96%	0	0	0	0	2
**43049**	LT897455	*Candidatus* Nitrosopelagicus brevis strain CN25 (CP007026.1), *Nitrosopumilus* sp. DDS1 (KR737579.1), *Candidatus* Nitrosopumilus sp. NF5 (CP011070.1), *Candidatus* Nitrosopumilus sp. D3C (CP010868.1)	93%	0	0	0	0	1
**43050**	LT897456	*Nitrosopumilus maritimus* strain NAOA6 (KT380502.1), *Nitrosopumilus maritimus* strain SCM1 (NR_102913.1), *Candidatus* Nitrosopumilus koreensis strain AR1 (NR_102904.1), *Candidatus* Nitrosopumilus sp. AR2 (CP003843.1), *Candidatus* Nitrosopumilus sp. HCA1 (KF957663.1)	96%	0	0	0	0	1
**43070**	LT897457	*Candidatus* Nitrosopelagicus brevis strain CN25 (CP007026.1), *Nitrosopumilus* sp. DDS1 (KR737579.1), *Candidatus* Nitrosopumilus sp. NF5 (CP011070.1), *Candidatus* Nitrosopumilus sp. D3C (CP010868.1)	93%	0	0	0	0	1
**43084**	LT897458	*Candidatus* Nitrosopelagicus brevis strain CN25 (CP007026.1), *Nitrosopumilus* sp. DDS1 (KR737579.1), *Candidatus* Nitrosopumilus sp. NF5 (CP011070.1), *Candidatus* Nitrosopumilus sp. D3C (CP010868.1)	93%	0	0	0	0	1

**43114**	LT897459	*Candidatus* Nitrosopelagicus brevis strain CN25 (CP007026.1), *Nitrosopumilus* sp. DDS1 (KR737579.1), *Candidatus* Nitrosopumilus sp. NF5 (CP011070.1), *Candidatus* Nitrosopumilus sp. D3C (CP010868.1)	95%	1	0	0	0	0
**43406**	LT897460	*Candidatus* Nitrosopelagicus brevis strain CN25 (CP007026.1)	94%	0	1	0	0	0
**43445**	LT897461	*Candidatus* Nitrosopelagicus brevis strain CN25 (CP007026.1)	95%	0	1	0	0	0
**43550**	LT897462	*Nitrosopumilus* sp. HCA1 (KX950757.1)	87%	0	1	0	0	0
**43594**	LT897463	*Candidatus* Nitrosopelagicus brevis strain CN25 (CP007026.1)	98%	0	1	0	0	0
**43609**	LT897464	*Candidatus* Nitrosopelagicus brevis strain CN25 (CP007026.1), *Nitrosopumilus* sp. DDS1 (KR737579.1), *Candidatus* Nitrosopumilus sp. NF5 (CP011070.1), *Candidatus* Nitrosopumilus sp. D3C (CP010868.1)	98%	0	0	1	0	0
**43659**	LT897465	*Candidatus* Nitrosopelagicus brevis strain CN25 (CP007026.1)	95%	0	1	0	0	0
**43844**	LT897466	*Candidatus* Nitrosopelagicus brevis strain CN25 (CP007026.1)	96%	0	0	0	1	0
**43866**	LT897467	*Candidatus* Nitrosopelagicus brevis strain CN25 (CP007026.1)	94%	0	0	0	1	0
**43897**	LT897468	*Candidatus* Nitrosopelagicus brevis strain CN25 (CP007026.1)	96%	0	0	0	0	1
**43958**	LT897469	*Candidatus* Nitrosopelagicus brevis strain CN25 (CP007026.1)	95%	0	0	0	0	1
**44008**	LT897470	*Candidatus* Nitrosopelagicus brevis strain CN25 (CP007026.1), *Nitrosopumilus* sp. DDS1 (KR737579.1), *Candidatus* Nitrosopumilus sp. NF5 (CP011070.1), *Candidatus* Nitrosopumilus sp. D3C (CP010868.1)	94%	0	0	0	0	1
**44015**	LT897471	*Candidatus* Nitrosopelagicus brevis strain CN25 (CP007026.1)	93%	0	0	0	0	1
**44020**	LT897472	*Candidatus* Nitrosopelagicus brevis strain CN25 (CP007026.1), *Nitrosopumilus* sp. DDS1 (KR737579.1), *Candidatus* Nitrosopumilus sp. NF5 (CP011070.1), *Candidatus* Nitrosopumilus sp. D3C (CP010868.1)	95%	0	0	0	0	1
**44022**	LT897473	*Candidatus* Nitrosopelagicus brevis strain CN25 (CP007026.1)	91%	0	0	0	0	1
**44064**	LT897474	*Nitrosopumilus maritimus* strain NAOA6 (KT380502.1), *Candidatus* Nitrosopumilus sp. HCA1 (KF957663.1)	98%	0	1	0	0	0
**44074**	LT897475	*Nitrosopumilus* sp. DDS1 (KR737579.1), *Candidatus* Nitrosopumilus sp. NF5 (CP011070.1), *Candidatus* Nitrosopumilus sp. D3C (CP010868.1)	95%	0	0	0	1	0

**44176**	LT897476	*Nitrosopumilus maritimus* strain NAOA6 (KT380502.1), *Nitrosopumilus maritimus* strain SCM1 (NR_102913.1), *Candidatus* Nitrosopumilus koreensis strain AR1 (NR_102904.1), *Candidatus* Nitrosopumilus sp. AR2 (CP003843.1), *Candidatus* Nitrosopumilus sp. HCA1 (KF957663.1)	99%	0	4	0	0	0
**44177**	LT897477	*Candidatus* Nitrosopelagicus brevis strain CN25 (CP007026.1), *Nitrosopumilus* sp. DDS1 (KR737579.1), *Candidatus* Nitrosopumilus sp. NF5 (CP011070.1), *Candidatus* Nitrosopumilus sp. D3C (CP010868.1)	96%	0	1	0	1	3
**44178**	LT897478	*Candidatus* Nitrosopelagicus brevis strain CN25 (CP007026.1), *Nitrosopumilus* sp. DDS1 (KR737579.1), *Candidatus* Nitrosopumilus sp. NF5 (CP011070.1), *Candidatus* Nitrosopumilus sp. D3C (CP010868.1)	95%	0	0	0	0	1
**44180**	LT897479	*Nitrosopumilus* sp. DDS1 (KR737579.1), *Candidatus* Nitrosopumilus sp. NF5 (CP011070.1), *Candidatus* Nitrosopumilus sp. D3C (CP010868.1)	87%	0	1	0	0	0
**44192**	LT897480	*Candidatus* Nitrosopelagicus brevis strain CN25 (CP007026.1)	90%	0	1	0	0	0
