# Supplementary material for: Diversity and relative abundance of ammonia- and nitrite-oxidizing microorganisms in the offshore Namibian hypoxic zone
Source: PLoS One. 2019 May 21;14(5):e0217136. doi: 10.1371/journal.pone.0217136 (PMC6529010; doi:10.1371/journal.pone.0217136)
Supplement: S2 Table — (PDF) [file pone.0217136.s009.pdf]

Table S2. Top named or cultured representative(s) based on BLASTN searches and read counts matching the 2 OTUs related to the bacterial ammonia-oxidizing family Nitrosomodaceae.

**OTU**	**GenBank Accession no.**	**Top named or cultured representative(s)**	**%ID to match**	**10m**	**25m**	**100m**	**130m**	**250m**
**32223**	LT897481	*Nitrosospira briensis* C-128 (CP012371.1)	94%	0	1	0	0	0
**30423**	LT897482	*Nitrosospira briensis* isolate Nsp10	89%	0	41	10	36	0
