# Supplementary material for: Diversity and relative abundance of ammonia- and nitrite-oxidizing microorganisms in the offshore Namibian hypoxic zone
Source: PLoS One. 2019 May 21;14(5):e0217136. doi: 10.1371/journal.pone.0217136 (PMC6529010; doi:10.1371/journal.pone.0217136)
Supplement: S3 Table — (PDF) [file pone.0217136.s010.pdf]

Table S3. Top named or cultured representative(s) based on BLASTN searches and read counts matching the 295 OTUs related to the bacterial nitrite-oxidizing family Nitrospinaceae and 4 OTUs related to the bacterial nitrite-oxidizing phylum Nitrospirae.

OTU	GenBank Accession no.	Top named or cultured representative(s)	%ID to match	10m	25m	100m	130m	250m
**2300**	LT897483	*Nitrospina gracilis* (Atlantic Ocean isolate) (L35504.1), *Nitrospina gracilis* strain 3/211 (NR_104821.1)	92%	0	2	0	0	0
**2612**	LT897484	*Nitrospina gracilis* (Atlantic Ocean isolate) (L35504.1), *Nitrospina gracilis* strain 3/211 (NR_104821.1)	92%	158	1582	874	420	89
**2944**	LT897485	*Nitrospina* sp. enrichment culture clone Cb12, North Sea (KC706458.1)	91%	2	54	56	94	244
**3053**	LT897486	*Nitrospina gracilis* (Atlantic Ocean isolate) (L35504.1), *Nitrospina gracilis* strain 3/211 (NR_104821.1)	93%	15	92	54	28	7
**3145**	LT897487	*Nitrospina gracilis* (Atlantic Ocean isolate) (L35504.1)	94%	35	299	84	36	25
**3267**	LT897488	*Nitrospina* sp. enrichment culture clone Cb12, North Sea (KC706458.1)	93%	8	188	191	111	113
**3469**	LT897489	*Nitrospina* sp. enrichment culture clone Cb12, North Sea (KC706458.1)	94%	0	15	15	27	55
**4835**	LT897490	*Nitrospina gracilis* (Atlantic Ocean isolate) (L35504.1)	89%	1	2	0	0	0
**4944**	LT897491	*Nitrospina* sp. enrichment culture clone Cb12, North Sea (KC706458.1)	89%	13	88	34	27	7
**5738**	LT897492	*Nitrospina gracilis* strain 3/211 (NR_104821.1)	91%	2	4	1	0	0
**5787**	LT897493	*Nitrospina gracilis* strain 3/211 (NR_104821.1)	92%	30	402	178	88	24
**5943**	LT897494	*Nitrospina gracilis* (Atlantic Ocean isolate) (L35504.1), *Nitrospina gracilis* strain 3/211 (NR_104821.1)	91%	1	1	0	0	0
**5981**	LT897495	*Nitrospina gracilis* strain 3/211 (NR_104821.1)	89%	4	2	0	0	0
**7456**	LT897496	*Nitrospina gracilis* strain 3/211 (NR_104821.1)	92%	1	5	0	1	0
**7716**	LT897497	*Nitrospina gracilis* strain 3/211 (NR_104821.1)	91%	4	6	0	0	0
**7758**	LT897498	*Nitrospina gracilis* strain 3/211 (NR_104821.1)	90%	6	20	0	2	0
**8373**	LT897499	*Nitrospina gracilis* strain 3/211 (NR_104821.1)	90%	4	5	0	0	0

**10108**	LT897500	*Nitrospina gracilis* strain 3/211 (NR_104821.1)	88%	1	0	0	0	0
**10174**	LT897501	*Nitrospina gracilis* strain 3/211 (NR_104821.1)	87%	1	10	3	2	0
**10461**	LT897502	*Nitrospina* sp. enrichment culture clone Cb12, North Sea (KC706458.1)	90%	1	4	1	0	0
**11099**	LT897503	*Nitrospina gracilis* strain 3/211 (NR_104821.1)	91%	2	7	0	2	0
**13696**	LT897504	*Nitrospina* sp. enrichment culture clone Cb12, North Sea (KC706458.1)	91%	31	43	4	3	0
**14281**	LT897505	*Nitrospina* sp. enrichment culture clone Cb12, North Sea (KC706458.1)	93%	2	28	30	10	39
**15146**	LT897506	*Nitrospina gracilis* strain 3/211 (NR_104821.1), *Nitrospina gracilis* (Pacific Ocean isolate) (L35503.1)	92%	9	294	22	7	0
**15290**	LT897507	*Nitrospina* sp. enrichment culture clone Cb12, North Sea (KC706458.1)	94%	2	26	24	0	1
**15904**	LT897508	*Nitrospina* gracilis strain 3/211 (NR_104821.1)	92%	3	14	0	0	0
**16350**	LT897509	*Nitrospina gracilis* (Atlantic Ocean isolate) (L35504.1), *Nitrospina gracilis* strain 3/211 (NR_104821.1)	93%	1	21	0	1	0
**16949**	LT897510	*Nitrospina gracilis* (Atlantic Ocean isolate) (L35504.1), *Nitrospina gracilis* strain 3/211 (NR_104821.1)	89%	1	7	1	0	0
**17184**	LT897511	*Nitrospina gracilis* strain 3/211 (NR_104821.1)	85%	1	1	1	0	0
**17335**	LT897512	*Nitrospina gracilis* strain 3/211 (NR_104821.1)	90%	1	3	0	0	0
**17455**	LT897513	*Nitrospina gracilis* (Atlantic Ocean isolate) (L35504.1), *Nitrospina gracilis* strain 3/211 (NR_104821.1)	90%	1	4	1	1	1
**19055**	LT897514	*Nitrospina gracilis* strain 3/211 (NR_104821.1)	90%	1	2	0	0	0
**19292**	LT897515	*Nitrospina gracilis* strain 3/211 (NR_104821.1)	90%	1	1	1	1	0
**19700**	LT897516	*Nitrospina gracilis* strain 3/211 (NR_104821.1)	90%	1	0	0	0	0
**19741**	LT897517	*Nitrospina* sp. enrichment culture clone Cb12, North Sea (KC706458.1)	94%	2	10	17	0	0
**20687**	LT897518	*Nitrospina gracilis* (Atlantic Ocean isolate) (L35504.1), *Nitrospina gracilis* strain 3/211 (NR_104821.1)	90%	1	0	0	0	0
**20814**	LT897519	*Nitrospina gracilis* (Atlantic Ocean isolate) (L35504.1), *Nitrospina gracilis*	92%	0	67	3	3	0

		strain 3/211 (NR_104821.1)						
**20936**	LT897520	*Nitrospina gracilis* (Atlantic Ocean isolate) (L35504.1), *Nitrospina gracilis* strain 3/211 (NR_104821.1)	89%	0	32	17	2	7
**21004**	LT897521	*Nitrospina* sp. enrichment culture clone Cb12, North Sea (KC706458.1)	90%	0	1	0	2	1
**21114**	LT897522	*Nitrospina* sp. enrichment culture clone Cb12, North Sea (KC706458.1)	90%	0	59	1	1	2
**21254**	LT897523	*Nitrospina* sp. enrichment culture clone Cb12, North Sea (KC706458.1)	90%	0	1	0	0	0
**21293**	LT897524	*Nitrospina gracilis* (Atlantic Ocean isolate) (L35504.1), *Nitrospina gracilis* strain 3/211 (NR_104821.1)	92%	0	94	5	4	1
**21378**	LT897525	*Nitrospina* sp. enrichment culture clone Cb12, North Sea (KC706458.1)	89%	0	110	51	22	1
**21441**	LT897526	*Nitrospina gracilis* strain 3/211 (NR_104821.1), *Nitrospina gracilis* (Pacific Ocean isolate) (L35503.1)	90%	0	6	1	0	0
**21559**	LT897527	*Nitrospina gracilis* (Atlantic Ocean isolate) (L35504.1), *Nitrospina gracilis* strain 3/211 (NR_104821.1)	92%	0	441	200	29	8
**21948**	LT897528	*Nitrospina gracilis* (Atlantic Ocean isolate) (L35504.1), *Nitrospina gracilis* strain 3/211 (NR_104821.1)	92%	0	23	6	10	12
**21953**	LT897529	*Nitrospina gracilis* (Atlantic Ocean isolate) (L35504.1), *Nitrospina gracilis* strain 3/211 (NR_104821.1)	92%	0	26	7	5	0
**22024**	LT897530	*Nitrospina gracilis* strain 3/211 (NR_104821.1)	88%	0	1	0	1	0
**22151**	LT897531	*Nitrospina gracilis* strain 3/211 (NR_104821.1), *Nitrospina gracilis* (Atlantic Ocean isolate) (L35504.1), *Nitrospina gracilis* (Pacific Ocean isolate) (L35503.1)	88%	0	2	0	0	0
**22176**	LT897532	*Nitrospina gracilis* (Atlantic Ocean isolate) (L35504.1), *Nitrospina gracilis* strain 3/211 (NR_104821.1)	90%	0	1	0	0	0
**22179**	LT897533	*Nitrospina* sp. enrichment culture clone Cb12, North Sea (KC706458.1)	94%	0	218	117	91	92
**22307**	LT897534	*Nitrospina gracilis* strain 3/211 (NR_104821.1), *Nitrospina gracilis* (Atlantic Ocean isolate) (L35504.1)	88%	0	5	0	0	0
**22403**	LT897535	*Nitrospina gracilis strain* 3/211 (NR_104821.1), *Nitrospina gracilis* (Atlantic Ocean isolate) (L35504.1)	92%	0	22	1	1	0

**22710**	LT897536	*Nitrospira* sp. enrichment culture clone LD3 (|KJ598611.1)	98%	0	2	0	1	0
**22733**	LT897537	*Nitrospina* sp. enrichment culture clone Cb12, North Sea (KC706458.1)	88%	0	6	1	0	0
**22798**	LT897538	*Nitrospina* gracilis strain 3/211 (NR_104821.1), *Nitrospina gracilis* (Atlantic Ocean isolate) (L35504.1)	92%	0	383	8	5	1
**22807**	LT897539	*Nitrospina* sp. enrichment culture clone Cb12, North Sea (KC706458.1)	88%	0	5	0	0	0
**22813**	LT897540	*Nitrospina gracilis* strain 3/211 (NR_104821.1), *Nitrospina gracilis* (Atlantic Ocean isolate) (L35504.1)	89%	0	1	0	0	0
**22898**	LT897541	*Nitrospina* sp. enrichment culture clone Cb12, North Sea (KC706458.1)	88%	0	1	0	0	0
**22913**	LT897542	*Nitrospina gracilis* strain 3/211 (NR_104821.1), *Nitrospina gracilis* (Atlantic Ocean isolate) (L35504.1)	91%	0	15	3	1	2
**23017**	LT897543	*Nitrospina gracilis* strain 3/211 (NR_104821.1)	93%	0	15	12	14	7
**23810**	LT897544	*Nitrospina* sp. enrichment culture clone Cb12, North Sea (KC706458.1)	87%	0	2	0	1	0
**23886**	LT897545	*Nitrospina gracilis* strain 3/211 (NR_104821.1), *Nitrospina gracilis* (Atlantic Ocean isolate) (L35504.1	88%	0	1	0	0	0
**23975**	LT897546	*Nitrospina gracilis* strain 3/211 (NR_104821.1)	90%	0	1	0	0	0
**24232**	LT897547	*Nitrospina gracilis* strain 3/211 (NR_104821.1), *Nitrospina gracilis* (Atlantic Ocean isolate) (L35504.1	91%	0	4	1	0	1
**24488**	LT897548	*Nitrospina gracilis* strain 3/211 (NR_104821.1), *Nitrospina gracilis* (Atlantic Ocean isolate) (L35504.1	92%	0	385	150	3	0
**24503**	LT897549	*Nitrospina gracilis* strain 3/211 (NR_104821.1)	90%	0	2	0	0	0
**24645**	LT897550	*Nitrospina gracilis* strain 3/211 (NR_104821.1), *Nitrospina gracilis* (Atlantic Ocean isolate) (L35504.1)	90%	0	3	1	0	0
**24732**	LT897551	*Nitrospina* sp. enrichment culture clone Cb12, North Sea (KC706458.1)	89%	0	1	0	0	0
**24965**	LT897552	*Nitrospina* sp. enrichment culture clone Cb12, North Sea (KC706458.1)	93%	0	4	3	6	7
**25168**	LT897553	*Nitrospina gracilis* strain 3/211 (NR_104821.1), *Nitrospina gracilis* (Atlantic Ocean isolate) (L35504.1)	93%	0	32	5	2	1
**25487**	LT897554	*Nitrospina gracilis* strain 3/211	88%	0	2	1	0	0

		(NR_104821.1), *Nitrospina gracilis* (Atlantic Ocean isolate) (L35504.1)						
**25645**	LT897555	*Nitrospina* sp. enrichment culture clone Cb12, North Sea (KC706458.1)	88%	0	615	14	14	1
**25878**	LT897556	*Nitrospina gracilis* strain 3/211 (NR_104821.1), *Nitrospina gracilis* (Atlantic Ocean isolate) (L35504.1)	92%	0	1	0	1	0
**25903**	LT897557	*Nitrospina* sp. enrichment culture clone Cb12, North Sea (KC706458.1)	94%	0	1	0	0	0
**26142**	LT897558	*Nitrospina gracilis* strain 3/211 (NR_104821.1)	93%	0	3	2	2	3
**26326**	LT897559	*Nitrospina gracilis* strain 3/211 (NR_104821.1), *Nitrospina gracilis* (Atlantic Ocean isolate) (L35504.1)	92%	0	6	4	1	0
**26481**	LT897560	*Nitrospina gracilis* strain 3/211 (NR_104821.1), *Nitrospina gracilis* (Atlantic Ocean isolate) (L35504.1)	88%	0	1	0	0	0
**26591**	LT897561	*Nitrospina gracilis* strain 3/211 (NR_104821.1)	93%	0	2	0	0	0
**26647**	LT897562	*Nitrospina gracilis* strain 3/211 (NR_104821.1), *Nitrospina gracilis* (Atlantic Ocean isolate) (L35504.1)	90%	0	1	0	0	0
**26670**	LT897563	*Nitrospina gracilis* (Atlantic Ocean isolate) (L35504.1)	90%	0	9	2	1	0
**26858**	LT897564	*Nitrospina gracilis* strain 3/211 (NR_104821.1)	90%	0	1	0	0	0
**27139**	LT897565	*Nitrospina gracilis* strain 3/211 (NR_104821.1)	88%	0	1	1	0	1
**27236**	LT897566	*Nitrospina gracilis* strain 3/211 (NR_104821.1), *Nitrospina gracilis* (Pacific Ocean isolate) (L35503.1)	92%	0	38	6	4	0
**27676**	LT897567	*Nitrospina gracilis* strain 3/211 (NR_104821.1)	91%	0	1	0	0	0
**27783**	LT897568	*Nitrospina* sp. enrichment culture clone Cb12, North Sea (KC706458.1)	89%	0	1	0	0	0
**28025**	LT897569	*Nitrospina* sp. enrichment culture clone Cb12, North Sea (KC706458.1)	93%	0	89	2	2	0
**28178**	LT897570	*Nitrospina gracilis* strain 3/211 (NR_104821.1)	87%	0	1	0	0	0
**28610**	LT897571	*Nitrospina gracilis* strain 3/211 (NR_104821.1), *Nitrospina gracilis* (Atlantic Ocean isolate) (L35504.1)	88%	0	1	0	1	0
**28705**	LT897572	*Nitrospina gracilis* strain 3/211 (NR_104821.1)	89%	0	1	0	0	0
**28741**	LT897573	*Nitrospina gracilis* strain 3/211	90%	0	1	0	0	0

		(NR_104821.1), *Nitrospina gracilis* (Atlantic Ocean isolate) (L35504.1)						
**29113**	LT897574	*Nitrospina* sp. enrichment culture clone Cb12, North Sea (KC706458.1)	87%	0	1	0	0	0
**29130**	LT897575	*Nitrospina gracilis* strain 3/211 (NR_104821.1)	90%	0	3	0	0	0
**29387**	LT897576	*Nitrospina gracilis* strain 3/211 (NR_104821.1), *Nitrospina gracilis* (Atlantic Ocean isolate) (L35504.1)	91%	0	9	4	2	0
**29617**	LT897577	*Nitrospina gracilis* strain 3/211 (NR_104821.1)	87%	0	1	0	0	0
**29783**	LT897578	*Nitrospina gracilis* strain 3/211 (NR_104821.1), *Nitrospina gracilis* (Atlantic Ocean isolate) (L35504.1)	90%	0	1	1	1	0
**29816**	LT897579	*Nitrospina* sp. enrichment culture clone Cb12, North Sea (KC706458.1)	90%	0	1	0	0	0
**29902**	LT897580	*Nitrospina* sp. enrichment culture clone Cb12, North Sea (KC706458.1)	89%	0	1	0	1	0
**30021**	LT897581	*Nitrospina gracilis* strain 3/211 (NR_104821.1), *Nitrospina gracilis* (Atlantic Ocean isolate) (L35504.1), *Nitrospina gracilis* (Pacific Ocean isolate) (L35503.1)	87%	0	2	0	0	0
**30052**	LT897582	*Nitrospina* sp. enrichment culture clone Cb12, North Sea (KC706458.1)	90%	0	2	1	0	0
**30072**	LT897583	*Nitrospina gracilis* strain 3/211 (NR_104821.1), *Nitrospina gracilis* (Pacific Ocean isolate) (L35503.1)	92%	0	287	241	123	36
**30118**	LT897584	*Nitrospina gracilis* strain 3/211 (NR_104821.1)	90%	0	1	0	0	0
**30189**	LT897585	*Nitrospina gracilis* strain 3/211 (NR_104821.1)	92%	0	3	2	1	0
**30286**	LT897586	*Nitrospina gracilis* (Atlantic Ocean isolate) (L35504.1),	93%	0	21	2	1	0
**30406**	LT897587	*Nitrospina gracilis* strain 3/211 (NR_104821.1), *Nitrospina gracilis* (Atlantic Ocean isolate) (L35504.1)	92%	0	230	104	33	6
**30460**	LT897588	*Nitrospina gracilis* strain 3/211 (NR_104821.1)	89%	0	1	0	0	0
**30576**	LT897589	*Nitrospina gracilis* strain 3/211 (NR_104821.1), *Nitrospina gracilis* (Atlantic Ocean isolate) (L35504.1)	92%	0	87	28	11	4
**31039**	LT897590	*Nitrospina gracilis* strain 3/211 (NR_104821.1), *Nitrospina gracilis* (Atlantic Ocean isolate) (L35504.1)	92%	0	1	0	0	0

**31507**	LT897591	*Nitrospina gracilis* strain 3/211 (NR_104821.1), *Nitrospina gracilis* (Atlantic Ocean isolate) (L35504.1)	89%	0	111	136	16	4
**31598**	LT897592	*Nitrospina gracilis* (Atlantic Ocean isolate) (L35504.1)	94%	0	28	29	16	1
**31644**	LT897593	*Nitrospina gracilis* strain 3/211 (NR_104821.1), *Nitrospina gracilis* (Atlantic Ocean isolate) (L35504.1)	89%	0	1	0	0	0
**31858**	LT897594	*Nitrospina gracilis* strain 3/211 (NR_104821.1), *Nitrospina gracilis* (Atlantic Ocean isolate) (L35504.1)	93%	0	408	436	4	2
**31936**	LT897595	*Nitrospina gracilis* strain 3/211 (NR_104821.1), *Nitrospina gracilis* (Atlantic Ocean isolate) (L35504.1)	89%	0	1	0	0	0
**32053**	LT897596	*Nitrospina gracilis* strain 3/211 (NR_104821.1), *Nitrospina gracilis* (Atlantic Ocean isolate) (L35504.1)	92%	0	1	0	0	0
**32101**	LT897597	*Nitrospina gracilis* strain 3/211 (NR_104821.1)	87%	0	1	0	0	0
**32105**	LT897598	*Nitrospina gracilis* strain 3/211 (NR_104821.1), *Nitrospina gracilis* (Atlantic Ocean isolate) (L35504.1)	90%	0	2	1	3	0
**32166**	LT897599	*Nitrospina gracilis* strain 3/211 (NR_104821.1)	87%	0	2	0	0	0
**32486**	LT897600	*Nitrospina gracilis* strain 3/211 (NR_104821.1)	92%	0	0	1	0	0
**32509**	LT897601	*Nitrospina gracilis* strain 3/211 (NR_104821.1), *Nitrospina gracilis* (Atlantic Ocean isolate) (L35504.1)	92%	0	6	8	6	8
**32521**	LT897602	*Nitrospina gracilis* strain 3/211 (NR_104821.1), *Nitrospina gracilis* (Atlantic Ocean isolate) (L35504.1)	90%	0	0	1	6	4
**32905**	LT897603	*Nitrospina* sp. enrichment culture clone Cb12, North Sea (KC706458.1)	94%	0	30	31	11	17
**32910**	LT897604	*Nitrospina* sp. enrichment culture clone Cb12, North Sea (KC706458.1)	92%	0	31	32	20	10
**32947**	LT897605	*Nitrospina* sp. enrichment culture clone Cb12, North Sea (KC706458.1)	90%	0	0	1	0	0
**32958**	LT897606	*Nitrospina* sp. enrichment culture clone Cb12, North Sea (KC706458.1)	91%	0	0	1	0	0
**33016**	LT897607	*Nitrospina* sp. enrichment culture clone Cb12, North Sea (KC706458.1)	88%	0	0	1	0	0
**33192**	LT897608	*Nitrospina gracilis* strain 3/211 (NR_104821.1)	87%	0	0	1	0	0

**33307**	LT897609	*Nitrospina gracilis* strain 3/211 (NR_104821.1)	91%	0	2	2	0	0
**33625**	LT897610	*Nitrospina gracilis* strain 3/211 (NR_104821.1), *Nitrospina gracilis* (Atlantic Ocean isolate) (L35504.1)	88%	0	1	1	0	0
**33691**	LT897611	*Nitrospina* sp. enrichment culture clone Cb12, North Sea (KC706458.1)	94%	0	6	6	3	0
**33768**	LT897612	*Nitrospina* sp. enrichment culture clone Cb12, North Sea (KC706458.1)	88%	0	116	116	8	2
**33781**	LT897613	*Nitrospina* sp. enrichment culture clone Cb12, North Sea (KC706458.1)	89%	0	1	1	0	0
**33795**	LT897614	*Nitrospina* sp. enrichment culture clone Cb12, North Sea (KC706458.1)	88%	0	1	1	0	0
**33818**	LT897615	*Nitrospina gracilis* strain 3/211 (NR_104821.1)	91%	0	6	6	0	1
**33841**	LT897616	*Nitrospina gracilis* strain 3/211 (NR_104821.1), *Nitrospina gracilis* (Atlantic Ocean isolate) (L35504.1)	89%	0	88	88	73	237
**33865**	LT897617	*Nitrospina gracilis* strain 3/211 (NR_104821.1)	90%	0	1	1	3	0
**33909**	LT897618	*Nitrospina* sp. enrichment culture clone Cb12, North Sea (KC706458.1)	90%	0	1	1	1	0
**33919**	LT897619	*Nitrospina gracilis* strain 3/211 (NR_104821.1), *Nitrospina gracilis* (Atlantic Ocean isolate) (L35504.1)	91%	0	18	18	26	39
**33948**	LT897620	*Nitrospina gracilis* strain 3/211 (NR_104821.1), *Nitrospina gracilis* (Atlantic Ocean isolate) (L35504.1)	91%	0	72	72	78	0
**33974**	LT897621	*Nitrospina gracilis* strain 3/211 (NR_104821.1), *Nitrospina gracilis* (Atlantic Ocean isolate) (L35504.1)	91%	0	54	54	60	49
**34046**	LT897622	*Nitrospina gracilis* strain 3/211 (NR_104821.1), *Nitrospina gracilis* (Atlantic Ocean isolate) (L35504.1)	89%	0	199	199	25	10
**34125**	LT897623	*Nitrospina gracilis* strain 3/211 (NR_104821.1), *Nitrospina gracilis* (Atlantic Ocean isolate) (L35504.1)	92%	0	2	2	5	2
**34137**	LT897624	*Nitrospina gracilis* strain 3/211 (NR_104821.1), *Nitrospina gracilis* (Atlantic Ocean isolate) (L35504.1)	92%	0	196	196	55	3
**34138**	LT897625	*Nitrospina* sp. enrichment culture clone Cb12, North Sea (KC706458.1)	90%	0	1	1	2	1
**34145**	LT897626	*Nitrospina* sp. enrichment culture clone Cb12, North Sea (KC706458.1)	91%	0	8	8	27	42

**34155**	LT900516	*Nitrospira* sp. enrichment culture clone M1-9 (HQ686083.1)	92%	0	2	0	1	7
**34163**	LT897627	*Nitrospina gracilis* strain 3/211 (NR_104821.1), *Nitrospina gracilis* (Atlantic Ocean isolate) (L35504.1)	91%	0	633	2	6	1
**34301**	LT897628	*Nitrospina gracilis* strain 3/211 (NR_104821.1), *Nitrospina gracilis* (Atlantic Ocean isolate) (L35504.1)	91%	0	2	633	0	0
**34435**	LT897629	*Nitrospina* sp. enrichment culture clone Cb12, North Sea (KC706458.1)	91%	0	21	2	27	6
**34493**	LT897630	*Nitrospina* sp. enrichment culture clone Cb12, North Sea (KC706458.1)	94%	0	55	21	34	11
**34551**	LT897631	*Nitrospina* sp. enrichment culture clone Cb12, North Sea (KC706458.1)	93%	0	1	55	4	1
**34576**	LT897632	*Nitrospina* sp. enrichment culture clone Cb12, North Sea (KC706458.1)	93%	0	35	1	45	12
**34577**	LT897633	*Nitrospina gracilis* strain 3/211 (NR_104821.1), *Nitrospina gracilis* (Atlantic Ocean isolate) (L35504.1)	88%	0	1	36	0	0
**34581**	LT897634	*Nitrospina gracilis* strain 3/211 (NR_104821.1), *Nitrospina gracilis* (Atlantic Ocean isolate) (L35504.1)	88%	0	1	1	0	0
**34607**	LT897635	*Nitrospina gracilis* strain 3/211 (NR_104821.1), *Nitrospina gracilis* (Atlantic Ocean isolate) (L35504.1), *Nitrospina gracilis* (Pacific Ocean isolate) (L35503.1)	88%	0	1	1	2	0
**34680**	LT897636	*Nitrospina* sp. enrichment culture clone Cb12, North Sea (KC706458.1)	88%	0	1	1	0	0
**34718**	LT897637	*Nitrospina gracilis* strain 3/211 (NR_104821.1), *Nitrospina gracilis* (Atlantic Ocean isolate) (L35504.1)	91%	0	555	1	550	128
**34724**	LT897638	*Nitrospina gracilis* strain 3/211 (NR_104821.1), *Nitrospina gracilis* (Atlantic Ocean isolate) (L35504.1)	90%	0	4	553	3	0
**35150**	LT897639	*Nitrospina* sp. enrichment culture clone Cb12, North Sea (KC706458.1)	92%	0	1	4	4	4
**35201**	LT897640	*Nitrospina gracilis* strain 3/211 (NR_104821.1), *Nitrospina gracilis* (Atlantic Ocean isolate) (L35504.1)	93%	1	14	1	597	40
**35234**	LT897641	*Nitrospina gracilis* strain 3/211 (NR_104821.1), *Nitrospina gracilis* (Atlantic Ocean isolate) (L35504.1)	89%	0	1	8	300	64
**35240**	LT897642	*Nitrospina* sp. enrichment culture clone Cb12, North Sea (KC706458.1)	94%	0	1	1	122	7

**35260**	LT897643	*Nitrospina gracilis* strain 3/211 (NR_104821.1), *Nitrospina gracilis* (Atlantic Ocean isolate) (L35504.1)	92%	0	1	1	5	3
**35364**	LT897644	*Nitrospina gracilis* strain 3/211 (NR_104821.1)	88%	0	0	2	4	11
**35438**	LT897645	*Nitrospin*a sp. enrichment culture clone Cb12, North Sea (KC706458.1)	90%	0	0	0	1	0
**35538**	LT897646	*Nitrospina* sp. enrichment culture clone Cb12, North Sea (KC706458.1)	94%	0	0	0	206	25
**35600**	LT897647	*Nitrospina gracilis* strain 3/211 (NR_104821.1), *Nitrospina gracilis* (Atlantic Ocean isolate) (L35504.1)	89%	0	0	0	2	0
**35686**	LT897648	*Nitrospina* sp. enrichment culture clone Cb12, North Sea (KC706458.1)	91%	0	0	0	1	0
**35692**	LT897649	*Nitrospina* sp. enrichment culture clone Cb12, North Sea (KC706458.1)	89%	0	0	0	3	0
**35738**	LT897650	*Nitrospina* sp. enrichment culture clone Cb12, North Sea (KC706458.1)	94%	0	0	0	1	0
**35824**	LT897651	*Nitrospina* sp. enrichment culture clone Cb12, North Sea (KC706458.1)	92%	0	0	0	29	105
**35849**	LT897652	*Nitrospina gracilis* strain 3/211 (NR_104821.1), *Nitrospina gracilis* (Atlantic Ocean isolate) (L35504.1)	92%	0	0	0	69	1
**35972**	LT897653	*Nitrospina* sp. enrichment culture clone Cb12, North Sea (KC706458.1)	86%	0	0	0	94	0
**36148**	LT897654	*Nitrospina gracilis* strain 3/211 (NR_104821.1), *Nitrospina gracilis* (Atlantic Ocean isolate) (L35504.1)	91%	0	0	0	2	0
**36221**	LT897655	*Nitrospina gracilis* strain 3/211 (NR_104821.1), *Nitrospina gracilis* (Atlantic Ocean isolate) (L35504.1)	92%	0	0	0	32	21
**36355**	LT897656	*Nitrospina* sp. enrichment culture clone Cb12, North Sea (KC706458.1)	94%	0	0	0	1	1
**36547**	LT897657	*Nitrospina* sp. enrichment culture clone Cb12, North Sea (KC706458.1)	94%	0	1	0	261	61
**36592**	LT897658	*Nitrospina* sp. enrichment culture clone Cb12, North Sea (KC706458.1)	92%	0	0	0	21	39
**36624**	LT897659	*Nitrospina gracilis* strain 3/211 (NR_104821.1)	87%	0	0	0	3	0
**36636**	LT897660	*Nitrospina gracilis* strain 3/211 (NR_104821.1)	93%	0	0	0	7	0
**36671**	LT897661	*Nitrospina gracilis* strain 3/211 (NR_104821.1)	91%	0	0	0	1	0

**36681**	LT897662	*Nitrospina gracilis* strain 3/211 (NR_104821.1), *Nitrospina gracilis* (Atlantic Ocean isolate) (L35504.1)	90%	0	0	0	1	0
**36799**	LT897663	*Nitrospina gracilis* (Atlantic Ocean isolate) (L35504.1)	91%	0	0	0	32	70
**36831**	LT897664	*Nitrospina gracilis* strain 3/211 (NR_104821.1), *Nitrospina gracilis* (Atlantic Ocean isolate) (L35504.1)	92%	0	0	0	9	8
**36836**	LT897665	*Nitrospina gracilis* strain 3/211 (NR_104821.1), *Nitrospina gracilis* (Atlantic Ocean isolate) (L35504.1)	92%	0	0	0	2	2
**36877**	LT897666	*Nitrospina gracilis* strain 3/211 (NR_104821.1), *Nitrospina gracilis* (Atlantic Ocean isolate) (L35504.1)	93%	0	1	0	49	32
**36916**	LT897667	*Nitrospina gracilis* strain 3/211 (NR_104821.1)	90%	0	0	0	1	1
**36924**	LT897668	*Nitrospina gracilis* strain 3/211 (NR_104821.1), *Nitrospina gracilis* (Atlantic Ocean isolate) (L35504.1)	92%	0	2	0	167	322
**36948**	LT897669	*Nitrospina gracilis* strain 3/211 (NR_104821.1), *Nitrospina gracilis* (Atlantic Ocean isolate) (L35504.1)	91%	0	0	1	3	20
**36974**	LT897670	*Nitrospina* sp. enrichment culture clone Cb12, North Sea (KC706458.1)	90%	0	0	0	1	1
**37043**	LT897671	*Nitrospina gracilis* strain 3/211 (NR_104821.1)	93%	0	0	0	1	0
**37080**	LT897672	*Nitrospina gracilis* strain 3/211 (NR_104821.1), *Nitrospina gracilis* (Atlantic Ocean isolate) (L35504.1)	90%	0	0	0	19	38
**37191**	LT897673	*Nitrospina* sp. enrichment culture clone Cb12, North Sea (KC706458.1)	91%	0	0	0	2	0
**37236**	LT897674	*Nitrospina gracilis* strain 3/211 (NR_104821.1), *Nitrospina gracilis* (Atlantic Ocean isolate) (L35504.1)	92%	0	0	0	47	5
**37238**	LT897675	*Nitrospina gracilis* strain 3/211 (NR_104821.1), *Nitrospina gracilis* (Atlantic Ocean isolate) (L35504.1)	89%	0	0	0	1	1
**37243**	LT897676	*Nitrospina gracilis* strain 3/211 (NR_104821.1), *Nitrospina gracilis* (Atlantic Ocean isolate) (L35504.1)	90%	0	0	0	1	0
**37295**	LT897677	*Nitrospina gracilis* strain 3/211 (NR_104821.1), *Nitrospina gracilis* (Atlantic Ocean isolate) (L35504.1)	90%	0	0	0	2	0
**37310**	LT897678	*Nitrospina gracilis* strain 3/211 (NR_104821.1), *Nitrospina gracilis* (Atlantic Ocean isolate) (L35504.1)	90%	0	0	0	21	1

**37361**	LT897679	*Nitrospina gracilis* strain 3/211 (NR_104821.1), *Nitrospina gracilis* (Atlantic Ocean isolate) (L35504.1)	92%	0	0	0	1	0
**37423**	LT897680	*Nitrospina* sp. enrichment culture clone Cb12, North Sea (KC706458.1)	93%	0	0	0	1	1
**37468**	LT897681	*Nitrospina gracilis* strain 3/211 (NR_104821.1), *Nitrospina gracilis* (Atlantic Ocean isolate) (L35504.1)	89%	0	0	0	1	0
**37484**	LT897682	*Nitrospina gracilis* strain 3/211 (NR_104821.1), *Nitrospina gracilis* (Atlantic Ocean isolate) (L35504.1)	91%	0	7	0	124	25
**37503**	LT897683	*Nitrospina gracilis* strain 3/211 (NR_104821.1), *Nitrospina gracilis* (Atlantic Ocean isolate) (L35504.1)	92%	0	0	5	1	1
**37544**	LT897684	*Nitrospina gracilis* strain 3/211 (NR_104821.1)	90%	0	0	0	16	3
**37616**	LT897685	*Nitrospina gracilis* strain 3/211 (NR_104821.1), *Nitrospina gracilis* (Atlantic Ocean isolate) (L35504.1)	88%	0	0	0	1	0
**37619**	LT897686	*Nitrospina* sp. enrichment culture clone Cb12, North Sea (KC706458.1)	89%	0	0	0	1	0
**37704**	LT897687	*Nitrospina gracilis* (Atlantic Ocean isolate) (L35504.1)	91%	0	0	0	1	3
**37746**	LT897688	*Nitrospina gracilis* strain 3/211 (NR_104821.1), *Nitrospina gracilis* (Atlantic Ocean isolate) (L35504.1)	91%	0	2	0	86	123
**37791**	LT897689	*Nitrospina* sp. enrichment culture clone Cb12, North Sea (KC706458.1)	89%	0	0	1	1	0
**37834**	LT897690	*Nitrospina* sp. enrichment culture clone Cb12, North Sea (KC706458.1)	92%	0	0	0	1	0
**37843**	LT897691	*Nitrospina* sp. enrichment culture clone Cb12, North Sea (KC706458.1)	91%	0	0	0	1	0
**37975**	LT897692	*Nitrospina gracilis* strain 3/211 (NR_104821.1), *Nitrospina gracilis* (Atlantic Ocean isolate) (L35504.1)	88%	0	0	0	1	0
**38020**	LT897693	*Nitrospina gracilis* strain 3/211 (NR_104821.1), *Nitrospina gracilis* (Atlantic Ocean isolate) (L35504.1)	90%	0	0	0	1	0
**38026**	LT897694	*Nitrospina gracilis* strain 3/211 (NR_104821.1), *Nitrospina gracilis* (Atlantic Ocean isolate) (L35504.1)	93%	0	0	0	1	0
**38084**	LT897695	*Nitrospina* sp. enrichment culture clone Cb12, North Sea (KC706458.1)	94%	0	0	0	46	81
**38114**	LT897696	*Nitrospina gracilis* strain 3/211 (NR_104821.1), *Nitrospina gracilis* (Atlantic Ocean isolate) (L35504.1)	90%	0	0	0	125	187

**38171**	LT897697	*Nitrospina gracilis* strain 3/211 (NR_104821.1), *Nitrospina gracilis* (Atlantic Ocean isolate) (L35504.1), *Nitrospina gracilis* (Pacific Ocean isolate) (L35503.1)	92%	0	0	0	4	1
**38267**	LT897698	*Nitrospina* sp. enrichment culture clone Cb12, North Sea (KC706458.1)	88%	0	0	0	1	0
**38328**	LT897699	*Nitrospina gracilis* strain 3/211 (NR_104821.1), *Nitrospina gracilis* (Atlantic Ocean isolate) (L35504.1)	92%	0	0	0	5	1
**38353**	LT897700	*Nitrospina* sp. enrichment culture clone Cb12, North Sea (KC706458.1)	93%	0	0	0	15	3
**38382**	LT897701	*Nitrospina* sp. enrichment culture clone Cb12, North Sea (KC706458.1)	94%	0	0	0	14	17
**38418**	LT897702	*Nitrospina* sp. enrichment culture clone Cb12, North Sea (KC706458.1)	94%	0	0	0	1	34
**38451**	LT897703	*Nitrospina* sp. enrichment culture clone Cb12, North Sea (KC706458.1)	88%	0	0	0	1	0
**38456**	LT897704	*Nitrospina* sp. enrichment culture clone Cb12, North Sea (KC706458.1)	93%	0	0	0	1	3
**38562**	LT897705	*Nitrospina* sp. enrichment culture clone Cb12, North Sea (KC706458.1)	88%	0	0	0	1	0
**38566**	LT897706	*Nitrospina gracilis* strain 3/211 (NR_104821.1), *Nitrospina gracilis* (Atlantic Ocean isolate) (L35504.1)	90%	0	0	0	1	12
**38614**	LT897707	*Nitrospina gracilis* strain 3/211 (NR_104821.1), *Nitrospina gracilis* (Atlantic Ocean isolate) (L35504.1)	92%	0	0	0	1	1
**38629**	LT897708	*Nitrospina gracilis* strain 3/211 (NR_104821.1), *Nitrospina gracilis* (Atlantic Ocean isolate) (L35504.1)	88%	0	0	0	1	0
**38648**	LT897709	*Nitrospina gracilis* strain 3/211 (NR_104821.1), *Nitrospina gracilis* (Atlantic Ocean isolate) (L35504.1)	90%	0	0	0	1	3
**38761**	LT897710	*Nitrospina gracilis* strain 3/211 (NR_104821.1)	90%	0	3	0	4	792
**38795**	LT897711	*Nitrospina gracilis* strain 3/211 (NR_104821.1), *Nitrospina gracilis* (Atlantic Ocean isolate) (L35504.1)	91%	0	0	3	1	0
**38853**	LT897712	*Nitrospina gracilis* strain 3/211 (NR_104821.1), *Nitrospina gracilis* (Atlantic Ocean isolate) (L35504.1)	90%	0	0	0	1	3
**38862**	LT897713	*Nitrospina gracilis* strain 3/211 (NR_104821.1), *Nitrospina gracilis* (Atlantic Ocean isolate) (L35504.1)	86%	0	0	0	1	0

**38891**	LT897714	*Nitrospina gracilis* strain 3/211 (NR_104821.1), *Nitrospina gracilis* (Atlantic Ocean isolate) (L35504.1)	92%	0	1	0	2	31
**39106**	LT897715	*Nitrospina* sp. enrichment culture clone Cb12, North Sea (KC706458.1)	94%	0	0	0	3	936
**39208**	LT897716	*Nitrospina* sp. enrichment culture clone Cb12, North Sea (KC706458.1)	93%	0	0	0	0	38
**39213**	LT897717	*Nitrospina gracilis* (Pacific Ocean isolate) (L35503.1)	87%	0	0	0	0	5
**39263**	LT897718	*Nitrospina gracilis* (Pacific Ocean isolate) (L35503.1)	88%	0	0	0	0	42
**39317**	LT897719	*Nitrospina gracilis* (Pacific Ocean isolate) (L35503.1)	88%	0	1	0	1	359
**39325**	LT897720	*Nitrospina gracilis* strain 3/211 (NR_104821.1), *Nitrospina gracilis* (Atlantic Ocean isolate) (L35504.1)	92%	0	0	1	0	58
**39346**	LT897721	*Nitrospina gracilis* (Pacific Ocean isolate) (L35503.1)	88%	0	0	0	0	5
**39444**	LT897722	*Nitrospina gracilis* strain 3/211 (NR_104821.1)	88%	0	0	0	0	2
**39473**	LT897723	*Nitrospina gracilis* strain 3/211 (NR_104821.1), *Nitrospina gracilis* (Atlantic Ocean isolate) (L35504.1), *Nitrospina gracilis* (Pacific Ocean isolate) (L35503.1)	92%	0	0	0	0	1
**39524**	LT897724	*Nitrospina gracilis* strain 3/211 (NR_104821.1)	94%	0	0	0	0	2
**39558**	LT897725	*Nitrospina gracilis* strain 3/211 (NR_104821.1), *Nitrospina gracilis* (Pacific Ocean isolate) (L35503.1)	88%	0	0	0	0	1
**39619**	LT897726	*Nitrospina gracilis* strain 3/211 (NR_104821.1), *Nitrospina gracilis* (Atlantic Ocean isolate) (L35504.1)	92%	0	0	0	0	7
**40090**	LT897727	*Nitrospira* sp. enrichment culture clone LD3 (KJ598611.1)	92%	0	0	0	0	1
**40093**	LT897728	*Nitrospina gracilis* (Pacific Ocean isolate) (L35503.1)	88%	0	1	0	1	121
**40113**	LT897729	*Nitrospina gracilis* (Pacific Ocean isolate) (L35503.1)	88%	0	0	1	0	3
**40142**	LT897730	*Nitrospina gracilis* (Pacific Ocean isolate) (L35503.1)	87%	0	0	0	0	1
**40245**	LT897731	*Nitrospina gracilis* strain 3/211 (NR_104821.1), *Nitrospina gracilis* (Atlantic Ocean isolate) (L35504.1)	90%	0	1	0	0	23
**40414**	LT897732	*Nitrospina gracilis* strain 3/211 (NR_104821.1), *Nitrospina gracilis*	92%	0	3	1	5	523

		(Atlantic Ocean isolate) (L35504.1)						
**40426**	LT897733	*Nitrospina* sp. enrichment culture clone Cb12, North Sea (KC706458.1)	93%	0	1	3	0	3
**40436**	LT897734	*Nitrospina gracilis* (Atlantic Ocean isolate) (L35504.1)	90%	0	0	1	0	1
**40496**	LT897735	*Nitrospina gracilis* strain 3/211 (NR_104821.1), *Nitrospina gracilis* (Atlantic Ocean isolate) (L35504.1)	92%	0	0	0	0	17
**40507**	LT897736	*Nitrospina gracilis* strain 3/211 (NR_104821.1), *Nitrospina gracilis* (Atlantic Ocean isolate) (L35504.1)	92%	0	0	0	0	1
**40516**	LT897737	*Nitrospina gracilis* (Pacific Ocean isolate) (L35503.1)	89%	0	0	0	1	70
**40544**	LT897738	*Nitrospina gracilis* strain 3/211 (NR_104821.1)	91%	0	0	0	0	52
**40554**	LT897739	*Nitrospina gracilis* strain 3/211 (NR_104821.1)	91%	0	0	0	0	2
**40628**	LT897740	*Nitrospina* sp. enrichment culture clone Cb12, North Sea (KC706458.1)	94%	0	4	0	1	269
**40860**	LT897741	*Nitrospina* sp. enrichment culture clone Cb12, North Sea (KC706458.1)	91%	0	0	4	0	1
**40938**	LT897742	*Nitrospina* sp. enrichment culture clone Cb12, North Sea (KC706458.1)	91%	0	0	0	0	1
**41040**	LT897743	*Nitrospina gracilis* strain 3/211 (NR_104821.1), *Nitrospina gracilis* (Atlantic Ocean isolate) (L35504.1)	91%	0	0	0	0	20
**41053**	LT897744	*Nitrospina* sp. enrichment culture clone Cb12, North Sea (KC706458.1)	90%	0	0	0	0	1
**41085**	LT897745	*Nitrospina* sp. enrichment culture clone Cb12, North Sea (KC706458.1)	89%	0	0	0	0	1
**41132**	LT897746	*Nitrospina gracilis* strain 3/211 (NR_104821.1)	88%	0	0	0	0	1
**41153**	LT897747	*Nitrospina* sp. enrichment culture clone Cb12, North Sea (KC706458.1)	94%	0	0	0	0	10
**41232**	LT897748	*Nitrospina* sp. enrichment culture clone Cb12, North Sea (KC706458.1)	92%	0	0	0	0	5
**41346**	LT897749	*Nitrospina gracilis* strain 3/211 (NR_104821.1), *Nitrospina gracilis* (Atlantic Ocean isolate) (L35504.1)	93%	0	0	0	0	11
**41661**	LT897750	*Candidatus* Nitrospira defluvii (JF449941)	81%	0	0	0	0	2
**41782**	LT897751	*Nitrospina* sp. enrichment culture clone Cb12, North Sea (KC706458.1)	90%	0	0	0	0	1

**41817**	LT897752	*Nitrospina* sp. enrichment culture clone Cb12, North Sea (KC706458.1)	93%	0	0	0	0	3
**42008**	LT897753	*Nitrospina* sp. enrichment culture clone Cb12, North Sea (KC706458.1)	94%	0	2	0	3	479
**42066**	LT897754	*Nitrospina* sp. enrichment culture clone Cb12, North Sea (KC706458.1)	93%	0	0	2	2	106
**42116**	LT897755	*Nitrospina* sp. enrichment culture clone Cb12, North Sea (KC706458.1)	94%	0	0	0	0	2
**42164**	LT897756	*Nitrospina gracilis* strain 3/211 (NR_104821.1), *Nitrospina gracilis* (Atlantic Ocean isolate) (L35504.1)	92%	0	5	0	2	13
**42183**	LT897757	*Nitrospina gracilis* strain 3/211 (NR_104821.1)	93%	0	0	2	0	3
**42184**	LT897758	*Nitrospina* sp. enrichment culture clone Cb12, North Sea (KC706458.1)	93%	0	0	0	0	17
**42259**	LT897759	*Nitrospina gracilis* strain 3/211 (NR_104821.1), *Nitrospina gracilis* (Atlantic Ocean isolate) (L35504.1)	91%	0	0	0	0	1
**42279**	LT897760	*Nitrospina gracilis* strain 3/211 (NR_104821.1), *Nitrospina gracilis* (Atlantic Ocean isolate) (L35504.1)	91%	0	0	0	0	1
**42306**	LT897761	*Nitrospina* sp. enrichment culture clone Cb12, North Sea (KC706458.1)	93%	0	0	0	1	13
**42351**	LT897762	*Nitrospina gracilis* strain 3/211 (NR_104821.1), *Nitrospina gracilis* (Atlantic Ocean isolate) (L35504.1)	92%	0	0	0	0	1
**42377**	LT897763	*Nitrospira* sp. enrichment culture clone RTC3 (KC161237.1)	94%	0	0	0	0	1
**42514**	LT897764	*Nitrospina* sp. enrichment culture clone Cb12, North Sea (KC706458.1)	92%	0	0	0	0	2
**42575**	LT897765	*Nitrospina gracilis* strain 3/211 (NR_104821.1), *Nitrospina gracilis* (Atlantic Ocean isolate) (L35504.1)	92%	0	0	0	0	13
**42588**	LT897766	*Nitrospina* sp. enrichment culture clone Cb12, North Sea (KC706458.1)	88%	0	0	0	0	1
**42690**	LT897767	*Nitrospina* sp. enrichment culture clone Cb12, North Sea (KC706458.1)	89%	0	0	0	0	1
**42748**	LT897768	*Nitrospina* sp. enrichment culture clone Cb12, North Sea (KC706458.1)	87%	0	0	0	0	1
**42850**	LT897769	*Nitrospina gracilis* strain 3/211 (NR_104821.1)	88%	0	0	0	0	1
**42936**	LT897770	*Nitrospina* sp. enrichment culture clone Cb12, North Sea (KC706458.1)	92%	0	0	0	0	1
**42989**	LT897771	*Nitrospina gracilis* strain 3/211 (NR_104821.1), *Nitrospina gracilis*	93%	0	0	0	0	1

		(Atlantic Ocean isolate) (L35504.1)						
**43037**	LT897772	*Nitrospina gracilis* strain 3/211 (NR_104821.1), *Nitrospina gracilis* (Atlantic Ocean isolate) (L35504.1)	87%	0	0	0	0	1
**43826**	LT897773	*Nitrospina gracilis* strain 3/211 (NR_104821.1), *Nitrospina gracilis* (Atlantic Ocean isolate) (L35504.1)	89%	0	0	0	1	0
**43860**	LT897774	*Nitrospina gracilis* strain 3/211 (NR_104821.1), *Nitrospina gracilis* (Atlantic Ocean isolate) (L35504.1)	90%	0	0	0	1	0
**43943**	LT897775	*Nitrospina gracilis* strain 3/211 (NR_104821.1)	90%	0	0	0	0	1
**44073**	LT897776	*Nitrospina gracilis* strain 3/211 (NR_104821.1), *Nitrospina gracilis* (Atlantic Ocean isolate) (L35504.1)	89%	0	1	0	0	0
