# Supplementary material for: Diversity and relative abundance of ammonia- and nitrite-oxidizing microorganisms in the offshore Namibian hypoxic zone
Source: PLoS One. 2019 May 21;14(5):e0217136. doi: 10.1371/journal.pone.0217136 (PMC6529010; doi:10.1371/journal.pone.0217136)
Supplement: S4 Table — (PDF) [file pone.0217136.s011.pdf]

S4 Table. Top named or cultured representative(s) based on BLASTN searches and read counts matching the 6 OTUs related to methane-oxidizing bacteria (MOB) of the gammaproteobacterial family Methylococcaceae, and 5 OTUs related to MOB of the Verrucomicrobial family Methylacidiphilaceae.

OTU	GenBank Accession no.	Top named or cultured representative(s)	%ID to match	10m	25m	100m	130m	250m
**5872**	LT900504	*Methylovulum miyakonense* strain HT12 (NR_112920.1), *Methylosoma* sp. TFB (GQ130272.1)	90%	1	0	0	0	0
**9001**	LT900505	*Methylosoma* sp. TFB (GQ130272.1)	94%	1	2	17	11	7
**18621**	LT900506	*Methylobacter tundripaludum* strain SV96 (NR_042107.1)	94%	1	0	0	0	0
**20040**	LT900507	*Methylacidimicrobium tartarophylax* strain 4AC (NR_126314.1), *Methylacidimicrobium fagopyrum* strain 3C (NR_126313.1)	83%	1	0	0	2	0
**20406**	LT900508	*Methylacidiphilum fumariolicum* strain SolV (EF591088.1)	84%	1	0	0	0	0
**23818**	LT900509	*Methylacidiphilum fumariolicum* strain SolV (EF591088.1)	84%	0	1	0	0	0
**33672**	LT900510	*Methylovulum psychrotolerans* strain HV10_M2 (CP022129.1)	97%	0	0	4	0	0
**33885**	LT900511	*Methylacidimicrobium tartarophylax* strain 4AC (NR_126314.1), *Methylacidimicrobium fagopyrum* strain 3C (NR_126313.1)	83%	0	0	5	0	0
**34775**	LT900512	*Methylacidiphilum fumariolicum* strain SolV (EF591088.1)	84%	0	0	1	0	0
**35632**	LT900513	*Methylovulum psychrotolerans* strain HV10_M2 (CP022129.1)	97%	0	0	0	3	0
**35645**	LT900514	*Methylosoma* sp. TFB (GQ130272.1)	94%	0	0	0	1	0
