# Supplementary material for: Diversity and relative abundance of ammonia- and nitrite-oxidizing microorganisms in the offshore Namibian hypoxic zone
Source: PLoS One. 2019 May 21;14(5):e0217136. doi: 10.1371/journal.pone.0217136 (PMC6529010; doi:10.1371/journal.pone.0217136)
Supplement: S5 Table — (PDF) [file pone.0217136.s012.pdf]

S5 Table. Adjusted R (correlation coefficient) and *P* values in comparisons between nitrifier abundances (percentage of total sequences) versus temperature, dissolved O_2 concentration, and N_2O concentration. Determination of correlation between nitrifier abundance and N_2O concentrations at 10 m was ignored as N_2O measurements were from 16–250 m.

**Nitrifier group**	**Environmental parameter**	R_{Adj}	***P*-value**
Ammonia oxidizers (AOA+AOB)	O_2	–0.59	0.17
Ammonia oxidizers (AOA+AOB)	Temperature	–0.57	0.19
Ammonia oxidizers (AOA+AOB)	N_2O	0.62	0.72
Nitrite oxidizers (NOB)	O_2	–0.81	0.06
Nitrite oxidizers (NOB)	Temperature	–0.81	0.06
Nitrite oxidizers (NOB)	N_2O	0.23	0.39
