# Supplementary material for: Diversity and relative abundance of ammonia- and nitrite-oxidizing microorganisms in the offshore Namibian hypoxic zone
Source: PLoS One. 2019 May 21;14(5):e0217136. doi: 10.1371/journal.pone.0217136 (PMC6529010; doi:10.1371/journal.pone.0217136)
Supplement: S6 Table — (PDF) [file pone.0217136.s013.pdf]

S6 Table. Co-occurrence patterns of 55 AOA and 52 NOB OTUs. Singleton occurrences (an OTU represented by only 1 read per depth) were excluded from the analysis. See S1 and S3 Tables for OTU counts at each depth. Exceptions are indicated by parentheses at the depths and explained by the asterisks below.

OTUs co-occrring based on Euclidean distance	Euclidean distance between OTUs	Depths at which OTUs were detected (parentheses with asterisks represent exceptions)
OTU20827(AOA), OTU21374(AOA), OTU22644(AOA), OTU24703(AOA), OTU24879(AOA), OTU25297(AOA), OTU26903(AOA), OTU44192(AOA), OTU2300(NOB), OTU22151(NOB), OTU24503(NOB), OTU25487(NOB), OTU26591(NOB), OTU29130(NOB), OTU30021(NOB), OTU30052(NOB), OTU32166(NOB), OTU33307(NOB), OTU33625(NOB); OTU33781(NOB), OTU33795(NOB), OTU34581(NOB), OTU34680(NOB)	0-0.0005	25m, (100m[A])
OTU21201(AOA), OTU21750(AOA), OTU22714(AOA), OTU24719(AOA), OTU27622(AOA), OTU28846(AOA), OTU30631(AOA), OTU34896(AOA), OTU35335(AOA), OTU36420(AOA), OTU22024(NOB), OTU22710(NOB), OTU23810(NOB), OTU25878(NOB), OTU28610(NOB), OTU29783(NOB), OTU29902(NOB), OTU33909(NOB), OTU37791(NOB)	0-0.0005	25m, (100m[B]), 130m
OTU32932(AOA), OTU35292(AOA), OTU40436(NOB)	0	(25m[C]), (100m[C]), 250m
OTU35512(AOA), OTU37473(AOA), OTU38064(AOA), OTU39088(AOA), OTU41823(AOA), OTU36355(NOB), OTU36916(NOB), OTU36974(NOB), OTU37238(NOB), OTU37423(NOB), OTU38614(NOB)	0	130m, 250m
OTU38487(AOA), OTU35600(NOB), OTU35692(NOB), OTU36148(NOB), OTU36624(NOB), OTU37191(NOB), OTU37295(NOB)	0-0.0008	130m
OTU39630(AOA), OTU39665(AOA), OTU39753(AOA), OTU41600(AOA), OTU41686(AOA), OTU41868(AOA), OTU42202(AOA), OTU42283(AOA),	0	250m

OTU42317(AOA), OTU42454(AOA), OTU43043(AOA), OTU39444(NOB), OTU39524(NOB), OTU40554(NOB), OTU41661(NOB), OTU42116(NOB), OTU42514(NOB)		
OTU9638(AOA), OTU4835(NOB), OTU19055(NOB), OTU17335(NOB)	0-0.0005	10m, 25m
OTU6964(AOA), OTU17034(AOA), OTU17184(NOB), OTU19292(NOB), OTU21004(NOB)	0-0.0008	(10m[D]), 25m, (100m[D]), (130m[D])
OTU31377(AOA), OTU34463(AOA), OTU34531(AOA), OTU37518(AOA), OTU38009(AOA), OTU42787(AOA), OTU33801(AOA), OTU35144(AOA), OTU27139(NOB), OTU34138(NOB)	0-0.0008	25m, (100m[E]), (130m[E]), 250m
OTU34366(AOA), OTU34557(AOA), OTU35170(AOA), OTU34607(NOB)	0-0.0005	25m, (100m[F]), 130m
OTU7793(AOA), OTU9967(AOA)	0	10m
OTU34030(AOA), OTU44176(AOA)	0.0005	25m

Legend:

[A] - All AOA and NOB OTUs in this group were detected only at 25 m, except OTU22644(AOA), OTU25487(NOB), OTU30052(NOB), OTU33307(NOB), OTU33795(NOB), OTU33781(NOB), OTU34581(NOB) and OTU34680(NOB), which were detected at both 25 m and 100 m.

[B] - All AOA and NOB in this group were detected at both 25 m and 130 m, whereas OTU29783(NOB) and OTU33909(NOB) were detected at these depths and additionally at 100 m, and OTU37791(NOB) was detected at 100 m and 130 m only.

[C] - All AOA and NOB OTUs in this group were detected only at 100 m and 250 m, except OTU35292(AOA), which was detected at 25 m and 250 m only.

[D] - OTU4835(AOA) was detected only at 10 m and 25 m, whereas OTU17034 and OTU17184 were detected at these depths as well as 100 m, OTU6964(AOA) was detected at 10 m, 25 m and

130 m, OTU19292(AOA) was detected at 10 m, 25 m, 100 m and 130 m, and OTU21004(NOB) was detected in 25 m, 130 m and 250 m.

[E] - OTU37518(AOA) and OTU38009AOA) were detected at 130 m and 250 m only, OTU34138(AOA) was detected at these depths and at 25 m and 100 m, OTU34531(AOA) and OTU42787(AOA) were detected at 25 m and 250 m only, OTU27139(NOB) was detected at 25 m, 100 m, and 250 m, OTU31377(AOA), OTU34463(AOA), OTU33801(AOA) and OTU35144(AOA) were detected at 25 m, 130 m and 250 m, and OTU35144 was detected at 25 m, 130 m and 250 m.

[F] - All AOA OTUs were detected only at 25 m and 130 m, whereas OTU34607(NOB) was detected at these depths and additionally at 100 m.
